# Characterization of a novel polyextremotolerant fungus, *Exophiala viscosa*, with insights into its melanin regulation and ecological niche

**DOI:** 10.1093/g3journal/jkad110

**Published:** 2023-05-23

**Authors:** Erin C Carr, Quin Barton, Sarah Grambo, Mitchell Sullivan, Cecile M Renfro, Alan Kuo, Jasmyn Pangilinan, Anna Lipzen, Keykhosrow Keymanesh, Emily Savage, Kerrie Barry, Igor V Grigoriev, Wayne R Riekhof, Steven D Harris

**Affiliations:** School of Biological Sciences, University of Nebraska-Lincoln, Lincoln, NE 68588, USA; School of Biological Sciences, University of Nebraska-Lincoln, Lincoln, NE 68588, USA; Roy J. Carver Department of Biochemistry, Biophysics, and Molecular Biology, Iowa State University, Ames, IA 50011, USA; School of Biological Sciences, University of Nebraska-Lincoln, Lincoln, NE 68588, USA; Department of Agronomy and Horticulture, University of Nebraska-Lincoln, Lincoln, NE 68588, USA; US Department of Energy Joint Genome Institute, Lawrence Berkeley National Laboratory, Berkeley, CA 94720, USA; US Department of Energy Joint Genome Institute, Lawrence Berkeley National Laboratory, Berkeley, CA 94720, USA; US Department of Energy Joint Genome Institute, Lawrence Berkeley National Laboratory, Berkeley, CA 94720, USA; US Department of Energy Joint Genome Institute, Lawrence Berkeley National Laboratory, Berkeley, CA 94720, USA; US Department of Energy Joint Genome Institute, Lawrence Berkeley National Laboratory, Berkeley, CA 94720, USA; US Department of Energy Joint Genome Institute, Lawrence Berkeley National Laboratory, Berkeley, CA 94720, USA; US Department of Energy Joint Genome Institute, Lawrence Berkeley National Laboratory, Berkeley, CA 94720, USA; Department of Plant and Microbial Biology, University of California Berkeley, Berkeley, CA 94720, USA; School of Biological Sciences, University of Nebraska-Lincoln, Lincoln, NE 68588, USA; Department of Plant Pathology, Entomology and Microbiology, Iowa State University, Ames, IA 50011, USA

**Keywords:** polyextremotolerant fungi, black yeast fungi, melanin, *Exophiala*, biological soil crusts, microbial ecology, fungi

## Abstract

Black yeasts are polyextremotolerant fungi that contain high amounts of melanin in their cell wall and maintain a primar yeast form. These fungi grow in xeric, nutrient depletes environments which implies that they require highly flexible metabolisms and have been suggested to contain the ability to form lichen-like mutualisms with nearby algae and bacteria. However, the exact ecological niche and interactions between these fungi and their surrounding community are not well understood. We have isolated 2 novel black yeasts from the genus *Exophiala* that were recovered from dryland biological soil crusts. Despite notable differences in colony and cellular morphology, both fungi appear to be members of the same species, which has been named *Exophiala viscosa* (i.e. *E. viscosa* JF 03-3 Goopy and *E. viscosa* JF 03-4F Slimy). A combination of whole genome sequencing, phenotypic experiments, and melanin regulation experiments have been performed on these isolates to fully characterize these fungi and help decipher their fundamental niche within the biological soil crust consortium. Our results reveal that *E. viscosa* is capable of utilizing a wide variety of carbon and nitrogen sources potentially derived from symbiotic microbes, can withstand many forms of abiotic stresses, and excretes melanin which can potentially provide ultraviolet resistance to the biological soil crust community. Besides the identification of a novel species within the genus *Exophiala*, our study also provides new insight into the regulation of melanin production in polyextremotolerant fungi.

## Introduction

**Fig. 1.**
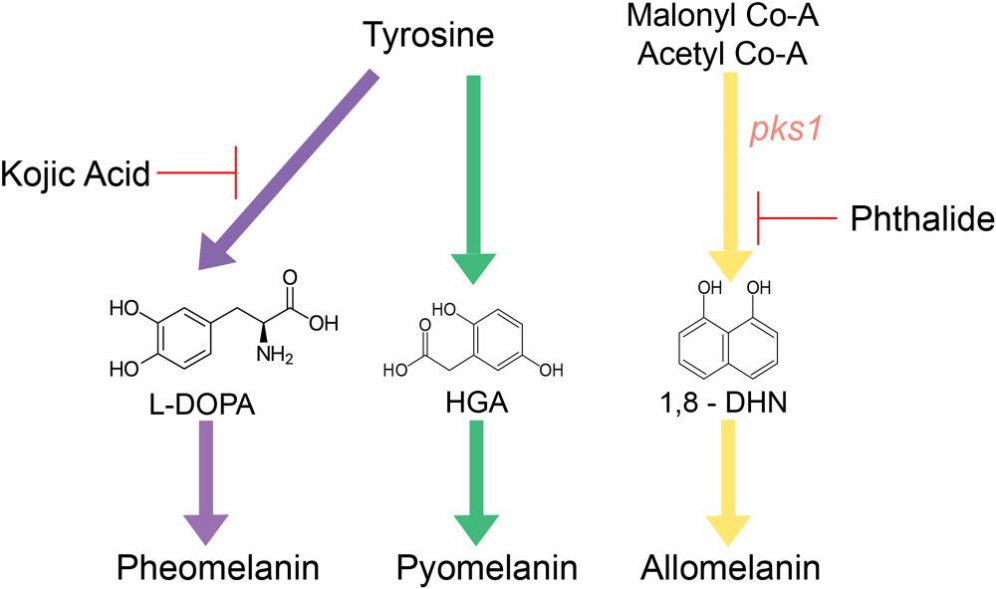
Visual summary of generalized fungal melanin production and the chemicals that block each pathway. Pheomelanin and pyomelanin both use tyrosine as their starting reagent but have different biosynthesis pathways. Pheomelanin uses L-DOPA as a precursor to the final product and pyomelanin uses HMG as a precursor. Allomelanin's starting components are malonyl Co-A and acetyl Co-A, and its immediate precursor is 1,8-DHN. The gene *pks1* is the first gene in the biosynthetic pathway for allomelanin. Kojic acid is a chemical blocker that blocks production of pheomelanin, and phthalide blocks production of allomelanin. Additionally, the chemical precursors of the final melanin product are provided, final melanin compound structures are still unknown and, therefore, the precursors are used in place of melanin structure itself. Detailed descriptions of these pathways can be found in Perez-Cuesta *et al*. (2020) and Płonka and Grabacka (2006).

The production of melanin is a key functional trait observed in many fungi spanning the fungal kingdom (Bell and Wheeler 1986; Zanne *et al.* 2020). The diverse protective functions of melanin (e.g. metal resistance, reactive oxygen species (ROS)- and reactive nitrogen species (RNS) tolerance, and ultraviolet (UV) resistance) underscores its broad utility in mitigating the impacts of stress despite the potential cost of its synthesis (Schroeder *et al.* 2020). Fungi are capable of producing 2 different types of melanin: pheomelanin, allomelanin, and pyomelanin, all of which have their own independent biosynthetic pathways (Pal *et al.* 2014; Perez-Cuesta *et al.* 2020). Allomelanin is formed from the polymerization of 1,8-dihydroxynaphthalene (1,8-DHN), which requires the use of polyketide synthase (PKS) for the initial steps in production (Płonka and Grabacka 2006; Perez-Cuesta *et al.* 2020) (Fig. 1). Pyomelanin and pheomelanin share an initial substrate of Tyrosine, but pheomelanin derives from l-3,4-dihydroxyphenylalanine (L-DOPA), whereas pyomelanin is created via tyrosine degradation (Płonka and Grabacka 2006; Perez-Cuesta *et al.* 2020) (Fig. 1). Allomelanin and pheomelanin are often referred to as DHN-melanin and DOPA-melanin, respectively, given their chemical precursors.

Unfortunately, due to the unique characteristics of melanins and their association with larger biomolecules, we do not know the complete structure of any type of melanin (Cao *et al.* 2021). However, given that we do know their chemical constituents, it is possible to draw some inferences about the relationship between the structure and function of a particular type of melanin. For instance, out of the 3 types of melanin fungi can produce, only pheomelanin has a chemical precursor, 5-CysDOPA, with both nitrogen and sulfur in its structure (Płonka and Grabacka 2006). Notably, all 3 fungal melanins are synthesized via independent pathways, which enables the targeted use of chemical inhibitors to block one pathway without interfering with the others. For example, previous studies have extensively used the chemical melanin blockers kojic acid and phthalide to block pheomelanin and allomelanin, respectively (Pal *et al.* 2014) (Fig. 1). Use of these chemical blockers allowed previous studies to identify the primary melanin biosynthetic pathway employed by individual fungal species (Pal *et al.* 2014).

Polyextremotolerant fungi are a paraphyletic group of melanized fungi that can be divided morphologically into black yeast and microcolonial/meristematic fungi types (Gostinčar *et al.* 2012). These 2 subtypes are distinct in their morphology; black yeast fungi are primarily yeasts but can be dimorphic, whereas microcolonial fungi are typically filamentous, pseudohyphal, or produce other unique morphologies such as spherical cells (De Hoog *et al.* 2003; Gostinčar *et al.* 2011; Ametrano *et al.* 2017). However, all polyextremotolerant fungi share the capacity to produce melanin, which presumably accounts for much of their polyextremotolerance. Most polyextremotolerant fungi are in the subdivision Pezizomycotina, residing mainly within Eurotiomycetes and Dothidiomycetes (Gostinčar *et al.* 2009).

Melanin is arguably the defining feature of polyextremotolerant fungi given that they form unmistakably black colonies. Because of its structure and association with the cell wall, melanin imbues polyextremotolerant fungi with resistance to multiple forms of stress. The most commonly known is UV light resistance, as melanin absorbs light in the UV part of the spectrum (Kobayashi *et al.* 1993). However, melanin is also capable of quenching ROS and RNS, providing tolerance to toxic metals, reducing desiccation, and enabling the use of ionizing radiation as an energy source (Płonka and Grabacka 2006; Dadachova *et al.* 2007; Gessler *et al.* 2014; Cordero and Casadevall 2017; Zanne *et al.* 2020). Collectively, these functions of melanin are thought to enhance the ability of polyextremotolerant fungi to colonize habitats that are otherwise inhospitable to most forms of life.

Polyextremotolerant fungi tend to occupy extreme niches such as rock surfaces, external brick and marble walls, soil surfaces, and even the inside of dishwashers (Gostinčar *et al.* 2009; Zupančič *et al.* 2016). Characteristic features of these environments include the relative paucity of nutrients and the frequent presence of a community biofilm consisting of photosynthetic organisms and/or bacteria (Gostinčar *et al.* 2012). Strikingly, these microbes are rarely found alone in their habitats, which suggests that multispecies interactions between fungi, bacteria, and photosynthetic organisms underlie the formation and function of these communities. That is, the ability of polyextremotolerant fungi to successfully adapt to their niche must depend on complex yet poorly understood interactions with other microbes.

We have isolated 2 polyextremotolerant fungi from a biological soil crust (BSC) in British Columbia, Canada. These novel fungi are of the genus *Exophiala*, which has previously been found in BSCs (Bates *et al.* 2006). Biological soil crusts are unique dryland biofilms that form on the surface of xeric desert soils where little to no plants are able to grow (Belnap *et al.* 2001; Belnap 2003). They are notable for their extensive cyanobacteria population, which seeds the initial formation of all BSCs and creates the main source of nitrogen for the rest of the community (Belnap 2002). Once the initial crust is established, it is then inundated with a consortium of bacteria, fungi, algae, archaea, lichens, and mosses (Bates *et al.* 2010; Lan *et al.* 2012; Maier *et al.* 2016). This community is a permanent fixture on the land they occupy unless physically disturbed, much like other biofilms (Belnap and Eldridge 2001; Donlan and Costerton 2002). As a result of the arid conditions to which BSCs are often exposed, their resident microbes are constantly exposed to extreme abiotic factors that must be countered to enable survival (Bowker *et al.* 2010). Some of these abiotic extremes are UV radiation (especially at higher altitudes and latitudes) (Bowker *et al.* 2002), desiccation and osmotic pressures (Rajeev *et al.* 2013), and temperature fluctuations both daily and annually (Belnap *et al.* 2001; Bowker *et al.* 2002; Pócs 2009). Accordingly, microbes that reside within BSCs must, therefore, possess adaptations that allow them to withstand these abiotic extremes.

An extensive amount of research has been dedicated to certain members of the BSC community, but one such less-studied microbe has been the “free-living” fungal taxa. These fungi are nonlichenized (Teixeira *et al.* 2017) yet are still thriving in an environment where there are no plants to infect or decompose (Belnap and Lange 2003), and no obvious source of nutrients aside from contributions of the other members of the BSC community (Belnap and Lange 2003). This would imply that even though these fungi are not lichenized per se, they would have to engage in lichen-like interactions with the BSC community to obtain vital nutrients for proliferation. While previous researchers have postulated the idea of transient interactions between nonlichenized fungi and other microbes (Gostinčar *et al.* 2012; Hom and Murray 2014; Grube *et al.* 2015), confirmation of this interaction, especially in BSCs, is potentially challenging given their taxonomic complexity.

Despite the importance of microbial interactions in enabling the successful formation of BSCs in a niche characterized by poor nutrient and water availability, the fungal components of BSCs and their relative functions within the interaction network remain poorly understood. Here, we combine genome sequencing with computational tools and culture-based phenotyping to describe 2 strains of a new species of black yeast fungi associated with BSCs. We report on their carbon and nitrogen utilization profiles, stress responses, and lipid accumulation patterns. In addition, we characterize their capacity for melanin production and generate valuable insight into mechanisms that might be involved in regulating the synthesis of these compounds so that melanin production contributes to the BSC community.

## Methods

### Fungal strains and media

Two strains of a novel species of fungi are described here: *Exophiala viscosa* JF 03-3F Goopy CBS 148801 and *E. viscosa* JF 03-4F Slimy CBS 148802. *E. viscosa* is typically grown in malt extract medium (MEA; see Table 1 for media recipe) at room temperature in an Erlenmeyer flask at 1/10th the volume of the flask, shaking at 160 rpm. Additional strains used in this study were *Saccharomyces cerevisiae* ML440 and BY4741, and *E. dermatitidis* wild-type (WT) strain ATCC 34100. *S. cerevisiae* strains are grown in yeast peptone dextrose medium (YPD; Table 1 for media recipe), and *E. dermatitidis* is grown in MEA.

**Table 1.** Medias used and their compositions.

Media name	Acronym	Composition (L^−1^)
Malt extract agar glucose	MAG	20 g dextrose 20 g malt extract (Sigma 70167) 2 g peptone (Fisher BP1420) 1 mL Hutner's TE 1 mL vitamin mix 15 g agar
Malt extract agar	MEA	20 g dextrose 20 g malt extract 2 g peptone 15 g agar
Minimal	MN	10 g dextrose 50 mL 20× nitrate salts 1 mL Hutner's TE
Minimal + vitamins	MNV	10 g dextrose 50 mL 20× nitrate salts 1 mL Hutner's TE 1 mL vitamin mix
Minimal + *N*-acetyl glucosamine	MN + NAG	10 g dextrose 50 mL 20× nitrate salts 1 mL Hutner's TE 4.74 g *N*-acetyl glucosamine (21.43 mM)
Potato dextrose agar	PDA	24 g BD Difco potato dextrose broth powder 15 g agar (if not in powder)
Spider	Spider	20 g nutrient broth 20 g mannitol 4 g K_2_HPO_4_ 27 g agar pH adjusted to 7.2 with NaOH
Yeast extract peptone dextrose	YPD	20 g dextrose 20 g peptone 10 g yeast extract 20 g agar
V8	V8	200 mL V8 juice 2 g CaCO_3_ 15 g agar
Tyrosine media	Tyr	20 g dextrose 10 g peptone 1 g yeast extract 20 g agar

Additives	Volume	Composition
20× nitrate salts/MN salts	1 L	120 g NaNO_3_ (remove for “MN salts”) 10.4 g KCl 10.4 g MgSO_4_-7H_2_O 30.4 g KH_2_PO_4_
Hutner's TE	100 mL	2.2 g ZnSO_4_-7H_2_O 1.1 g H_3_BO_3_ 0.5 g MnCl_2_-4H_2_O 0.5 g FeSO_4_-7H_2_O 0.17 g CoCL_2_-6H_2_O 0.16 g CuSO_4_-5H_2_O 0.15 g Na_2_MoO_4_-2H_2_O 5 g EDTA (disodium salt)
Vitamin mix	100 mL	10 mg biotin 10 mg pyridoxin 10 mg thiamine 10 mg riboflavin 10 mg p-aminobenzoic acid 10 mg nicotinic acid

## Fungal identification, genomics, and phylogenetics

### Fungal isolation and identification methods

Fungi were isolated from a BSC in British Columbia (B.C.), Canada, Latitude: 52.94997°, Longitude: −119.41919°. Soil samples were taken from the top 2 cm of the BSC. A 0.1 g portion of the crust was re-suspended in 1 mL of water, ground with a sterile micropestle, and diluted with a dilution factor (DF) of 10 till they reached 10,000× dilution. Each dilution was then spread out onto 2 different MEA petri plates containing either no antibiotics or a plate containing: ampicillin (100 mg/L final concentration), chloramphenicol (50 mg/L), gentamycin (10 mg/L), and cycloheximide (100 mg/L). The plates were then incubated in a Percival light incubator at 23°C with a 12 hr light/dark cycle and examined daily using a dissection microscope to check for small black colonies. Once a potential black colony was seen, half of it was removed and transferred to a new MEA petri plate with no antibiotics. It was vital to examine the plates daily because even in the presence of antibiotics many unwanted fast-growing microbes would grow on the plates and cover up the slower-growing polyextremotolerant fungal colonies. Once a pure culture of each isolate was grown up (approximately 2 weeks), they were preserved in 30% glycerol and stored in the −80°C freezer.

DNA sequencing of amplified internal transcribed spacer (ITS) sequences was used to initially identify the isolates. DNA was extracted using the Qiagen DNeasy Powersoil DNA extraction kit. Primers used to isolate the ITS region were: ITS1- (5′-TCC GTA GGT GAA CCT GCG G-3′) and ITS4- (5′-TCC TCC GCT TAT TGA TAT GC-3′) (White *et al.* 1990). A BioRad MJ Mini Personal Thermal Cycler was used, with the program set as (1) 95°C for 5:00 (5 minutes), (2) 94°C for 0:30 (30 seconds), (3) 55°C for 0:30, (4) 72°C for 1:30, (5) Return to Step 2 35 times, (6) 72°C for 5:00. Resulting PCRs were then checked via gel electrophoresis in 1% agar run at 80 V for 1 hr. Isolated ITS regions were sequenced using the Eurofins sequencing facility with their in-house ITS primers as the sequencing primers, and the sequences were subsequently identified using the basic local alignment search tool (BLAST) of the National Center for Biotechnological Information (NCBI) database to look for potential taxa matches.

### DNA extraction and RNA extraction for whole genome sequencing and annotation

A cetyltrimethylammonium bromide (CTAB) based DNA extraction method was performed to obtain high molecular weight DNA for whole genome sequencing. The DNA extraction method used was derived from (Cubero *et al.* 1999). Changes to the original protocol include: (1) switching polyvinylpolypyrrolidone for the same concentration of polyvinylpyrrolidone, (2) use of bead beating tubes with liquid nitrogen-frozen cells and the extraction buffer instead of a mortar and pestle for breaking open the cells, and (3) heating up the elution buffer to 65°C before eluting the final DNA. These changes were made to optimize the protocol for liquid-grown fungal cells instead of lichen material. Cells for the DNA extraction were grown up in 25 mL of liquid MEA in 250 mL Erlenmeyer flasks for 5 days at room temperature with 160 rpm shaking. 1 mL of the grown cells was used for the DNA extraction after washing with water twice. Protocol for the CTAB extraction method can be found at: protocols.io at: dx.doi.org/10.17504/protocols.io.n92ldp9qol5b/v1.

RNA was obtained using the Qiagen RNeasy Mini Kit (Cat. No. 74104). Cells were grown in 25 mL of 3 different liquid media types (MEA, YPD, and minimal media with vitamins added (MNV); see Table 1) in 250 mL Erlenmeyer flasks at room temperature for 5 days (as described above), and 1–2 mL of cells were used for the RNA extraction. Cells were washed with DEPC-treated water and flash-frozen in liquid nitrogen in 1.5 mL microcentrifuge tubes. RNA extraction was then performed according to the methods by the RNeasy kit.

### Genome assembly and annotation

Both genomes and transcriptomes were sequenced using Illumina technology. For transcriptomes, a plate-based RNA sample prep was performed on the PerkinElmer SciClone next-generation sequencing (NGS) robotic liquid handling system using Illumina's TruSeq Stranded mRNA high throughput sample prep kit utilizing a poly-A selection of mRNA following the protocol outlined by Illumina in their user guide: https://support.illumina.com/sequencing/sequencing_kits/truseq-stranded-mrna.html, and with the following conditions: total RNA starting material was 1 μg per sample and 8 cycles of PCR was used for library amplification. The prepared libraries were quantified using KAPA Biosystems’ NGS library qPCR kit and run on a Roche LightCycler 480 real-time PCR instrument. The libraries were then multiplexed and prepared for sequencing on the Illumina NovaSeq sequencer using NovaSeq XP v1 reagent kits, and S4 flow cell, following a 2 × 150 indexed run recipe.

BBDuk was used for trimming and filtering sequencing reads (https://sourceforge.net/projects/bbmap/). Raw reads were evaluated for artifact sequence by k-mer matching (k-mer = 25), allowing 1 mismatch, and detected artifacts were trimmed from the 3′ end of the reads. RNA spike-in reads, PhiX reads, and reads containing any Ns were removed. Quality trimming was performed using the phred trimming method set at Q6. Finally, following trimming, reads under the length threshold were removed (minimum length 25 bases or 1/3 of the original read length—whichever is longer). Filtered reads were assembled into consensus sequences using Trinity ver. 2.3.2 (Grabherr *et al.* 2011). Specific Trinity options that were utilized were: –jaccard_clip, –run_as_paired, –min_per_id_same_path 95, –normalize_reads.

For genomes, DNA library preparation for Illumina sequencing was performed on the PerkinElmer Sciclone NGS robotic liquid handling system using the Kapa Biosystems library preparation kit. 200 ng of sample DNA was sheared to 300 bp using a Covaris LE220 focused-ultrasonicator. The sheared DNA fragments were size selected by double-solid phase reversible immobilization (SPRI) and then the selected fragments were end-repaired, A-tailed, and ligated with Illumina compatible sequencing adaptors from Integrated DNA technologies (IDT) containing a unique molecular index barcode for each sample library. The prepared libraries were quantified using KAPA Biosystems’ NGS library qPCR kit and run on a Roche LightCycler 480 real-time PCR instrument. The quantified libraries were then multiplexed with other libraries, and the pool of libraries was then prepared for sequencing on the Illumina HiSeq sequencing platform utilizing a TruSeq paired-end cluster kit, v4, and Illumina's cBot instrument to generate a clustered flow cell for sequencing. Sequencing of the flow cell was performed on the Illumina HiSeq2500 sequencer using HiSeq TruSeq SBS sequencing kits, v4, following a 2 × 150 indexed run recipe.

An initial assembly of the target genome was generated using VelvetOptimiser version 2.1.7 (3) with Velvet version 1.2.07 (Zerbino and Birney 2008) using the following parameters; “–s 61 –e 97 –i 4 –t 4, –o “-ins_length 250 -min_contig_lgth 500””. The resulting assembly was used to simulate 28× of a 2 × 100 bp 3000 ± 300 bp insert long read mate-pair library with wgsim version 0.3.1-r13 (https://github.com/lh3/wgsim) using “-e 0 -1 100 -2 100 -r 0 -R 0 -X 0 -d 3000 -s 30”. 25× of the simulated long read mate-pair was then co-assembled together with 125× of the original Illumina filtered fastq with AllPathsLG release version R49403 (Gnerre *et al.* 2011) to produce the final nuclear assembly. A simulated long read assembly was required because AllpathsLG requires both a long read and short read insert library and having both types of libraries was beyond the cost of this project. Therefore, a subsample of the original Illumina short read inserts and the newly simulated long read insert data was fed into AllpathsLG. This methodology was shown to produce superior assemblies over other assemblers with a single short insert library. The genome was then annotated using the Joint Genome Institute (JGI) annotation pipeline (Grigoriev *et al.* 2014). The JGI annotation pipeline (1) detects and masks repeats and transposable elements, (2) predicts genes using a variety of methods, (3) characterizes each conceptually translated protein using a variety of methods, (4) chooses a “best” gene model at each locus to provide a filtered working set, (5) clusters the filtered sets into draft gene families, and (6) creates a JGI Genome Portal with tools for public access and community-driven curation of the annotation (Grigoriev *et al.* 2012; Kuo *et al.* 2014).

The assembly scaffolds were masked by RepeatMasker (http://www.repeatmasker.org/) using both the manually curated RepBase library (Jurka 2000) and a library of de novo repeats discovered by RepeatScout (Price *et al.* 2005). Transcript-based gene models were derived by assembling Illumina-sequenced RNA reads into contigs which were then modeled on genomic scaffolds using EST MAP (http://www.softberry.com) and COMBEST (Zhou *et al.* 2015). Protein-based gene models were predicted using GeneWise (Birney and Durbin 2000) and FGENESH+ (Salamov and Solovyev 2000) seeded by BLASTx alignments of genomic sequence against sequences from the NCBI nonredundant protein set nr (Altschul *et al.* 1990). Ab initio gene models were predicted using GeneMark-ES (Ter-Hovhannisyan *et al.* 2008) and FGENESH, the latter trained on a set of putative full-length transcripts and reliable protein-based models. GeneWise models were completed using scaffold data to find start and stop codons. RNA contig alignments to the genome were used to verify, complete, and extend the gene models.

All predicted gene models were functionally annotated by the JGI Annotation Pipeline using InterProScan (Zdobnov and Apweiler 2001), BLASTp alignments against nr, and highly curated databases such as SwissProt (Bairoch *et al.* 2005), KEGG (Ogata *et al.* 1999), and Pfam (Bateman *et al.* 2004). KEGG hits were used to map enzyme commission (EC) numbers (Bairoch 2000); InterPro, KEGG, and SwissProt hits were used to map GO terms (Ashburner *et al.* 2000). In addition, predicted proteins were annotated according to KOG classification (Koonin *et al.* 2004). Protein targeting predictions were made with signalP (Nielsen *et al.* 1999) and trans membrane prediction using Hidden Markov Models (TMHMM) (Krogh *et al.* 2001). Because the gene-prediction steps usually generated multiple gene models per locus, the Pipeline selected a single representative gene model for each locus based on protein similarity, RNA coverage, and functional annotation, leading to a filtered set of gene models for downstream analysis. All filtered model proteins in a genome were aligned\to each other and to those of other related genomes by blastp. The alignment scores were used as a distance metric for clustering by MCL (http://www.micans.org/mcl/) into a first draft of candidate multigene families for each genome. Percent GC content for the genomes of various *Exophiala* spp. was determined by uploading each fungi's “Masked assembly scaffolds” file from JGI's Mycocosm to the program Geneious (Kearse *et al.* 2012), and the % GC output displayed was used as the genomic GC content.

All genomic scaffolds, RNA contigs, gene models and clusters, and functional predictions thereof, may be accessed through MycoCosm Herpotrichiellaceae PhyloGroup Portal (https://mycocosm.jgi.doe.gov/Herpotrichiellaceae/) and the MycCosm Black Yeasts EcoGroup Portal (https://mycocosm.jgi.doe.gov/black_yeasts/). The assemblies and annotations of both genomes are available at the fungal genome portal MycoCosm (Grigoriev *et al.* 2014); https://mycocosm.jgi.doe.gov/EurotioJF033F_1/EurotioJF033F_1.home.html; https://mycocosm.jgi.doe.gov/EurotioJF034F_1/EurotioJF034F_1.home.html) and in the DDBJ/EMBL/GenBank repository under accessions JALDXI000000000 and JALDXH000000000.

### Phylogenetic analyses

Our single-copy homologous protein sequence approximate maximum likelihood phylogenetic tree was constructed from the protein sequences of 24 taxa which represent a sampling of the family Herpotrichielleaceae in which *E. viscosa* JF 03-3F and JF 03-4F reside. We created this tree using the pipeline described by Kuo *et al.* (2014). To obtain the largest set of data for phylogenetic analysis available, we defined homologous proteins across all 24 genomes using best bidirectional blast (BBB) pairs via blastp across all proteins of all the genomes from their FilteredModels or ExternalModels files available on Mycocosm, with an e-value 1e-05 cutoff for BBBs. Clusters were compiled using in-house JGI scripts. If there are e.g. N genomes, we first compute BBBs individually for each pair of genomes (in total N*(*N*–1)/2 pairs) and then use BBBs as an input computed for each pair of genomes. Another script finds BBBs that are present in all *N* genomes, so these are sets of *N* genes (one from each species or strain) that are BBB to each other in all *N* genomes. The resulting defined homologous proteins across all 24 genomes were then used as our sequences for the rest of the pipeline. These sequences were aligned with Mafft v7.123b with no alterations (Katoh *et al.* 2002). That alignment was then trimmed using Gblocks 0.91b with options –b4 = 5 –b5 = h to have a minimum of 5 positions per block and allow up to half of those to be gaps (Castresana 2000). The resulting trimmed alignment was then input into FastTree version 2.1.5 SSE3 with options -gamma -wag (Price *et al.* 2009). The tree file was then input into FigTree for visual optimization and edited in Adobe Illustrator to highlight the new species.

The combined Bayesian and maximum likelihood concatenated gene phylogenetic trees were constructed using 18S rDNA, 28S rDNA, and ITS sequences of *Exophiala, Pheoannellomyces,* and *Endocarpon* species available from NCBI GenBank. Accession numbers and strains of fungi used for phylogenetic analyses are listed in Table 2. Specific fungal species and genes were chosen based on relatedness to our new fungus via BLAST alignment and the availability of all marker genes needed for analyses. The marker gene *RPB1* was also concatenated to the others tested for all species except *Phaeoannellomyces elegans* and *Exophiala crusticola* which did not have an available *RPB1* sequence in NCBI. Alignments of individual gene sets were performed using the program MEGA X (Kumar *et al.* 2018). The alignment of the sequences was done using a MUSCLE aligner (Edgar 2004). Gap open was set to −400.00, gap extends set to 0.00, max memory was set to 2,048 MB, max iterations was 16, cluster method used was UPGMA, and minimal diagonal length was 24. Trimming was done by hand, first by removing any excess ends, then only essential gaps that formed where 1- or 2-taxa contained inserted nucleotides were removed. This allowed for the parts of the ITS and *RPB1* regions that remain highly variable even across the same genus to be kept, whereas when trimming was attempted with Gblocks with all least stringent options selected, only the most conserved regions were maintained resulting in improper phylogenies (e.g. *Exophiala eucalyptorum* 121,638 did not cluster with other *Exophiala* species but was eventually removed from the tree for clarity). The alignments were then uploaded to Geneious Prime 2023.0.4 (Kearse *et al.* 2012). Individual gene sets were then concatenated using the concatenate alignments tool.

**Table 2.** List of NCBI accession numbers of genes and strains of each fungal species used for concatenated phylogenetic analyses. If alternative strain was used for a gene the strain is listed under the accession number.

Fungal species and predominant strain used	18S	ITS	rpb1	28S
*Exophiala aquamarina CBS 119918*	NG 062075	NR 111626	XM 013403745	KX712347
				Sutton R-3685
*Exophiala bergeri CBS 353.52*	NG 062764	NR 165997	FJ358371	NG_059199
			PW2468	
*Exophiala cancerae CBS 120420*	KF155198	NR 137766	JX498928	MH874540
			PW2463	CBS 115142
*Exophiala castellanii CBS 158.58*	NG 062076	NR 121460	FJ358372	NG_070513
				
*Exophiala dermatitidis CBS 207.35*	X79312	NR 121268	JX498937	NG_059225
			PW 2643	
*Exophiala jeanselmei CBS 507.90*	NG 062765	NR 111129	JX498921	NG_070514
			CBS 677.76	
*Exophiala lecanii-corni CBS 123.33*	NG 062766	NR 145351	JX498934	NG_059200
			PW2534	
*Exophiala nigra CBS 535.94*	NG 062119	MH862481	FJ358375	NG_059253
			dh 12296	
*Exophiala nishimurae CBS 101538*	NG 063073	NR 137092	JX498925	KX712351
				
*Exophiala oligosperma CBS 725.88*	AY554287	NR 111134	JX498935	NG_059201
			PW2535	
*Exophiala pisciphila CBS 537.73 rpb1*	JN856018	NR 121269	DQ840556	DQ823101
				AFTOL-ID 669
*Exophiala salmonis CBS 157.67*	NG 061119	NR 121270	EF413610	AY213702
			no strain listed	
*Exophiala sideris CBS 121828*	NG 062072	NR 111553	Mycococsm BLAST	MH875856
				CBS:127096
*Exophiala spinifera CBS 899.68*	KF155201	NR 111131	JX498922	KF155182
	CBS 101534		CBS 101644	CBS 101534
*Exophiala xenobiotica CBS 118157*	KF155202	NR 111203	JX498920	XR_001230708
			CBS 117753	
*Endocarpon pusillum AFTOLID 2279*	EF689837	JQ927447	EF689756	EF643754
*Phaeoannellomyces elegans CBS 101597*	X80708	NR 155687	N/A	KF928507

Phylogenetic estimates were performed using Bayesian inference via the Mr. Bayes plug-in ver. 2.2.4 in Geneious (Huelsenbeck and Ronquist 2001; Ronquist and Huelsenbeck 2003). For the Bayesian analysis, we used metropolis-coupled MCMC and the general time reversible (GTR) model with gamma distribution with invariant sites (Γ + I) the GTR + Γ + I model, with 5 gamma categories. We conducted the run with 4 heated chains at a temp, of 0.2 for 500,000 generations, sampling trees every 1,000 generations, with a burn length of 10,000, and random seed of 20,512. The resulting phylogenetic tree was then used as the seed tree for Maximum Likelihood analyses. A maximum likelihood phylogenetic tree was created in MEGA X using the maximum likelihood statistical method and the bootstrap method for the test of phylogeny with 1,000 bootstrap replications. The substitution type used was nucleotide and not a protein-coding sequence of DNA, and the model used for the substitution was the GTR + Γ + I with 5 discrete gamma categories. Gaps were included in the data treatment as many individual gaps are created in ITS and *RPB1* sequences after alignment in the variable regions. For tree inference options we used the nearest-neighbor-interchange heuristic method and had the initial tree be automated under the default settings-neighbor joining (NJ)/bioNJ, the resulting Bayesian tree was used as the initial tree file, and no branch swap filter. The resulting tree in newick (.nwk) form with branch lengths and bootstrap values was then imported into the interactive tree of live program ver. 6.6 for visualization, and edited in Adobe Illustrator to add combined posterior probabilities and bootstrap values and highlight the new species (Letunic and Bork 2021).

### Assemble species by automatic partitioning for DNA-based species delimitation

We used assemble species by automatic partitioning (ASAP) to confirm our species delimitation that was observed in our phylogenetic analyses (Puillandre *et al.* 2021). We used the web-based version of ASAP (https://bioinfo.mnhn.fr/abi/public/asap/) using the Kimura (K80) model on our aligned ITS-only, 18S-28S-ITS gene concatenation alignment, and our 18S-28S-ITS-*RPB1* gene concatenation alignment. The resulting lowest ASAP score (smallest *P*-value and largest relative barcode gap) was chosen to be the most likely delimitation of species of that dataset.

### Mating type locus identification

Mating loci for *E. viscosa* JF 03-3F and *E. viscosa* JF 03-4F were determined using the methods described by (Teixeira *et al.* 2017). Genes known to flank the MAT loci of most Chaetothyriales species include *APN2*, *SLA2*, *APC5*, and *COX13*. The protein sequences of these genes from *Aspergillus nidulans* were used to BLASTp against the genomes of the new fungi. These gene sequences were obtained from Aspergillus Genome Database (now called FungiDB https://fungidb.org/) and protein-protein BLASTed using JGI's Mycocosm. Once the genes were found, analysis of upstream and downstream flanking genes was performed until the mating gene MAT1-1 was located. Genes close to MAT1-1 and within the span of genes listed above were considered part of the MAT locus.

### Synteny dot plot formation

To further elucidate the similarities between our 2 strains and their relatedness to *E. sideris*, we created synteny dot plots using their genome assemblies. The genome assembly scaffolds of *E. viscosa* JF 03-3F and *E. viscosa* JF 03-4F were used to create the synteny dot plots comparing these 2 genomes. The assembled genome of *E. sideris* was downloaded from NCBI accession number KN846951. Dot plots were created using the program D-genies (https://dgenies.toulouse.inra.fr/) (Cabanettes and Klopp 2018). Settings used to create the dot plots were: aligner: Minimap2 v2.24, and repeatedness: many repeats. The resulting dot plots then had their contigs sorted to create an aligned figure. Final figures were created in Adobe Illustrator using the.svg file output.

### Phenotyping experiments

Phenotyping experiments of E. viscosa JF 03-3F and E. viscosa JF 03-4F were performed to characterize the new species’ capabilities and differences between the strains. Experiments performed were carbon utilization, nitrogen utilization, UV resistance, metal resistance, growth temperature range, budding patterns, lipid profiles, and growth capabilities on various fungal media as described below.

#### Budding pattern determination

Protocols for observing the budding patterns of these new strains were derived from methods in Mitchison-Field *et al.* (2019) (Supplementary Fig. 1). A 1:1:1 ratio by weight of Vaseline, Paraffin, and Lanolin (VALAP) was combined in a glass bottle and heated to 115°C to melt completely and kept at room temperature for later use. Heated solid MEA was aliquoted into a 50 mL tube for agar slab making. Isolates were grown in liquid MEA for 5 days before inoculation of slides. First, the VALAP was brought back up to 115°C to melt completely for application. Then 5 μL of the 5-day-old cells were diluted in 995 μL of liquid MEA. Agar slabs of MEA were made by microwaving the 50 mL tube of solid MEA until it melted, then pipetting 1 mL of the hot agar into a 1 cm × 2 cm mold formed out of cut strips of silicone and laid down in a sterile petri dish. This agar slab was allowed to solidify and was then cut in half to be 1 cm × 1 cm. Both the microscope cover slip and the microscope slide were wiped down with 70% ethanol to ensure clean and sterile growth conditions for the cells. 6 μL of the diluted cells were pipetted onto the center of the sterile slide, then one square of the agar slab was carefully placed on top of the cells in media, 8 μL of MEA was pipetted onto the top of the agar slab, and the coverslip was placed over the agar slab. Using a small paintbrush, the melted VALAP was carefully painted onto the gap between the coverslip and the microscope slide to seal off the coverslip. Finally, a 23-gauge needle was used to poke holes in the solidified VALAP to allow for gas exchange. The slide was then placed with the microscope slide facing down onto the inverted microscope EVOS fl set to the 40× objective. Once an adequate number of cells was observed in a frame, the cells were allowed to settle for 2 hours before imaging began. Images were then automatically taken every 30 mins for 72 hours. Videos of the budding pattern were created using Adobe Premiere Pro.

#### Growth of *E. viscosa* JF 03-3F and *E. viscosa* JF 03-4F on different media

Nine different fungal media were used to observe the growth of these novel fungi. These media have been used for identification purposes and will be useful for future identification of this species from other locations. Media used in this experiment were: malt extract agar glucose (MAG), MEA, minimal media (MN), MNV, MN + *N*-acetyl glucosamine (NAG), potato dextrose agar (PDA), spider, YPD, YPD + trace elements (TE), and V8 (Table 1). Both isolates were first grown in 25 mL of liquid MEA in a 250 mL Erlenmeyer flask at room temperature, shaking at 200 rpm for 5 days. Then 1 mL of each fungus was aliquoted and washed 3 times with water. Washed cells were then applied to the media in 3 ways: 5 μL spotting (pipetting 5 μL of cells onto the plate), toothpick poking (poking a sterile toothpick tip into the suspended cells and then onto a plate), and metal loop streaking (placing sterile metal loop into suspended cells, then spreading cells onto a plate in a decreasing manner). Cells were then allowed to grow on the plates for 14 days. This provided us with different plating techniques that could potentially yield different morphologies. Descriptions of colony color morphologies follow color nomenclature from Ridgway (1912).

#### Carbon utilization

The carbon utilization of each isolate was determined using a BioMerieux ID C32 carbon utilization strip (Cat. No. 32200-1004439110). These strips have 30 different carbon sources in individual wells, one well with no carbon source for negative control, and one well with esculin ferric citrate. The esculin (β-glucose-6,7-dihydroxycoumarin) ferric citrate assay is a colorimetric assay originally used to identify Enterobacteria via the production of a dark color in positive cultures for esculin degradation (Edberg *et al.* 1977) and was co-opted to identify *Cryptococcus neoformans* (Edberg *et al.* 1980). Therefore, the dark coloration that forms is due to the nature of the colorimetric assay, not due to extreme amounts of melanized fungal growth. The inoculated strips were kept in a plastic box container with a lid and lined with moist paper towels to reduce drying. The initial inoculum of cells was prepared in 25 mL of MEA shaking at room temp for 5 days. Inoculation of the strips was done according to the instructions provided by the vendor, and each strain was inoculated in 3 separate strips for triplicate replication. Cells were diluted to the kit requirement of McFarland standard #3 (McFarland 1907) before starting the inoculum. Growth in the ID strip lasted 10 days before evaluation. Growth in each well was observed and evaluated by eye. Each well was compared to the negative control (no carbon) and the positive control (dextrose). Nuances of the fungal growth on individual carbon sources required a more gradual scale, and so scores were adjusted to form a range of 1–5 to allow for more accurate average calculation between the 3 replicates. For this scale: “1” was no growth or equal to the kits “−”, “5” was the most growth and equivalent to the kit's “+”; numbers in between allowed us to average out the replicates to ensure we had a sliding scale of utilization rather than multiple variable “V” utilizations without a clear idea of how variable the utilization was. Since triplicates were performed, we averaged the growth results in each carbon source and then determined the fungus’ carbon utilization profile from the averages. The scale used goes as follows: no growth “−” = 1.0–1.9, variable growth “v” = 2.0–3.6, growth “+” = 3.7–5.0.

#### Wavelength for optical density determination

The proper wavelength for optical density (OD) readings of these new strains was determined by creating a standard curve. We performed a full spectrum analysis of serially diluted cells of these fungi at the end of their exponential growth phase. Cells were allowed to grow in 25 mL of MEA in a 250 mL Erlenmeyer flask for 5 days. 200 μL of culture was removed, washed 3× with water, and diluted 1:1 in MEA (100 μL of cells to 100 μL of MEA) 9 times to reach a maximum dilution of 1:256 (DF = 512; % dilution = 0.1953%). OD was measured at wavelengths from 300 nm–700 nm in 10 nm increments. Then the *R*^2^ values for: OD to percent dilution at 25% dilution (DF = 4) and below (25% ≥ % Dilu.), OD to percent dilution at 50% dilution (DF = 2) and below (50% ≥ % Dilu.), OD to cell count at 2.19 × 10^6^ cells/mL (DF = 8) and below, OD to cell count at 4.38 × 10^6^ cells/mL (DF = 4) and below, and the accuracy that the ODs quantified the actual percent dilutions at 12.5% dilution (DF = 8) and below (i.e. is the OD at 12.5% dilution really 12.5% of the 100% OD?), were compared across all wavelengths to determine the most optimal wavelength. The most optimal wavelength would have the highest *R*^2^ values for all comparisons except OD to the consistency of the actual cell counts percent dilution at 12.5% dilution and below which would have the lowest *R*^2^ value. OD reads were done using the BioTek Synergy H1 hybrid spectrophotometer.

#### Nitrogen utilization

Nitrogen utilization tests were performed using 10 different nitrogen conditions in MN base media. 100 mM was the concentration used for all compounds that contained one nitrogen atom per molecule: proline, ammonium tartrate dibasic, serine, sodium nitrate, glycine, glutamate, and aspartate; 50 mM was the concentration used for urea because it has 2 atoms of nitrogen per molecule; 1% w/v of peptone was used as a positive control and no nitrogen was added as a condition for negative control (Table 3). This concentration of 100 mM was used because our standard growth media YPD contains 20 g/L of peptone, which contains 14% total nitrogen by weight. 14% total nitrogen per gram of peptone is 1.4 g of nitrogen per 100 mL of water, which if we use 1% peptone as a positive control that equals 100 mM total nitrogen in 1% of peptone. Liquid MN with MN salts (not 20× Nitrate salts) was used with the varying nitrogen sources to ensure that no alternative nitrogen source would be available to the fungi. Fungi were first grown up in liquid MEA for 5 days at room temperature to reach maximum density. Then, 1 mL of cells was removed and washed 3 times with water. 99 μL of each nitrogen source-containing medium was added to 6 wells in a 96-well plate, for 6 replicates, and 1 μL of the washed cells was added to each well. 100 μL of each medium was also added to one well each without cells to blank each condition because the different nitrogen sources created different colors of the medium. Daily growth was measured from day 0 to day 7 at 420 nm (the resulting optimal wavelength for OD reads), using the BioTek Synergy H1 hybrid spectrophotometer.

**Table 3.** Nitrogen sources, concentrations, and manufacturer.

Nitrogen source	Concentration	Catalog number
No nitrogen	N/A	N/A
Peptone	1% w/v	Fisher Brand: BP1420
L-proline	100 mM	Sigma: P-0380
Ammonium tartrate	100 mM	Sigma: A2956
L-serine	100 mM	Sigma: S4500
Sodium nitrate	100 mM	Fisher Brand: S343
Glycine	100 mM	Fisher BioReagents: BP381
L-glutamic acid	100 mM	Sigma: G1251
L-aspartic acid	100 mM	Sigma: A9256
Urea	50 mM	Alfa Aesar: A12360

#### Optimal growth temperature and range of growth temperatures

To determine the temperature resistance range and optimal growth temperature for each isolate, we grew both fungi at 4°C, 15°C, and 23°C (i.e. ambient room temperature), 28°C, 37°C, and 42°C. Isolates were first grown up in 25 mL of MEA for 5 days at room temperature to maximum density. Then 1 mL of cells was removed, and a 10× serial dilution was made from 0× to 100,000×, using prefilled 1.5 mL tubes with 900 µL of MEA and adding 100 µL of the previous tubes each time. Then 5 µL of each serial dilution was spotted onto a square MEA plate which allowed us to determine the carrying capacity of each isolate at the different temperatures. Plates were kept at their respective temperatures for 7 days before observations were made, however, the 37°C and 42°C plates were only kept at those temperatures for 2 days and the incubators required cups of water inside of them to prevent the plates from dehydrating. Plates grown in 42°C and 37°C were then allowed to grow at room temp for up to a week to determine if the isolates died at these temperatures or if their growth was just arrested.

#### UV resistance

Resistance to UV light was observed to determine if these black fungi, with highly melanized cell walls and constant exposure to sunlight in their natural habitat, were UV resistant. To determine this, we used the UVP HL-2000 HybriLinker UV crosslinker as our source of UV light, which has a UV wavelength of 254 nm. Lower wavelengths (100–280 nm) are in the UV-C range, they are considered ionizing radiation and are the most detrimental to living organisms, but are completely blocked by the ozone layer (Molina and Molina 1986; Schreier *et al.* 2015). Therefore, using this wavelength we are able to push our organisms beyond the UV limits found in their natural habitat and test extreme amounts of UV exposure.

The fungi were inoculated in 25 mL of MEA in a 250 mL Erlenmeyer flask and let grow under shaking conditions at 200 rpm for 5 days at room temperature to reach maximum density. 100 µL of this culture was then spread out onto 6 MEA plates, using a glass spreader. Three plates were kept as the control growth, to compare to the 3 other plates which were exposed to the UV light. Experimental plates were placed inside of the UV crosslinker with their lids taken off. Then the plates were exposed to 120 seconds of UV light from a distance of 9.5 cm to the light source at 10,000 μJ/cm^2^ (254 nm) (Frases *et al.* 2007). We then wrapped all plates in aluminum foil and placed them in the Percival light incubator set at 23°C for 2 days. Placing UV-exposed cells in complete darkness after exposure is essential for limiting repair via photoreactivation (Weber 2005). After 2 days the plates were removed from the aluminum foil and left in the incubator for 5 more days before final observations were made. To determine whether a particular isolate was resistant to UV exposure, the growth of the fungi exposed to UV was compared to the controlled growth in imageJ, by comparing the pixel areas of the plates.

#### Metal resistance

Metal resistance is a relatively universal trait in many polyextremotolerant fungi. Due to the under-studied nature of this particular characteristic in BSCs and fungi, we decided to test if any of our isolates were resistant to any heavy metals which would indicate possible bioremediation capacity. In order to test metal resistance, we used the antibiotic disk method by aliquoting metal solutions onto paper discs and observing zones of clearance. The metals and concentrations used are listed in Table 4. For testing, 5 µL of each metal solution was aliquoted onto a dry autoclaved Whatman filter paper disc which was created using a standard paper hole puncher. These discs were then allowed to air dry and kept at 4°C for up to a week. Initial growth of the fungi (*E. viscosa* JF 03-3F, *E. viscosa* JF 03-4F, *E. dermatitidis*, and *S. cerevisiae* ML440) was done in 25 mL of MEA, shaking at 200 rpm for 5 days at room temperature. We then spread 100 μL of each fungus onto 100 mm-sized MEA plates using a glass spreader to create a lawn. Using flame sterilized tongs our metal paper discs were placed onto the center of the petri dish on top of the fungal lawn and lightly pressed down to ensure the metal disc was touching the plate completely. These plates were then placed in the Percival light incubator at 23°C with a 12 hr light/dark cycle for up to 2 weeks. Once a zone of the clearing was clearly visible amongst the fungal growth (1–2 weeks), the zone of clearing was then measured in cm. Generally, large zones of clearing indicated sensitivity to the metal, whereas zones of reduced size were consistent with resistance to the metal.

**Table 4.** Metals used, their concentration, and manufacturer.

Metal	Concentration	Catalog number
Fe(III)SO_4_	0.6 M	Fisher: I146
CoCl_2_	0.5 M	Sigma: C-2644
NiCl_2_	1.5 M	Sigma-aldrich: 223387
CuCl_2_	1.5 M	Sigma: 203149
CdCl_2_	10 mM	Fisher: 7790-84-3
AgNO_3_	0.47 M	Alfa Aesar: 7761-88-8

#### Lipid profiles

Comparison of the lipid production of *S. cerevisiae*, *E. dermatitidis*, *E. viscosa* JF 03-3F, and *E. viscosa* JF 03-4F was performed in the presence of fermentable vs nonfermentable sugars in high and low nitrogen. To test these conditions, we grew all 4 fungi in 4 different media types: (1) MEA; (2) MEA + 20 g/L of peptone instead of 2 g/L; (3) MEA with the dextrose replaced with the same weight amount of glycerol; and (4) MEA with glycerol instead of dextrose and 20 g/L of peptone instead of 2 g/L. All 4 fungi were first inoculated in 25 mL of liquid MEA in a 250 mL Erlenmeyer flask and shaken at 200 rpm for 5 days at room temperature to reach peak density. Then 100 μL was inoculated into 5 mL of each media in a size 25 mm tube, placed in a roller drum, and allowed to grow at room temperature for 5 days.

To observe their lipid profile, we performed a standard Bligh Dyer lipid extraction (Bligh and Dyer 1959). Equal wet weight of each organisms’ cells was pelleted and re-suspended in 2 mL of methanol inside 16 mm glass tubes. Tube openings were covered in Duraseal before applying the lid of the tube, then samples were boiled for 5 minutes and let cool for 10 minutes. Then 2 mL of chloroform and 1.6 mL of 0.9% NaCl were added, and the tubes were vortexed to fully mix. Tubes were then centrifuged at 5000 rpm for 5 minutes to separate the layers. The bottom lipid layer of the suspension was removed and placed in a new glass tube which was then dehydrated using nitrogen gas till the samples became fully dried. Dehydrated samples were then re-suspended with 100 μL of a 9:1 ratio of chloroform: methanol to run the thin layer chromatography (TLC).

For all samples except the *S. cerevisiae*, 7 μL of the lipid suspension was used to dot the TLC. For *S. cerevisiae,* 10 μL of the lipid suspension was needed. The solvent systems used for TLC were chloroform: methanol: glacial acetic acid: water 85:12.5:12.5:3 for the polar lipid solvent system, and petroleum ether: diethyl ether: acetic acid 80:20:1 for the neutral lipid solvent system. The TLC plates were loaded with 7 or 10 μL of the re-suspended samples, and they were placed in the polar solvent system for approximately 30 minutes (half-way up the plate) before transferring to the neutral lipid solvent system in a separate container till the solvent front reached just a few cm below the top of the plate. The plate was then removed and dried for 15 minutes, until the solution on the plate was no longer volatile by smell, and the plate was placed in the presence of iodine (Sigma-Aldrich cat. No. 207772) in a glass chamber for 5 minutes until all the lipids were visible. The plates were then immediately placed in plastic covers and scanned and photographed for visualization and documentation.

### Melanin biosynthesis and regulation experiments

#### Melanin biosynthesis gene annotation

Melanin biosynthesis in fungi occurs via 3 different pathways: the 1,8-DHN pathway which creates allomelanin, the L-DOPA pathway which creates pheomelanin, and the tyrosine degradation (HGA) pathway which creates pyomelanin (Gessler *et al.* 2014; Cao *et al.* 2021) (Fig. 1). Most fungal species only contain one melanin biosynthetic pathway, but there are many species in Pezizomycotina, particularly in the genera *Aspergillus* and *Exophiala*, which are capable of producing 2 or all 3 forms of melanin (Teixeira *et al.* 2017; Perez-Cuesta *et al.* 2020). For that reason, we decided to manually annotate the *E. viscosa* JF 03-3F and *E. viscosa* JF 03-4F genome sequences to determine if they too possessed multiple melanin biosynthetic pathways. In all cases, the relevant *A. niger* and *A. fumigatus* proteins were used as queries (Chen *et al.* 2014; Teixeira *et al.* 2017). Protein sequences for each gene were found using the *Aspergillus* genome database (AspGD) (now called FungiDB https://fungidb.org/) and were tested using protein-protein BLAST against the filtered model proteins database of *E. viscosa* JF 03-3F *and E. viscosa* JF 03-4F on Mycocosm. Since *A. niger* contains paralogs for some melanin biosynthetic genes, all genes listed in Teixeira *et al.* (2017), Chen *et al.* (2014) were used as queries for BLAST searches. Additionally, the protein sequences for scytalone dehydratase (Arp1) and 1,3,8-trihydroxynapthelene reductase (Arp2) are not homologous between *A. niger* and *A. fumigatus* (Jørgensen *et al.* 2011), therefore, both species’ protein sequences were used for BLASTing against *E. viscosa* JF 03-3F and *E. viscosa* JF 03-4F. Once the melanin biosynthetic genes in *E. viscosa* JF 03-3F and *E. viscosa* JF 03-4F were identified, their highest matching protein sequences were then reverse BLASTed to the *A. niger* genome to determine the reciprocal best hit to ensure true homology. Similarity scores (>40%), e-values (<10^−6^), and bit scores (>50) of the protein-protein BLASTs followed standards for homology laid out by Pearson (2013).

#### Regulation of melanin production using chemical blockers

Once it was established that both isolates contain the potential for the production of all 3 fungal melanins, we wanted to determine which melanin was actively being produced using chemical blockers of the 1,8-DHN and L-DOPA-melanin production pathways. DHN-melanin blocker phthalide and the L-DOPA-melanin blocker kojic acid were both used to determine which melanin was being actively produced. Stock solutions were made according to (Pal *et al.* 2014): phthalide was diluted in 70% ethanol, and kojic acid in DMSO. Three separate experiments were performed using these melanin blockers, to determine which method would provide the most informative results.

The first was the disc diffusion method whereby Whatman filter paper discs were autoclaved and impregnated with 5 μL of either 10 mM of phthalide or 10 mg/mL of kojic acid. Impregnated filter paper discs were then placed on top of freshly spread lawns of either isolate on both MEA and YPD. Lawns were of 50:50 diluted 5-day-old cells grown in MEA, and 100 µL of this dilution was spread onto the petri plates with a glass spreader. These plates were then grown at 23°C with 12 hr light/dark cycles for 5 days. Additionally, both a kojic acid disc and phthalide discs were placed on fungal lawns ∼4 cm apart simultaneously to observe their specific melanin-blocking capabilities on the same cells.

Next, we tried adding the melanin blockers directly to the medium as was done in Pal *et al.* (2014). Since melanin is more universally distributed in *Exophiala* cells compared to *Aspergillus* cells, we decided to use the highest concentration of both kojic acid and phthalide that was used by Pal *et al.* (2014), which was 100 mM of each compound. This concentration of each compound was added to both solid YPD and MEA after autoclaving, chemicals were added both individually and combined. These plates were then used for 2 forms of growth experiments, serial dilutions, and lawn growth. We spread a lawn of either fungus onto YPD and MEA with and without kojic acid, phthalide, and both compounds at 100 mM each. And we performed a 10× serial dilution of both *E. viscosa* JF 03-3F and *E. viscosa* JF 03-4F up to 10,000× diluted and spotted 5 μL of each dilution onto MEA plates with and without Kojic acid, Phthalide, and both compounds. We let both growth experiments grow at 23°C for 5 days with a 12 hr light/dark cycle.

#### Evaluating the cause of melanin excretion

To assess the role of organic nitrogen levels in melanin excretion, we initially switched the concentration of peptone added to YPD and MEA media such that the new media were YPD + 0.2% peptone and MEA + 2% peptone. *E. viscosa* JF 03-3F and *E. viscosa* JF 03-4F were grown in liquid MEA for 5 days with shaking at room temperature and were plated onto these new media using the same technique as described above for growth comparison on different media. To determine if a more gradual increase in peptone would correlate with a gradual excretion of melanin, we took the base media of MEA (solid) and changed the concentration of peptone to 0.1%, 0.5%, 1%, 1.5%, 2%, 2.5%, 3%, 3.5%, 4%, and 5%. We then spotted 5 μL of both strains onto the plates after performing a 10× serial dilution up to 10,000× dilution. The plates were grown at 23°C for 10 days with a 12 hr light/dark cycle.

Additionally, we tested if tyrosine levels (a direct precursor for L-DOPA and HGA-derived melanin) were the source of the increased melanin excretion in the increased peptone experiment. Growth of 5 μL spots of *E. viscosa* JF 03-3F and *E. viscosa* JF 03-4F were grown on “Tyrosine media” (Jalmi *et al.* 2012) (2% w/v dextrose, 1% w/v peptone, 0.1% w/v yeast extract (Table 1)): without added tyrosine, with 0.5 g/L tyrosine, with 1 g/L tyrosine, and MN-nitrate 2% w/v dextrose with 0.5 g/L tyrosine as the sole nitrogen source. Colonies were allowed to grow for 19 days and then observations were made to see if melanin excretion was induced by different tyrosine levels.

#### Melanin extraction and spectrophotometric measurements

Extraction of melanin from a variety of sources has been performed using 2 different approaches: chemical extraction and enzymatic extraction (Pralea *et al.* 2019). We performed both chemical and enzymatic extractions on our fungi's melanin. The enzymatic extraction method that was used came from Rosas *et al.* (2000), and the chemical extraction method, which has been used more extensively in previous works, was derived from Pal *et al.* (2014). Pal *et al.*'s method for extraction and purification of melanin from culture filtrate was adapted and used for all future melanin extractions, mainly supernatant melanin extractions. Adjustments to the Pal *et al.* method included: the 6 M HCl precipitation taking multiple days instead of overnight for melanin to precipitate, and the protocol was stopped when 2 M NaOH was added to the extracted melanin. We did not continue to re-precipitation and drying of the melanin as this product did not reprecipitate in any solvents used.

The exact methods are as follows. 10 mL of culture was centrifuged at 3,000× g for 5 minutes, and the resulting supernatant was filter sterilized through a 2 μm filter to ensure all cells were removed. The filtered supernatant was then transferred into a 50 mL centrifuge tube, and 40 mL of 6 M HCl was added to the tube. The filtrate was then allowed to precipitate out for up to 2 weeks. Precipitated solutions were then centrifuged at 4,000× g for 3 minutes, and the resulting supernatant was discarded. The pellet was washed with 2 mL of dd H_2_O, vortexed, centrifuged, and the supernatant discarded. Then 3 mL of 1:1:1 chloroform: ethyl acetate: ethanol was added to the samples and vortexed vigorously to ensure re-distribution of the melanin. The tubes were then centrifuged at 4,000× g for 1 minute, and any resulting clear layers (top and or bottom) were discarded, leaving behind the dark layer. 2 mL of water was added to the sample for washing, and the tubes were centrifuged at 4,000× g for 1 minute, and the entire supernatant was discarded. Finally, 1 mL of 2 M NaOH was added to each sample to allow for a standard volume added even if the melanin amount and, therefore, the final volume varied.

Extracted melanin samples suspended in 1 mL of 2 M NaOH were then diluted 5 μL into 195 μL of 2 M NaOH into a 96-well plate, with a 200 μL 2 M NaOH blank well. These diluted samples were then read using the BioTek Synergy H1 hybrid spectrophotometer. The settings were for a full spectrum read from 230 to 700 nm, with 10 nm steps. However, the machine could not read OD values above 4.0, and therefore, only data from 300 to 700 nm was used.

#### Confirming active melanin excretion from living cells and determining its OD amount in the supernatant

To confirm that *E. viscosa* JF 03-3F and *E. viscosa* JF 03-4F were actively excreting melanin, as opposed to dead cells lysing and releasing it, we grew up both strains and took daily aliquots for melanin extraction. We use the term excrete/excreting instead of secrete/secreting for the terminology of melanin being released into the media by the fungi because we have no evidence currently that this melanin release mechanism is an active process (i.e. via Golgi, efflux pumps, or autophagy) warranting active secretion. Additionally, we wanted to compare the melanin excretion capabilities of these strains to *E. dermatitidis* for a baseline comparison. All 3 fungi were grown in liquid MEA shaking at room temperature for 5 days. Then 2 mL of cells were washed with water 3 times. 500 μL of washed cells were then inoculated into 100 mL of MEA and YPD in 500 mL flasks. We let the cells grow at 23°C shaking at 200 rpm for 7 days, removing 11 mL of cells and supernatant daily and pipetting them into 15 mL centrifuge tubes. The tubes of cells were then centrifuged at 3000× g for 5 minutes, the supernatant was removed, filter sterilized through a 2 μm filter, and placed into a new tube. We filter sterilized the supernatant to ensure that no cells remained in the supernatant, therefore, all of the melanin extracted came only from excreted melanin. Melanin was then extracted using the chemical method explained above. The resulting pure melanin for all samples was read with the full spectrum as stated above, and both standard OD and log scale graphs were created to confirm the presence of melanin with the proper *R*^2^ value above 0.9 (Pralea *et al.* 2019).

### Albino mutant creation for genetic analysis of melanin production

#### Creation of ethyl methanesulfonate mutants and annotation of their mutations

Genetic modification of *E. viscosa* has not been established yet. Therefore, random mutagenesis was performed using ethyl methanesulfonate (EMS) to induce G:C to A:T mutations EMS mutagenesis was performed using the method by Winston (2008). Albino mutants and other interesting pigmentation or morphological mutants were isolated from the resulting mutagenesis, and their DNA was extracted using the CTAB DNA extraction protocol described above. DNA was then sent to the former Microbial Genome Sequencing Center (MiGS; https://www.migscenter.com/; 355 Fifth Avenue, Suite 810 Pittsburgh, PA 15222, now called SeqCenter (https://www.seqcenter.com/; 91 43rd Street, Ste. 250 Pittsburgh, PA 15201) for genome re-sequencing and base-called against the WT genome sequences. Resulting mutations were then manually annotated using the JGI Mycocosm genome “search” tool to identify affected genes.

#### Recovery of melanin production in albino mutants: modern use of the classical beadle and tatum biochemical-genetics experiment

Following the recovery of an albino mutant, we attempted to restore melanin production via chemical induction of the other melanin biosynthetic pathways. We did this using hydroxyurea (HU), L-DOPA, 1,8-DHN, and tyrosine. HU has been shown to enhance melanin production in *E. dermatitidis*, and L-DOPA is needed for certain fungi to produce melanized structures, including the albino mutant form of *E. dermatitidis* WdPKS1 (Paolo *et al.* 2006; Dadachova *et al.* 2007; Schultzhaus *et al.* 2020). Both YPD and MEA medium was made up and 20 mM of HU, 1 mM of L-DOPA, or 1 mM of 1,8-DHN was added to the medium after autoclaving. 5 μL of albino and WT control cells were spotted onto these media with added compounds, and they were grown for 10 days at 23°C 12 hr light/dark cycle.

Additionally, we also tested the presence of tyrosine, the starting compound for L-DOPA and HGA melanins on the albino mutant. Growth of 5 μL spots of EMS 2-11 were grown on “tyrosine media” (Jalmi *et al.* 2012) (2% w/v dextrose, 1% w/v peptone, 0.1% w/v yeast extract (Table 1)): without added tyrosine, with 0.5 g/L tyrosine, with 1 g/L tyrosine, and MN-nitrate 2% w/v dextrose with 0.5 g/L tyrosine as the sole nitrogen source. Colonies were allowed to grow for 19 days and then observations were made to see if melanization was recovered by different tyrosine levels.

## Results

### Fungal isolation, ITS alignment, and general morphological observations

Two fungi were isolated from BSCs located near Jackman Flats Provincial Park in BC, Canada (latitude: 52.94997°, longitude: −119.41919°, and altitude: 738). BSC samples were diluted in water, crushed with a plastic micropestle, and plated out onto MEA with antibiotics and cycloheximide, and grown at 23°C for 2 weeks to obtain the isolates. Once colonies began to form, they were picked and struck out onto regular MEA to obtain pure cultures. DNA extractions were performed on liquid cultures of the isolates that had been grown in liquid MEA for 5 days at 23°C and 160 rpm of shaking. PCR amplification of the isolates’ ITS region was performed and then sequenced using Sanger sequencing. The resulting ITS sequences placed the fungal isolates within the genus *Exophiala*, and later apparent sequencing errors created nucleotide differences in their ITS sequences which initially placed them as different species. Further whole genome sequencing using Illumina-based sequencing allowed for more accurate sequences of the ITS region for these isolates.

Using the Illumina-based ITS sequences of the isolates, it was determined that they have 100% identity to each other's ITS sequence (Supplementary File 1). BLAST results to their ITS sequences using the UNITE simple analysis (Nilsson *et al.* 2019) resulted in a 97.57% identity to “*Exophiala nigra* strain CBS 535.95” accession number: MH862481.1, which was performed on September 29th, 2022. UNITE's fungal species identification delineation typically separates species on ITS sequence similarity with ≥98% as the cutoff (Kõljalg *et al.* 2013), indicating that via ITS sequence alone, our *Exophiala* isolates (introduced here as *E. viscosa* JF 03-3F and JF 03-4F) were a separate species from *E. nigra*. However, since that date, the UNITE database has been further curated with more strict parameters and now only contains 19 *Exophiala* species, removing *E. nigra* and *E. sideris* as species in Taxon name searches. Repeating this analysis on December 12th, 2022, resulted in a BLAST result of *Capronia acutiseta* UDB035414 86.16% identity as the highest match.

Although both *E. viscosa* JF 03-3F and *E. viscosa* JF 03-4F are 2 strains of the same species indicated via their marker gene alignment, their cellular morphology and budding patterns differ drastically as described below. Both strains form smooth, shiny, sooty black colonies on PDA after 9 days of growth at 23°C. When grown on high organic nitrogen media such as YPD, they will begin to excrete melanin into the agar by day 7 when grown at 23°C, and by day 3 in liquid YPD (Figs. 4l, 5l, and 14). On MEA, they are grayish brown in color, have the consistency of sticky black tar (Supplementary Videos 1 & 2), and have an iridescent rainbow shine which forms on their colonies after a week at 23°C (Figs. 4k and 5k & Supplementary 3). These fungi also fail to easily disperse when suspended in water but can be readily pelleted. These observations and the prominence of lipid bodies within the cells (Supplementary Fig. 4) suggested that lipid-derived compounds could cause their sticky, water-repelling, and iridescent nature. These isolates grow to their maximum density in 7 days at 23°C in 25 mL of MEA in a 250 mL Erlenmeyer flask shaken at 160 rpm. Both *E. viscosa* JF 03-3F and *E. viscosa* JF 03-4F have been preserved in a metabolically inactive state at the Westerdijk Institute.

### Phylogenetic placement of *E. viscosa* amongst the genus *Exophiala*

Phylogenetic analyses were performed using the marker genes 18S rDNA (1,600 bp), 28S rDNA (548 bp), and ITS rDNA (547 bp), with a combined sequence length of 2,685 base pairs. These genes were chosen because while they are predominant marker genes, they are also the only genes available on NCBI for the species *Phaeoannellomyces elegans*, which has been identified as closely related to this clade regardless of its nomenclature (Moussa *et al.* 2017). Bayesian analysis of the multi-locus gene tree yielded the best scoring tree, with the final log-likelihood value of −9,031.1. Estimated base frequencies were: A = 0.242, C = 0.23, G = 0.275, and T = 0.253; with substitution rates of: AC = 0.161, AG = 0.151, AT = 0.143, CG = 0.067, CT = 0.412, and GT = 0.065. The shape parameter of the gamma distribution of rate variation alpha was 0.141, and the total tree length was 0.574. The final average standard deviation of split frequencies at the end of the total 500,000 MCMC generations was 0.002955.

**Fig. 2.**
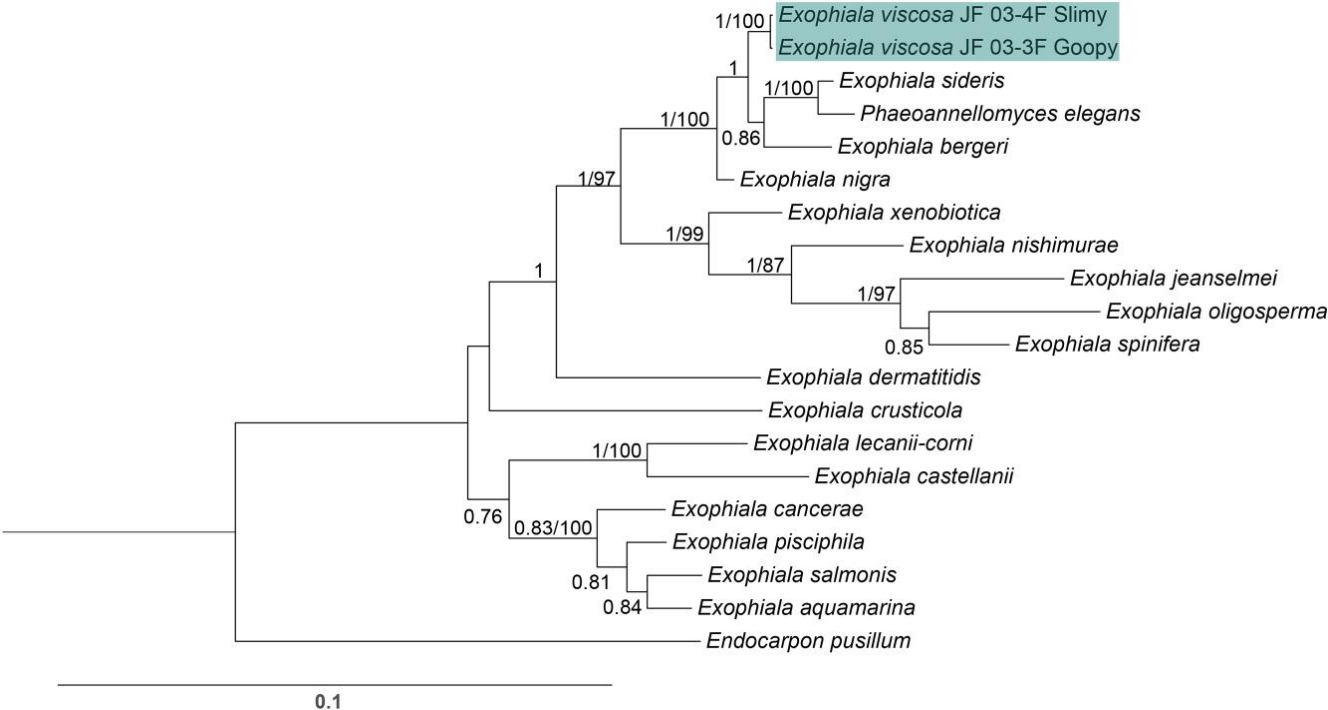
Rooted Bayesian phylogenetic tree of the concatenation of the 18S rDNA, 28S rDNA, and ITS rDNA regions of available *Exophiala* species with the addition of *E. viscosa* JF 03-3F and *E. viscosa* JF 03-4F to identify the phylogenetic location of these new species within the genus *Exophiala*. The tree with the highest log-likelihood is shown. The numbers above the branches are the Bayesian posterior probability/maximum likelihood bootstrap values, with posterior probabilities >75% and bootstrap values >80 shown. Location of *E. viscosa* JF 03-3F and *E. viscosa* JF 03-4F within *Exophiala* places them closest to *E. bergeri*, *P. elegans*, and *E. sideris*.

The multi-locus combined Bayesian inference and Maximum likelihood tree placed *E. viscosa* within the monophyletic clade composed of *E. nigra*, *E. sideris*, *E. bergeri*, and *P. elegans* with a posterior probability of 100% (Fig. 2). In this combined Bayesian and Maximum likelihood tree, *P. elegans* is placed with 100% posterior probability and a bootstrap value of 100 as related to *E. sideris* and distinct from *E. nigra*. Additionally, *E. nigra* is further separated from *E. viscosa*, placing *E. nigra* on its own node beneath *E. bergeri* with 100% posterior probability and 100 bootstrap value (Fig. 2). Both strains of the new fungus *E. viscosa* form a monophyletic node separate from *E. nigra*, *E. sideris*, *E. bergeri,* and *P. elegans* with a posterior probability of 100%, providing supporting that these strains are a novel species.

**Fig. 3.**
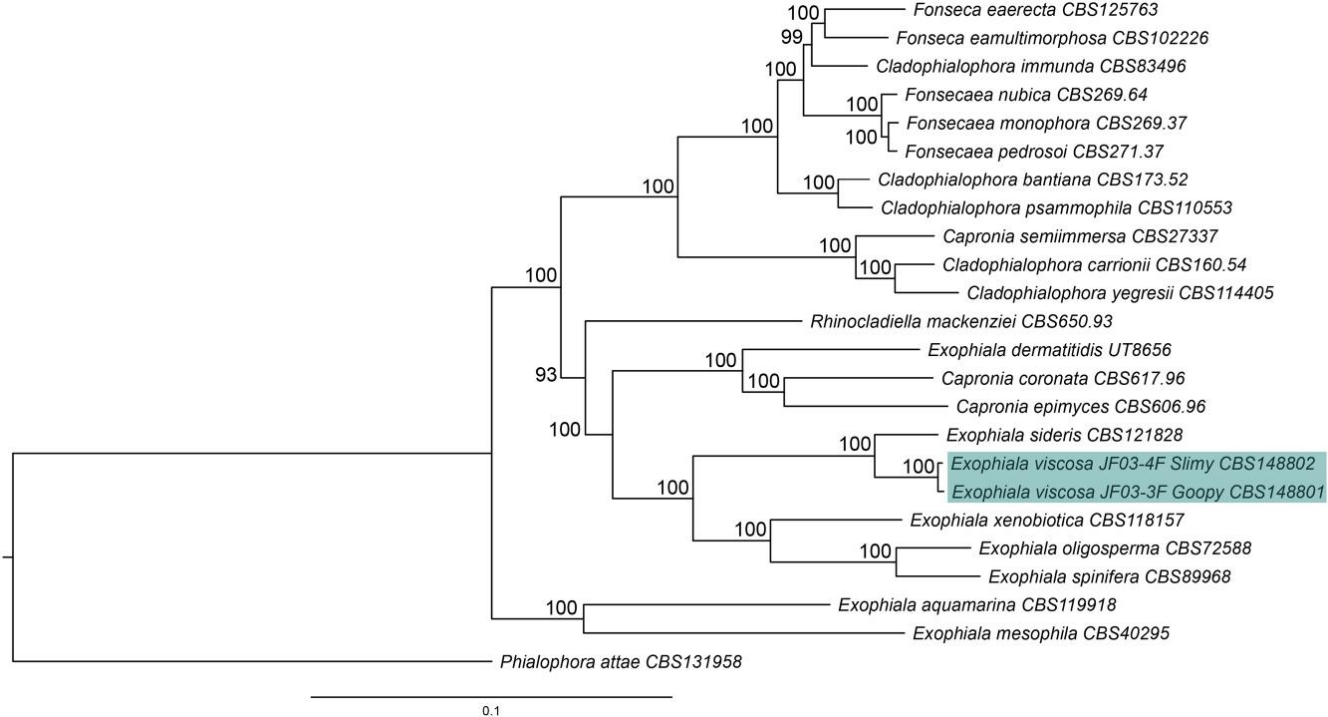
Single-copy homologous protein sequence approximate maximum likelihood phylogenetic tree of taxa of the family herpotrichielleaceae all of which have been whole genome sequenced and annotated. *E. viscosa* JF 03-3F and *E. viscosa* JF 03-4F are shown to be most closely related to *E. sideris*. Node values represent bootstrap values.

One additional marker gene sequence is available for *E. nigra* which is not available for *P. elegans*, and that is *RPB1*. When *RPB1* (464 bp) is added to the gene concatenation, creating the combination of 18S, 28S, ITS, and *RPB1* (3,156 bp total), *E. nigra* is again placed on its own node below *E. bergeri* maintaining a separate position to *E. viscosa* and the *E. sideris* clade with 100% posterior probability and 79 bootstrap value (Supplementary Fig. 2). This tree also shows *E. sideris* as the closest relative to *E. viscosa* with 100% posterior probability and a bootstrap value of 100. Within the 3 gene concatenations (18S, 28S, and ITS), the percent identical base pairs between *E. viscosa* and *E. sideris* are 98.436%, and 99.07% identical bases between *E. viscosa* and *E. nigra*. However, when *RPB1* is added to the concatenation, the percent identical bases between *E. sideris* and *E. viscosa* remain similar at 98.551%; whereas the percent identical bases between *E. viscosa* and *E. nigra* becomes 97.03%. Percent identity matches between all species represented in the phylogenetic trees of Fig. 2 and Supplementary Fig. 2 across all genes and gene concatenations are provided in Supplementary File 1. Further phylogenetic analyses were performed on all *Exophiala* species and related fungi who have been whole genome sequenced, and whose genomes are available in JGI's Mycocosm. With their whole genomes, we were able to perform single-copy homologous protein sequence approximate maximum likelihood phylogenetic analyses, providing additional support for *E. viscosa*'s intraspecific variation from specifically the fungus *E. sideris*. Our approximate maximum likelihood single-copy homologous protein phylogenetic tree shows with a 100 bootstrap value, that *E. sideris* is separate from *E. viscosa* (Fig. 3).

ASAP values of the marker genes allowed us to confirm our phylogenetic inferences of separating *E. viscosa* from the other species in its clade. For the ITS-only ASAP analysis, the output provided an ASAP score of 1.0–18 distinct species with a distance threshold of 1.32% (Supplementary File 2). Both strains of *E. viscosa* were considered one species, and *E. sideris* and *P. elegans* were considered one species. When ASAP was performed on the concatenated 18S-28S-ITS alignment, it maintained that there should be 18 distinct species (ASAP score of 2.0), with both strains of *E. viscosa* as one species and *E. sideris* and *P. elegans* as one species. However, with the three-gene concatenation, the distance threshold decreased to 0.75%. Finally, the concatenation of 18S-28S-ITS-*RPB1* provided an ASAP score of 2.5 which suggested that there be 16 species with a distance threshold of 2.5%, combining both strains of *E. viscosa* into one species, and combining *E. aquamarina* and *E. pisciphila* into one species (*E. crusticola* and *P. elegans* are not in this dataset). All 3 ASAP analyses suggest that both strains of *E. viscosa* remain as one species and that they are a distinct species from the other *Exophiala* spp.

### Morphological differences between *E. viscosa* strains JF 03-3F and JF 03-4F

**Fig. 4.**
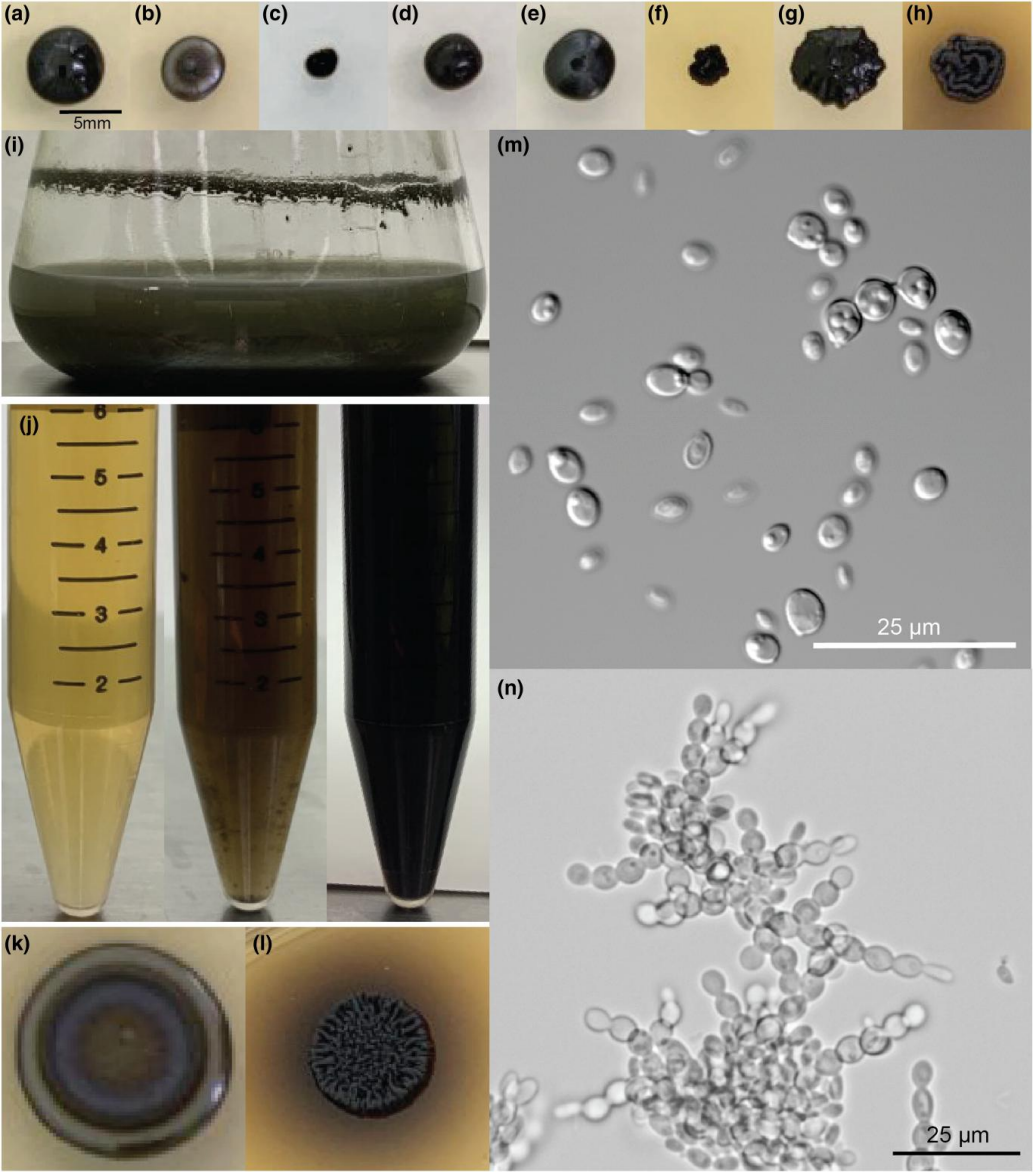
*E. viscosa* JF 03-3F's description summary figure. All colonies: day 14 grown at 23°C) a) Colony on MAG; b) Colony on MEA; c) Colony on MN-NAG; d) Colony on MNV; e) Colony on PDA; f) Colony on Spider media; g) Colony on V8; h) Colony on YPD; i) Biofilm at air-liquid Interface of growth in MEA day 3; j) Supernatant when grown in YPD; Day 0, 3, 6; k) Colony growth from 5 µL of liquid culture on MEA after 14 days with rainbow sheen; l) Colony growth from 5 µL of liquid culture on YPD after 14 days with dark excretions; m) Cellular growth in liquid MEA, shaking at room temperature day 5; n) Cellular growth on solid MEA, room temperature hour 65, using VALAP method.

**Fig. 5.**
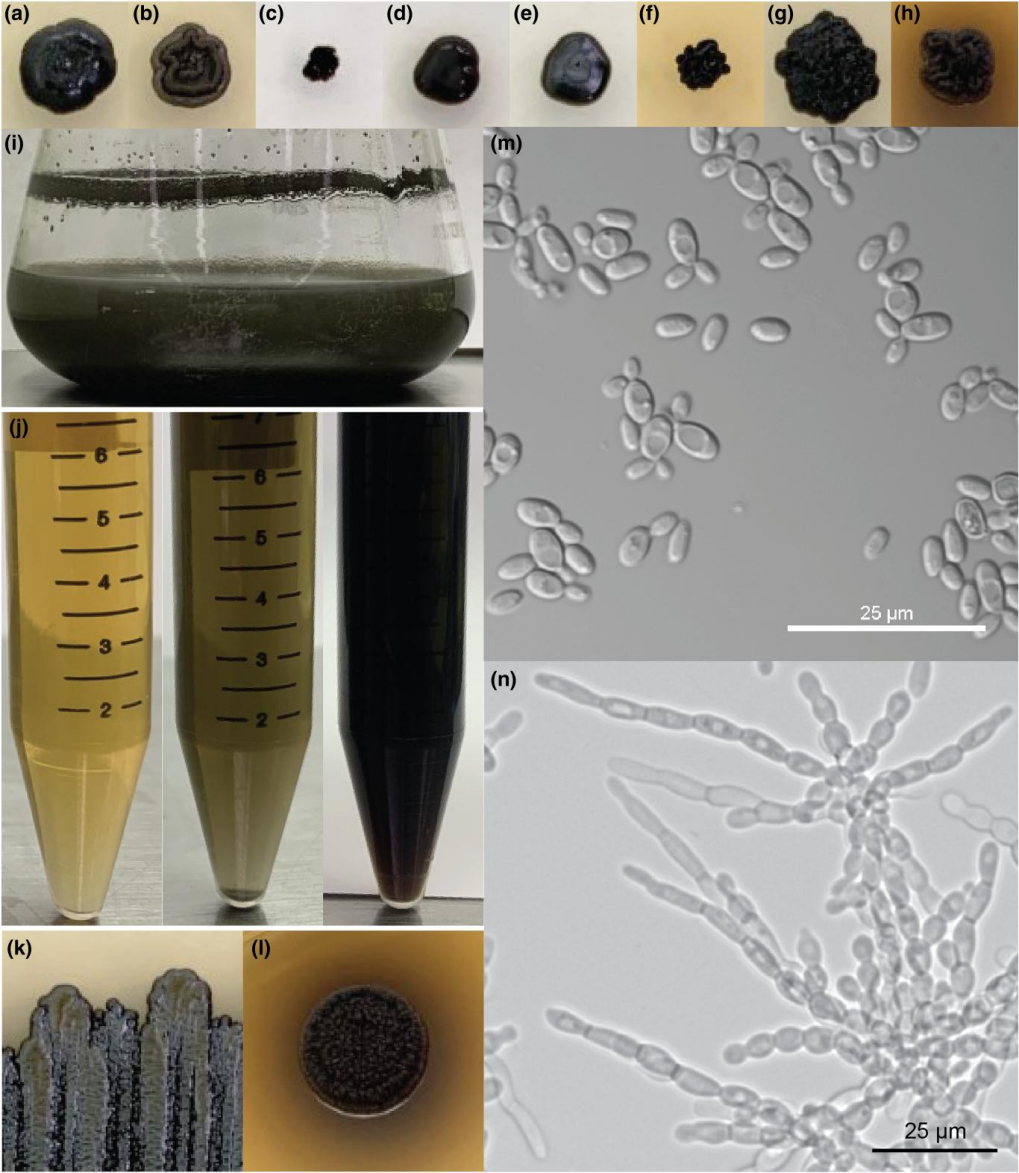
*E. viscosa* JF 03-4F's description summary figure. All colonies: day 14 grown at 23°C a) Colony on MAG; b) Colony on MEA; c) Colony on MN-NAG; d) Colony on MNV; e) Colony on PDA; f) Colony on Spider; g) Colony on V8; h) Colony on YPD; i) Biofilm at air-liquid interface of growth in MEA day 3; j) Supernatant when grown in YPD; Day 0, 3, 6; k) Colony growth from 5 µL of liquid culture on MEA after 14 days with rainbow sheen; l) Colony growth from 5 µL of liquid culture on YPD after 14 days with dark excretions; m) Cellular growth in liquid MEA, shaking at room temperature day 5; n) Cellular growth on solid MEA, room temperature hour 60, using VALAP method.

**Fig. 6.**
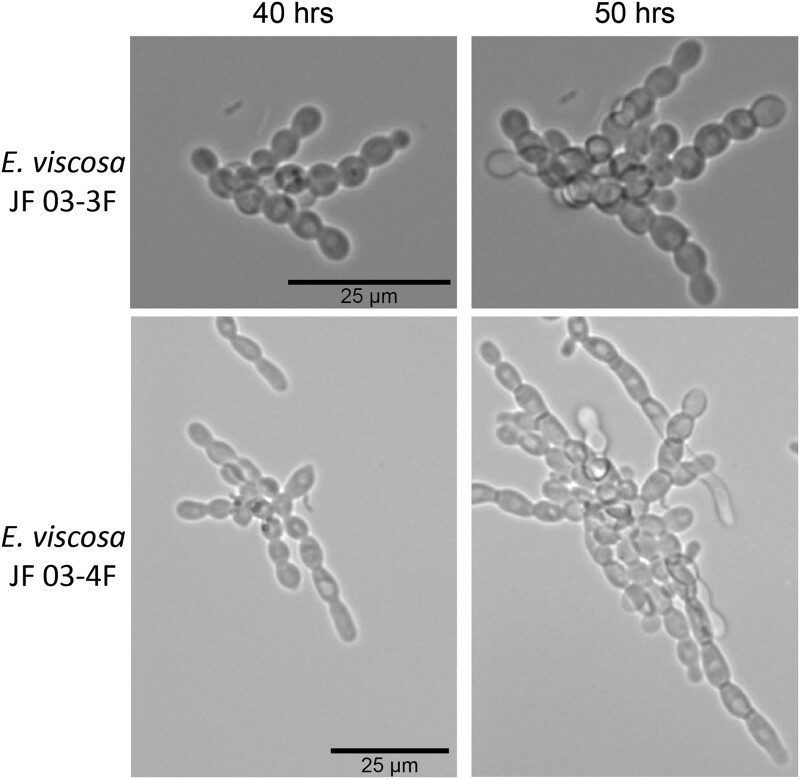
Budding styles of *E. viscosa* JF 03-3F and *E. viscosa* JF 03-4F pseudohyphae. Rate of budding is higher in *E. viscosa* JF 03-4F than in *E. viscosa* JF 03-3F, as seen at the 50 hr mark. Budding style is also different between the species, *E. viscosa* JF 03-3F buds both distal polarly and proximal at close to a 90° angle, whereas *E. viscosa* JF 03-4F buds almost exclusively as a distal polar. *E. viscosa* JF 03-4F's cells also elongate with every bud, forming almost hyphal-like structures at the 50 hr timepoint (scale bars apply to side-by-side images).

While these 2 strains of *E. viscosa*, JF 03-3F and JF 03-4F, are very similar to each other in genomic content and have 100% similarity across the 4 marker genes 18S, 28S, ITS, and *RPB1*, they vary in gene synteny, they contain multiple single nucleotide polymorphisms across their genomes, and their morphologies differ. *E. viscosa* JF 03-3F is found primarily as a yeast morphology on all media tested, with ellipsoidal cells 4.4 μm ± 0.34 μm wide and 5.36 μm ± 0.37 μm long (*n* = 20 cells measured; in MEA). *E. viscosa* JF 03-4F is found primarily in a pseudohyphal form on all media tested, with more elongated cells 3.6 μm ± 0.38 μm wide and 6.0 μm ± 0.67 μm long (*n* = 20 cells measured; in MEA). *E. viscosa* JF 03-3 will form pseudohyphae when grown for longer than 2 weeks and only on solid media, whereas *E. viscosa* JF 03-4F readily form pseudohyphae in liquid media and on solid media as a primary growth from and will form a few true hyphae after 3 weeks of growth on MEA (Figs. 5n and 6, & Supplementary Video 3). As seen in Fig. 6 and Supplementary Fig. 3d, the cellular morphology of *E. viscosa* JF 03-4F resembles that of *Hortaea werneckii* as observed by Mitchison-Field *et al.* (2019). Budding of *E. viscosa* JF 03-3F cells occurs at 180° or 90° from the mother cell's origin, whereas JF 03-4F is almost exclusively budding 180° from the mother cell. When *E. viscosa* JF 03-4F is grown up to maximum density in liquid culture, pipetting becomes difficult due to large clumps of cells formed by its more filamentous growth pattern (Figs. 5n and 6). However, *E. viscosa* JF 03-3F at maximum density in liquid culture does not form large clumps and, therefore, creates no pipetting issues. Notably, *E. viscosa* JF 03-4F possesses a looser pellet and is less refractory to re-suspension than *E. viscosa* JF 03-3F. *E. viscosa* JF 03-4F also produces a fuller, thicker biofilm at the air-liquid interface than *E. viscosa* JF 03-3F, as seen in Figs. 4i and 5i. *E. viscosa* JF 03-3F is also a much more prolific melanin excreter than *E. viscosa* JF 03-4F (Figs. 14–16).

### Recognition of novel taxon *E. viscosa*

The new species *E. viscosa* is defined based on molecular phylogenetic analyses of the 4 marker regions: 18S, 28S, ITS, and *RPB1* resulting in the holotype and the additional strain forming one monophyletic node separate from *E. sideris, P. elegans, E. bergeri,* and *E. nigra* which are its closest relatives, with the genes ITS and *RPB1* providing the most support for intergenic variation (Fig. 2 and Supplementary Fig. 2). Additional phylogenetic support results from the single-copy gene tree of whole genome sequenced *Exophiala* spp. and related fungi, which further supports intraspecific variation of *E. viscosa* from *E. sideris* (Fig. 3). Whole genome sequencing and then single-copy gene phylogenetic analyses of *E. nigra* and *P. elegans* would have been beneficial for further elucidation of this fungus, but that was not feasible at this time. Additionally, ASAP values of all 3 gene/gene concatenations tested were in consensus that *E. viscosa* is a separate species from *E. sideris*, *E. bergeri*, *P. elegans*, and *E. nigra*.

Morphological comparison of *E. viscosa* to *E. nigra* from images in Moussa *et al.* (2017) show differences in colony and cell morphology. Colonies of *E. nigra* on MEA showed no rainbow sheen and remained olivaceous black, with irregular undulate colony edges (Moussa *et al.* 2017). Whereas *E. viscosa*'s colonies are smooth to curled, is dark grayish brown in color, and have a rainbow sheen. *E. nigra* colonies on PDA were also irregular and undulate on the edges and umbonate or lumpy on the elevation morphology (Moussa *et al.* 2017), whereas *E. viscosa* has smooth and circular edges with a flat elevation on PDA (Figs. 4e and 5e). Cellular morphology images of *E. nigra* on MEA showed pseudohyphae to hyphal formation with conidia forming on the tips of hyphae (Moussa *et al.* 2017). Pseudohyphae are observed in *E. viscosa* on MEA, but hyphae are rarely observed except in the strain JF 03-4F, and no conidia were observed forming at the tips of pseudohyphae or hyphae on MEA even after 3 weeks. No characteristic asexual or sexual structures of *Exophiala* species were observed in either strain on any media or conditions tested on *E. viscosa*.

The other closest relative of *E. viscosa* is *E. sideris*. Multiple strains of *E. sideris* were isolated from primarily arsenate and other metal-contaminated locations, and the holotype strain is isolated from *Sorbus aucuparia* berries in The Netherlands (Seyedmousavi *et al.* 2011). These specific locations and environments are distinct from those in which *E. viscosa* has so far been recovered. The differences between a BSC environment and a berry or rock surface would be the permanence of the location (in reference to the berry surface) and the abundance and make-up of microbial neighbors (i.e. cyanobacteria, algae, specific lichens, and bacteria) (Nunes da Rocha *et al.* 2015; Couradeau *et al.* 2019). Additionally, while arsenate was not a metal tested on *E. viscosa*, the other metals tested on both strains of this fungus did not indicate increased metal resistance, and showed less resistance to CoCl_2_, AgNO_3_, and CuCl_2_ than *S. cerevisiae* (Table 9). Neither sexual nor asexual structures were observed in *E. viscosa* on all media and conditions tested including on PDA after 30 days, whereas *E. sideris* readily forms conidia on PDA (Seyedmousavi *et al.* 2011). Attempts at inducing asexual or sexual development for both *E. viscosa* strains were performed using established methods: modified Leonian's agar from Untereiner (1994), Untereiner and Naveau (1999), and Potato dextrose agar via methods from Seyedmousavi *et al.* (2011), but were not successful.

The isolates described here are the first described instance of this fungus being cultured. This is only the second instance of an *Exophiala* species being isolated from a BSC, the first being *E. crusticola* (Bates *et al.* 2006). The ITS sequences of *E. viscosa* and *E. crusticola* have 82.904% identity (Supplementary File 1), and therefore, they are not the same species, which is supported by the phylogenetic tree in Fig. 2. It is also the first instance of an *Exophiala* species being isolated from a BSC in Canada, as opposed to the Western United States where *E. crusticola* was isolated. In terms of other instances of *E. viscosa*'s presence in biological samples, according to BLAST results of *E. viscosa*'s ITS region on NCBI, there has been one instance of another 100% identical fungal DNA from a rocks surface with the accession number AY843177. Unfortunately, this fungal DNA is from an unpublished article according to NCBI. Additional fungal ITS ribosomal DNA that closely matched *E. viscosa*'s via BLAST on NCBI with 99.26% identity were: MZ017038, MZ016619, MZ016618, all of which were DNA samples labeled “Fungal sp.” from *Pinus ponderosa* either roots or needles from Arizona (USA) (Bowman and Arnold 2021).

#### Taxonomy and species descriptions of *E. viscosa*

***E. viscosa*** E.C. Carr, S.D. Harris, **sp. nov.**

MycoBank MB845198

*Typification:* Canada. British Columbia: Adjacent to Fraser-Fort George H, Jackman Flats Provincial Park (Lat.: 52.95; Long.: −119.416; Alt.: 783), in BSC sample, sampled June 2015, *S. Harris*, isolated July 2015, *E. Carr*. Holotype culture strain JF 03-3F Goopy: CBS 148801. Additional strains JF 03-4F Slimy: CBS 148802.

*GenBank*: Whole genome = JALDXI000000000.1

*NCBI Taxonomy ID*: 2486360

*Etymology*: *E. viscosa* was colloquially referred to as “Goopy” due to the nature of its yeast morphology lending to a very goopy colony. Accordingly, we have formally named it *E. viscosa* for the Latin term for viscous.

*Sexual and asexual morph*: not observed.

*Culture characteristics:* Hyphae not observed in liquid culture, aerial pseudohyphae in colonies observed by day 30 in MEA, primarily found in yeast form in liquid MEA and YPD mediums (23°C) but will form pseudohyphae as well. Cells in MEA liquid culture are ellipsoid in shape, 3.9 μm ± 0.55 μm wide and 5.66 μm ± 0.62 μm long (*n* = 40 cells measured). Cell walls start off thin and become thicker over time. In the pseudohyphal form on solid MEA, budding occurs at 180° or 90° from the mother cell's origin, or at acute angles from the previous bud site. Cells either maintain the size and shape of yeast form as pseudophyphal cells bud out for 4 buds and then begin to elongate, or the buds begin to elongate from the first bud and continue to elongate with each linear budding. Colony morphology all at 23°C with a 12 hour light/dark cycle: on MEA colonies are initially shiny dark grayish brown and smooth to curled, then develops a rainbow sheen with a colony size of 5.2–6.6 mm by day 14, additionally dark supernatant is observed in liquid MEA culture starting on day 3; on malt extract agar with vitamins and trace elements (MAG) colonies are sooty black and glossy with smooth colony edges forming a dome shape, and 6.3–8.1 mm growth is observed on day 14; on MN(nitrate with salts) + *N*-acetyl glucosamine as the carbon source, the colony is matte sooty black smooth edged and grows to 2.7–2.8 mm in 14 days; on MNV, the colony is glossy raisin black with smooth edges and smooth top, colonies grow to 4.7–5.3 mm in 14 days; on PDA colonies are glossy sooty black with smooth edges and grow to 5.6–5.8 mm in 14 days; on Spider media, the colonies are sooty black and irregularly shaped with a size of 3.0–4.5 mm in 14 days, additionally a red pigment Is observed surrounding the colonies in the media; on V8 medium colonies are glossy sooty black and irregularly shaped, growth reaches 7.5–8.1 mm in 14 days; on YPD colonies are blackish brown (1) and matte with round to irregular side morphologies and undulate top morphology, dark excretions are observed coming from the colonies and excessively dark supernatant is observed in liquid culture starting on day 2, colony growth reaches 6.1–6.2 mm in 14 days. Color descriptions used above come from Ridgway (1912).

*Cardinal temperatures*: Growth is capable at temperatures from 4°C to 28°C, and optimal growth is at 23°C, growth is not capable at or above 37°C.

*Additional specimen examined*: Canada. British Columbia: Adjacent to Fraser-Fort George H, Jackman Flats Provincial Park, in BSC sample, sampled June 2015, *S. Harris*, isolated July 2015, *E. Carr*. Culture *E. viscosa* Slimy JF 03-4F CBS 148802.

*Notes*: Support for *E. viscosa* as a novel species stems from molecular multi-locus phylogenetic analyses both for marker genes and single-copy genes, morphological comparisons of micro- and macromorphology to phylogenetically related species, ecology, and biogeography of the novel species. *E. viscosa* is phylogenetically placed on all analyses as related to but separate from *E. sideris* with 100% posterior probability in all Bayesian analyses, and bootstrap values above 95 in all Maximum likelihood analyses (Figs. 2 and 3 and Supplementary Fig. 2). The highest matching species to *E. viscosa*'s ITS sequence was *E. nigra*, however, marker gene phylogenies place it as polyphyletic to *E. viscosa* (Fig. 2 and Supplementary Fig. 2). Morphologically, *E. viscosa* is distinct in that it excreted melanin into its surrounding environment, especially on high organic nitrogen substrates such as YPD or on MEA if grown for longer than 2 weeks, which is not noted in other *Exophiala* species. Additionally, it forms a rainbow sheen when grown on MEA, which is not observed in *E. sideris* or *E. nigra* (Seyedmousavi *et al.* 2011; Moussa *et al.* 2017). Sexual or asexual structures were not observed in *E. viscosa* on all media and conditions tested, whereas *E. sideris* readily forms conidia on PDA (Seyedmousavi *et al.* 2011). Both strains of *E. viscosa* are also the first instances of *Exophiala* species being isolated from Canadian soils and biological soil crusts, and the only other *Exophiala* species that have been previously isolated from a BSC was *E. crusticola* (Bates *et al.* 2006).

### Genome description and gene content

Genome sequencing, assembly, and annotation were performed by DOE JGI, and their genomes and transcriptomes are deposited on the Mycocosm website under their genus species names. All accession numbers for both strains are listed in Table 5. The genome assembly sizes of these 2 novel *Exophiala* strains are 28.29 Mbp for *E. viscosa* JF 03-3F and 28.23 Mbp for *E. viscosa* JF 03-4F (Table 6). These sizes are similar to other yeasts in the genus *Exophiala* and are only a bit smaller than their closest relative *E. sideris* (29.51 Mbp). Their predicted gene contents are 11,344 for *E. viscosa* JF 03-3F and 11,358 for *E. viscosa* JF 03-4F (Table 6). *E. sideris* appears to possess longer genes, transcripts, exons, and introns compared to *E. viscosa* JF 03-4F and *E. viscosa* JF 03-3F, which could contribute to the gene number and genome size differences (Table 6). *E. viscosa* JF 03-3F and JF 03-4F contain a median percent of GC content (51.3%) amongst the *Exophiala* species listed in Table 6.

**Table 5.** Accession numbers for all databases containing *E. viscosa* JF 03-3F and JF 03-4F's data.

Database ID numbers	*E. viscosa* JF 03-3F	*E. viscosa* JF 03-4F
WGS Accession GenBank	JALDXI000000000	JALDXH000000000
WGS Accession version used	JALDXI010000001.1	JALDXH000000001.1
Transcriptome Accession	PRJNA501636	PRJNA501637
BioProject	PRJNA500555	PRJNA500554
BioSample	SAMN10366861	SAMN10366859
SRA	SRS4089839	SRS4090708
DOE JGI GOLD project ID	Gp0220461	Gp0220460
CBS strain number	148801	148802
MycoBank number	MB845198	MB845199

**Table 6.** Genome descriptions of the novel *Exophiala* species and related taxa. Information gathered from JGI's Mycocosm.

	*E. viscosa* JF 03-3F CBS148801	*E. viscosa* JF 03-4F CBS148802	*E. sideris* CBS121828	*E. spinifera* CBS89968	*E. xenobiotica* CBS118157	*E. oligosperma* CBS72588	*E. dermatitidis* UT8565
Accession #s	PRJNA500555	PRJNA500554	PRJNA325799	PRJNA325800	PRJNA325801	PRJNA325798	PRJNA225511
Genome assembly statistics							
Genome assembly size (Mbp)	28.29	28.23	29.51	32.91	31.41	38.22	26.35
Number of contigs	35	27	69	143	64	287	10
Number of scaffolds	30	17	5	28	15	143	10
Number of scaffolds ≥2Kbp	25	17	5	20	11	129	10
Scaffold N50	4	5	2	4	3	5	4
Scaffold L50 (Mbp)	2.24	2.89	7.9	3.79	5.04	3.39	3.62
Number of gaps	5	10	64	115	49	144	0
% of scaffold length in gaps	0.00%	0.00%	0.10%	0.10%	0.10%	0.80%	0.00%
Three largest scaffolds (Mbp)	5.39, 4.56, 2.49	4.57, 3.58, 2.93	9.94, 7.90, 7.15	6.18, 3.93, 3.92	5.55, 5.20, 5.04	4.47, 4.29, 4.12	4.25, 4.22, 3.71
GC content (%)	51.3	51.3	50.7	51.7	51.5	50.4	51.5
**Gene statistics**							
Number of genes	11344	11358	11120	12049	13187	13234	9562
Gene length (bp, average)	1840	1844	2044	1593	2072	2090	2237
Gene length (bp, median)	1666	1671	1823	1415	1845	1879	1923
Transcript length (bp, average)	1740	1746	1933	1483	1941	1949	2122
Transcript length (bp, median)	1564	1574	1710	1311	1710	1735	1794
Exon length (bp, average)	705	707	776	621	738	743	896
Exon length (bp, median)	410	414	452	339	430	426	513
Intron length (bp, average)	70	69	74	81	80	85	85
Intron length (bp, median)	56	56	56	61	57	61	62
Protein length (aa, average)	488	489	500	494	492	488	501
Protein length (aa, median)	428	430	434	437	431	429	429
Exons per gene (Average)	2.47	2.47	2.49	2.39	2.63	2.62	2.37
Exons per gene (median)	2	2	2	2	2	2	2

Although the morphology and development of *E*. *viscosa* are seemingly much less complex compared to other Eurotiomycete fungi (e.g. *Aspergillus nidulans*), there are few differences in the complement of genes that are assigned to these processes when these fungi are compared. For example, compared to *A*. *nidulans*, differences include losses of a velvet regulator (VosA) as well as a class of hydrophobins (RodA, DewA), along with the gains of additional cryptochrome/photolyases (CryA) and an NADPH oxidase (NoxB) (Supplementary File 3). This pattern suggests that *E. viscosa* might harbor additional morphological or developmental complexity that has yet to be observed. Notably, results from the automated and manual annotation of the genome sequences reveal no striking differences in numerous traits (e.g. morphogenetic or developmental functions, gene clusters associated with secondary metabolism, CAZymes, transcription factors, cytochrome p450 complement) compared to other sequenced and annotated genomes from the members of Chaetothyriales (Teixeira *et al.* 2017). Additional details of both species’ genome sequences, including results from automated annotation (e.g. GO, KEGG, PFAM domains, etc.) can be found on their respective pages on the JGI Mycocosm website (https://mycocosm.jgi.doe.gov/EurotioJF033F_1/EurotioJF033F_1.home.html; https://mycocosm.jgi.doe.gov/EurotioJF034F_1/EurotioJF034F_1.home.html).

A synteny dot plot of *E. viscosa* JF 03-3F vs *E. viscosa* JF 03-4F was performed to determine if these strains had nonsyntenic regions which could potentially contribute to their morphological differences. The resulting dot plot showed that most of their genomes are syntenic and 98.31% of their genomes are greater than 75% matching (Supplementary Fig. 5). However, there are short regions that have rearrangements and some rearrangements with inversions implying a degree of nonsynteny. Additional dot plots were created to compare these strains to *E. sideris* to further determine if there was similar genome synteny that was observed in the *E. viscosa* strains. Dot plots of both strains of *E. viscosa* aligned to *E. sideris* resulted in very low percent matching with 90.41% for 03-3F matching <25% of the sequences of *E. sideris* and 90.51% for 03-4F matching <25% of the sequences of *E. sideris*. Additionally, most of the genome alignments were nonsyntenic to *E. sideris*, containing many inversions and rearrangements, with only short regions of synteny (Supplementary Figs. 6 and 7).

### Mating type

*Exophiala* species are one of the many fungal genera whose mating capabilities remain incompletely understood and vary across the genus (Teixeira *et al.* 2017). The closest species to *E. viscosa* JF 03-3F and *E. viscosa* JF 03-4F with its mating type identified is *E. sideris,* within which both mating types have been characterized as a single fused gene (Teixeira *et al.* 2017). Given the known order of genes in regions flanking the *MAT* locus in *Exophiala* species, we used comparative approaches to determine the mating identities of the sequenced *E. viscosa* JF 03-3F and *E. viscosa* JF 03-4F isolates, and to look for possible evidence of homothallism that is also presumed in *E. sideris*. Homologs of the genes *APN2*, *SLA2*, *APC5*, *COX13*, and *MAT1* (*MAT1-1/alpha*) in *E. viscosa* JF 03-3F and *E. viscosa* JF 03-4F were identified via BLAST searches. We found that *APN2* and *SLA2* flank the *MAT* locus of both species, and they also contain the Herpotrichielleaceae-specific mating gene *MAT1-1-4* (Supplementary Fig. 8). These results are not surprising, in that this is the exact same order as these genes in *E. sideris*. Interestingly, as Teixeira *et al.* indicate, in *E. sideris* the *MAT* gene is a fused *MAT1-1/MAT1-2* gene with the HMG domain in the middle splitting the α-box in half (2017). When the MAT protein sequences of *E. viscosa* JF 03-3F and *E. viscosa* JF 03-4F are aligned with the *MAT* gene of *E. sideris*, we see 83.79% homology between their protein sequences indicating that these fungi also contain a fused *MAT* gene (Supplementary Fig. 8). We were able to confirm that the HMG domain was inserted within the α-box of *E. viscosa* via NCBI's conserved domains visualizer, where residues 75–206 were the HMG domain within the *MAT1-1* protein sequence. This fused *MAT* gene (*MAT1-1-1*) presumably confers homothallic mating behavior, although this has not been confirmed experimentally in any of these 3 fungi. Additionally, neither *E. viscosa* JF 03-3F nor *E. viscosa* JF 03-4F contain an additional gene between *APN2* and *MAT1-1-4* as is found in *E. sideris* (Teixeira *et al.* 2017). Furthermore, *COX13* and *APC5* are about 7,000 bp downstream of the MAT locus in both species, but *COX13* is on the reverse strand in *E. viscosa* JF 03-4F compared to being on the forward strand in *E. viscosa* JF 03-3F (Supplementary Fig. 8).

#### Phenotyping of *E. viscosa* JF 03-3F and *E. viscosa* JF 03-4F

To further understand the morphology, growth capabilities, and stress responses of *E. viscosa* JF 03-3F and *E. viscosa* JF 03-4F, we performed multiple phenotyping experiments. These experiments intended to provide a broader perspective on the potential adaptations that would support the ability of these fungi to successfully colonize BSCs and other extreme niches.

### Budding patterns

Due to similarities in their colony morphologies, the extent to which this was reflected in their respective budding patterns was assessed. Using protocols adapted from Mitchison-Field *et al.* (2019) we were able to perform more detailed time-lapse microscopy of their budding patterns (Supplementary Fig. 1). From this we were able to observe dramatic differences in the budding types of *E. viscosa* JF 03-3F and *E. viscosa* JF 03-4F.

*E. viscosa* JF 03-3F buds with round cells in initially a distal fashion where the new bud forms 180° from the mother cell origination site, but also form new buds at a ∼90° angle from where the mother bud was formed in a proximal manner (Fig. 6; Supplementary Video 4). *E. viscosa* JF 03-4F on the other hand forms elongated cells in a distal manner, forming long chains of cells instead of clusters, with axial buds forming at later timepoints (Fig. 6; Supplementary Video 2). These morphological differences in budding patterns influence the way these 2 strains grow in a shaking flask. For example, *E. viscosa* JF 03-4F forms more elongated cells and buds distally which although it does not result in the formation of hyphae, still creates larger clumps of cells that are not easily pipetted. However, because *E. viscosa* JF 03-3F forms rounder cells and buds with both distal and proximal patterns, it does not form extensive clumps in a shaking flask and is more easily pipetted. *E. viscosa* JF 03-4F also forms more extensive biofilms at the liquid-air interface than *E. viscosa* JF 03-3F, likely also due to the elongated cell morphology (Figs. 4i and 5i).

### Growth of *E. viscosa JF 03-3F* and *E. viscosa JF 03-4F* on different media

**Fig. 7.**
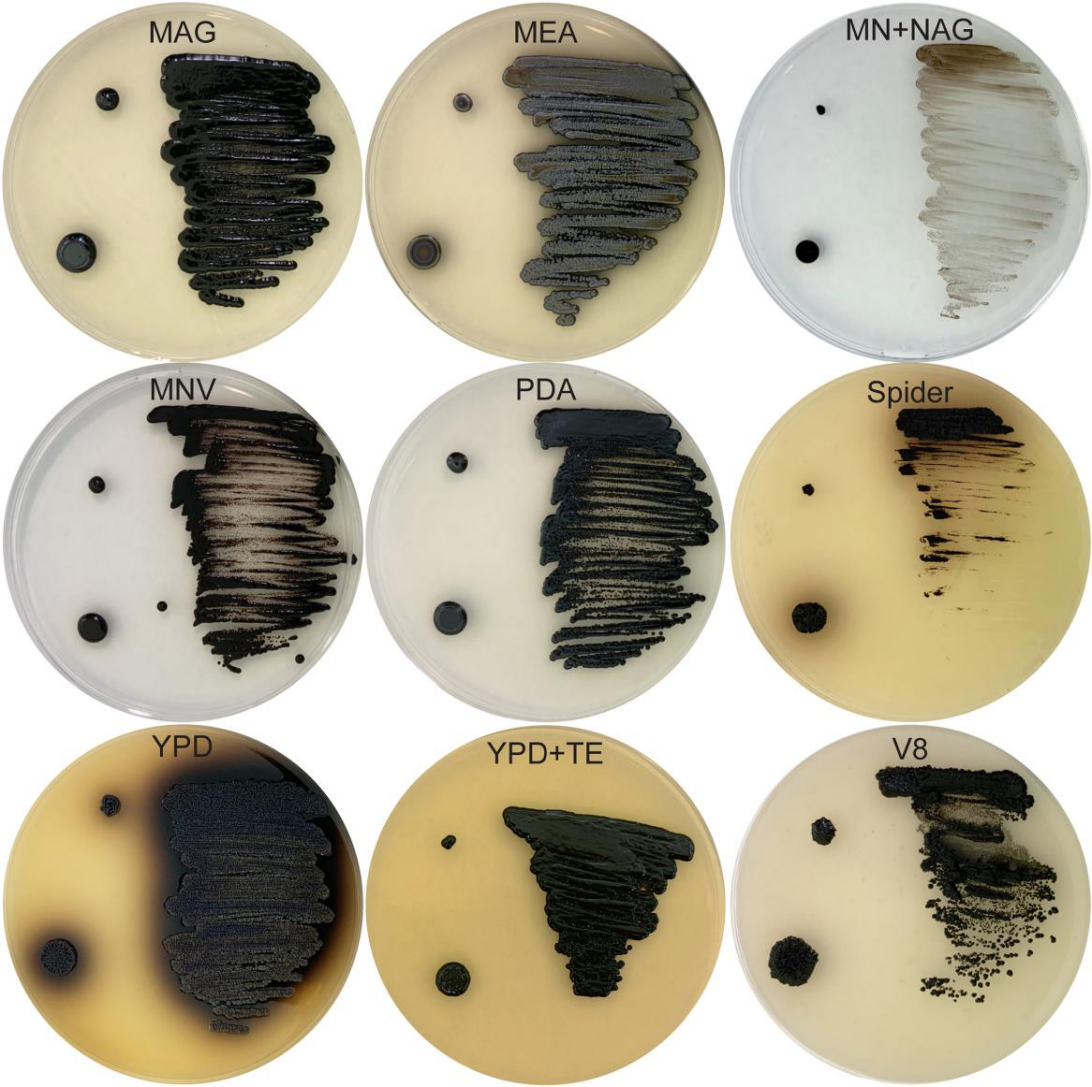
Growth of *E. viscosa* JF 03-3F on different media types grown at 22°C for 14 days, plated as a toothpick prick (top left), 5 μL spot (bottom left), and loop streak patterns (pictures reduced in contrast and lightened to see details; see Supplementary Fig. 18 for unedited photos). *E. viscosa* JF 03-3F was capable of growth on all medias tested, but growth on MN + NAG showed the least amount of growth. PDA, MAG, and MNV allowed for very shiny and dark black colony growth. Growth on V8 medium shows potential for saprotrophic growth on plant material. Colorful red excretion was observed on Spider media and a lot of dark brownish-black excretion on YPD. Addition of TE to YPD prevents excretion of melanin into the media. Growth on MEA resulted in a rainbow iridescent shine on both the streaked-out growth and the 5 μL spot. Images adjusted for brightness and contrast for easier visualization, original images in Supplementary Fig. 18.

**Fig. 8.**
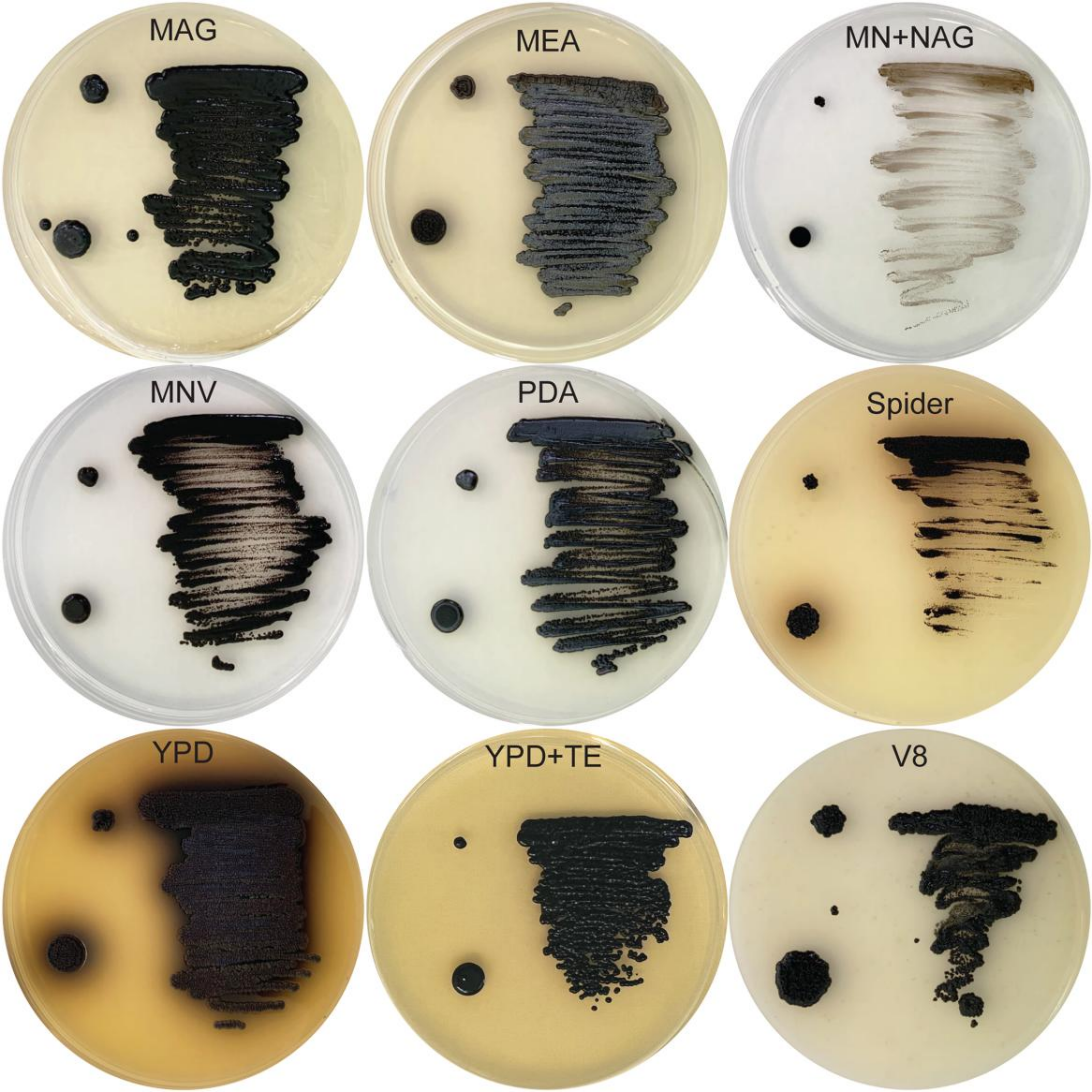
Growth of *E. viscosa* JF 03-4F on different media types grown at 22°C for 14 days, plated as a toothpick prick (top left), 5 μL spot (bottom left), and loop streak patterns (pictures reduced in contrast and lightened to see details; see Supplementary Fig. 18 for originals). *E. viscosa* JF 03-4F was capable of growth on all medias tested, but growth on MN + NAG showed the least amount of growth. PDA, MAG, and MNV allowed for very shiny and dark colonies to form. Growth on V8 medium shows potential for saprotrophic growth. Colorful excretions were observed on both Spider media and YPD. Addition of TE to YPD prevents excretion of melanin into the media. Growth on MEA shows an iridescent shine on the streaked-out growth. Images adjusted for brightness and contrast for easier visualization, original images in Supplementary Fig. 18.

Growth of *E. viscosa* JF 03-3F and *E. viscosa* JF 03-4F on a variety of different media was done to evaluate growth requirements and their impact on pigmentation (Figs. 7 and 8; unedited photos in Supplementary Fig. 18). The media used are described in Table 1, and include MAG, MEA, MN + NAG, MNV, PDA, Spider, YPD, YPD + TE, and V8. The addition of Vitamin mix (Table 1) to any medium, but specifically to MAG and MNV, caused the colonies of both isolates to become much shinier, blacker (vs browner), and more yeast morphology. Growth on MEA caused the formation of a rainbow iridescent sheen (Figs. 4k, 5k, 7, and 8), which is not seen on any other medium. Spider medium and YPD caused the formation of a colored halo around colonies of both strains. However, the halo around colonies grown on YPD is much darker and extends further than on Spider medium, and *E. viscosa* JF 03-3F showed a more extensive halo than *E. viscosa* JF 03-4F. When TE are added to YPD, the formation of a dark halo is inhibited in both strains. The ability of both strains to grow on a V8 medium implies that they can use cellulosic material as a carbon source. Overall, colony growth and pigmentation were similar across media types for both strains (Figs. 7 and 8).

### Optimal wavelength for OD measurements

Serial dilutions (1:1 to DF = 512) of both *E. viscosa* JF 03-3F and *E. viscosa* JF 03-4F were read in a wavelength range of 300–700 nm at 10 nm intervals. The OD results of those dilutions were then compared to the percent dilution at 25% dilution and below, the percent dilution at 50% and below, cell counts at 2.19 × 10^6^ cells/mL and below, cell counts at 4.38 × 10^6^ cells/mL and below, and the consistency of the actual percent dilution at 12.5% dilution and below. The resulting R^2^ values showed that the optimal wavelength range for reading ODs for these strains is between 410–420 nm (Supplementary Table 1). To reduce the potential effect pure melanin in the supernatant might have on the OD results, we decided to choose the higher wavelength of 420 nm for our OD reads.

### Carbon and nitrogen utilization

**Fig. 9.**
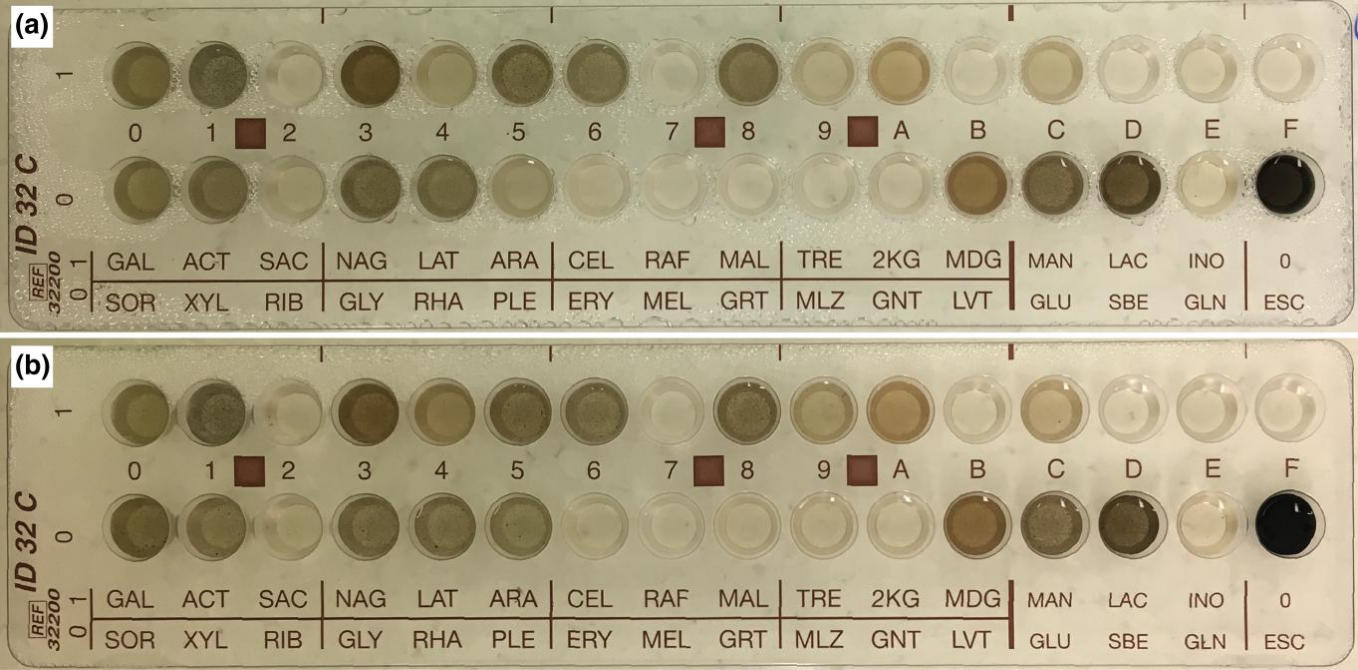
Growth of a) *E. viscosa* JF 03-3F and b) *E. viscosa* JF 03-4F on C32 strips for determining carbon utilization. Both species were capable of using the same carbon sources, though some variations are seen. *E. viscosa* JF 03-4F was better at growing on palatinose (PLE), trehalose (TRE), potassium 2-ketogluconate (2KG), and mannitol (MAN) than *E. viscosa* JF 03-3F. *E. viscosa* JF 03-3F was not better at growing on any carbon sources than *E. viscosa* JF 03-4F.

Carbon source utilization was determined using Biomerieux C32 carbon strips, which are typically used for the identification of human pathogens (Tragiannidis *et al.* 2012). Following the protocols provided by the vendor, we were able to show that *E. viscosa* JF 03-3F and *E. viscosa* JF 03-4F can utilize a wide range of carbon sources. Triplicates were performed on these strips to ensure the results were uniform and representative. Overall, a variety of carbon sources supported the robust growth of both strain (e.g. D-glucose, L-sorbose, D-galactose, *N*-acetyl glucosamine, D-sorbitol, D-xylose, glycerol, L-rhamnose, L-arabinose, D-cellobiose, and maltose), and there are only a few quantitative differences in utilization patterns (Fig. 9 and Tables 7 and 8). Carbon sources that could not support the growth of either strain included D-raffinose, D-melibiose, methyl-aD-glucopyranoside, and D-lactose. Genome annotation did not provide an obvious explanation for this observation (Supplementary File 3). Both strains were resistant to cycloheximide and were capable of producing a black color in the presence of esculin ferric citrate (positive for esculin metabolism) (Fig. 9). Notably, for both *E. viscosa* JF 03-3F and *E. viscosa* JF 03-4F, growth on some carbon sources, particularly sorbose, levulinic acid, and N-acetyl glucosamine, lead to enhanced pigmentation (Fig. 9).

**Table 7.** Carbon source utilization values of *E. viscosa* JF 03-3F.

*E. viscosa* JF 03-3F		# 1	# 2	# 3	Average	Score
D-galactose	GAL	4	5	4	4.3	+
D-sorbitol	SOR	4	4	4	4.0	+
Actidione (cycloheximide)	ACT	5	4	4	4.3	+
D-xylose	XYL	4	4	4	4.0	+
D-saccharose (sucrose)	SAC	1	1	1	1.0	–
D-ribose	RIB	2	2	2	2.0	V
*N*-acetyl glucosamine	NAG	4	5	4	4.3	+
Glycerol	GLY	4	4	4	4.0	+
Lactic acid	LAT	3	3	3	3.0	V
L-rhamnose	RHA	4	4	4	4.0	+
L-arabinose	ARA	4	4	4	4.0	+
PLE	PLE	3	3	3	3.0	V
D-cellobiose	CEL	4	4	4	4.0	+
Erythritol	ERY	2	2	2	2.0	V
D-raffinose	RAF	1	1	1	1.0	–
D-melibiose	MEL	1	1	1	1.0	–
D-maltose	MAL	4	4	4	4.0	+
Sodium glucuronate	GRT	2	2	2	2.0	V
D-trehalose	TRE	3	3	3	3.0	V
D-melezitose	MLZ	2	2	2	2.0	V
Potassium 2-ketogluconate	2KG	4	3	4	3.7	+
Potassium gluconate	GNT	2	2	2	2.0	V
Methyl-aD-glucopyranoside	MDG	1	1	1	1.0	–
Levulinic acid (leuvulinate)	LVT	4	3	4	3.7	+
D-mannitol	MAN	3	3	3	3.0	V
D-glucose	GLU	5	5	5	5.0	+
D-lactose	LAC	1	1	1	1.0	–
L-sorbose	SBE	5	5	5	5.0	+
Inositol	INO	2	2	2	2.0	V
Glucosamine	GLN	1	1	1	1.0	–
No substrate	No	1	1	1	1.0	–
Esculin ferric citrate	ESC	+	+	+	+	Black +

**Table 8.** Carbon source utilization values of *E. viscosa* JF 03-4F.

*E. viscosa* JF 03-4F		# 1	# 2	# 3	Average	Score
D-galactose	GAL	4	4	4	4.0	+
D-sorbitol	SOR	5	5	5	5.0	+
Actidione (cycloheximide)	ACT	4	4	4	4.0	+
D-xylose	XYL	4	4	4	4.0	+
D-saccharose (sucrose)	SAC	2	2	2	2.0	V
D-ribose	RIB	2	2	3	2.3	V
*N*-acetyl glucosamine	NAG	4	4	4	4.0	+
Glycerol	GLY	4	4	4	4.0	+
Lactic acid	LAT	3	3	3	3.0	V
L-rhamnose	RHA	4	4	4	4.0	+
L-arabinose	ARA	4	4	4	4.0	+
PLE	PLE	4	4	4	4.0	+
D-cellobiose	CEL	4	4	4	4.0	+
Erythritol	ERY	1	2	2	1.7	V
D-raffinose	RAF	1	1	1	1.0	–
D-melibiose	MEL	1	1	1	1.0	–
D-maltose	MAL	4	4	4	4.0	+
Sodium glucuronate	GRT	2	2	2	2.0	V
D-trehalose	TRE	4	3	4	3.7	+
D-melezitose	MLZ	2	2	2	2.0	V
Potassium 2-ketogluconate	2KG	3	3	4	3.3	V
Potassium gluconate	GNT	2	2	2	2.0	V
Methyl-aD-glucopyranoside	MDG	1	1	1	1.0	–
Levulinic acid (leuvulinate)	LVT	3	4	3	3.3	V
D-mannitol	MAN	3	3	4	3.3	V
D-glucose	GLU	5	5	5	5.0	+
D-lactose	LAC	1	1	1	1.0	–
L-sorbose	SBE	5	5	5	5.0	+
Inositol	INO	2	2	2	2.0	V
Glucosamine	GLN	2	2	1	1.7	V
No substrate	0	1	1	1	1.0	–
Esulin ferric citrate	ESC	+	+	+	+	Black +

**Fig. 10.**
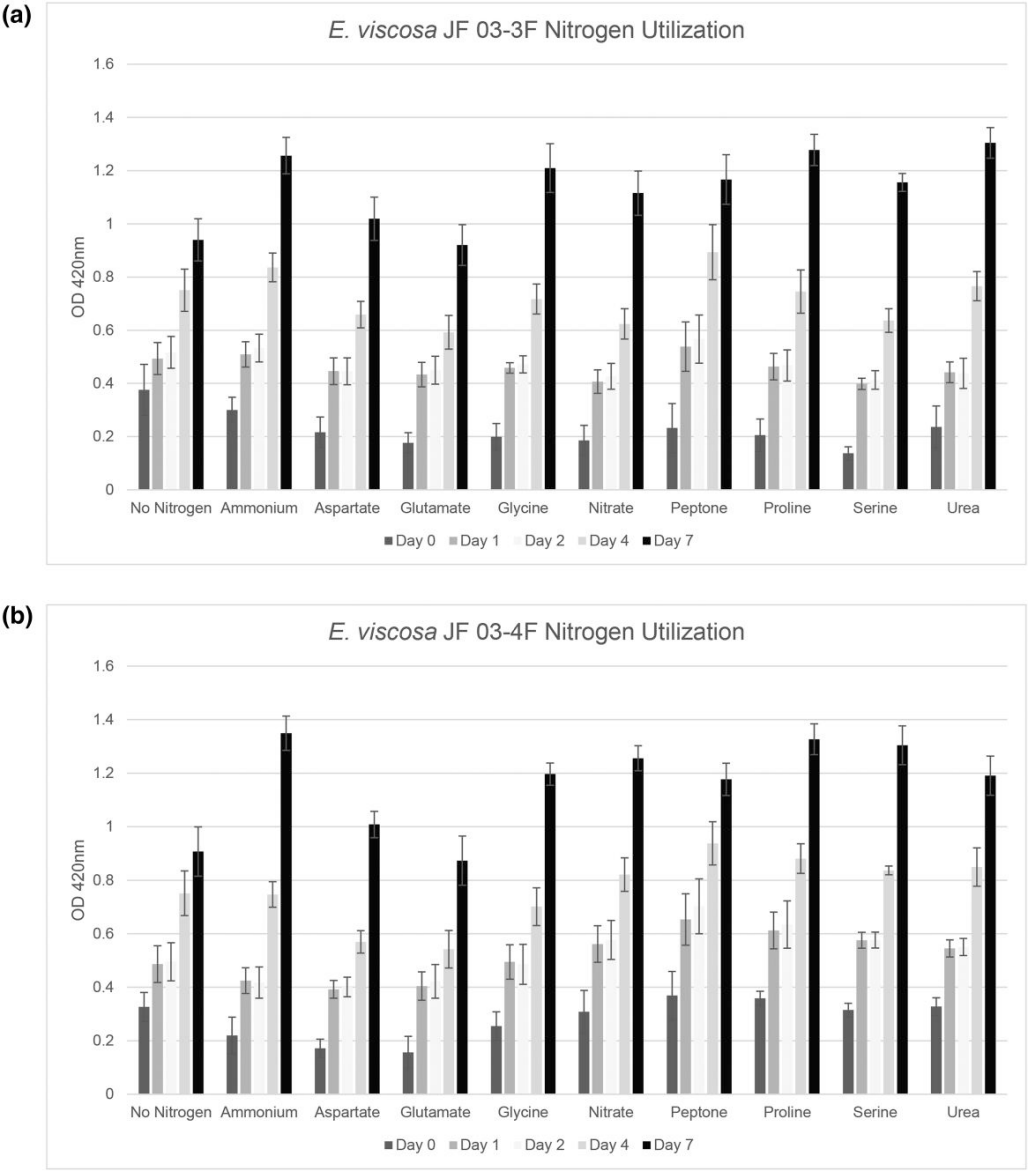
Nitrogen source utilization of a) *E. viscosa* JF 03-3F and b) *E. viscosa* JF 03-4F in liquid culture. Neither species was capable of using aspartate or glutamate as a nitrogen source, as their growth amount were equivalent to no nitrogen. All other nitrogen sources tested were used by both species with varying preference. Ammonium was the preferred nitrogen source for *E. viscosa* JF 03-4F (*P*-value = 0.0007 compared to peptone (*n* = 6)), and *E. viscosa* JF 03-3F preferred urea for a nitrogen source (*P*-value = 0.009 compared to peptone (*n* = 6)). *E. viscosa* JF 03-3F was unable to use aspartate or glutamate as nitrogen sources, but *E. viscosa* JF 03-4F was able to use aspartate as a nitrogen source (*P*-value = 0.03 compared to no nitrogen (*n* = 6)). *P-value of 0.05 > (*n* = 6) compared to peptone positive control, **P-value of 0.01 > (*n* = 6) compared to peptone positive control, ^+^*P*-value of 0.05 > (*n* = 6) compared to no nitrogen negative control.

We were particularly interested in patterns of nitrogen utilization for *E. viscosa* JF 03-3F and *E. viscosa* JF 03-4F given their isolation from a nutrient-depleted niche with extensive nitrogen-fixing bacterial populations. Nine different nitrogen sources were tested: 5 amino acids (aspartate, glutamate, glycine, proline, serine), ammonium tartrate, sodium nitrate, urea, peptone (mixed short chain amino acids) as a positive control, and no nitrogen as a negative control. Both strains are incapable of utilizing aspartate and glutamate as nitrogen sources; *E. viscosa* JF 03-3F has a *P*-value of 0.012 (*n* = 6) for aspartate and 0.0005 (*n* = 6) for glutamate; *E. viscosa* JF 03-4F having a *P*-value of 0.0003 (*n* = 6) for aspartate and.00005 (*n* = 6) for Glutamate (all compared to peptone) (Fig. 10). *E. viscosa* JF 03-3F and *E. viscosa* JF 03-4F differed in their preferred nitrogen sources. Both preferred proline, *P*-value for *E. viscosa* JF 03-3F 0.024 (*n* = 6), *P*-value for *E. viscosa* JF 03-4F is 0.0012 (*n* = 6), over peptone. However, they differ in that *E. viscosa* JF 03-3F most prefers urea *P*-value 0.009 (*n* = 6), while *E. viscosa* JF 03-4F had additional significantly preferred nitrogen sources including serine (*P*-value 0.0064 (*n* = 6)), ammonium (*P*-value 0.0007 (*n* = 6)), and nitrate (*P*-value 0.022 (*n* = 6) (Fig. 10).

### Optimal growth temperature and range of growth temperatures

**Fig. 11.**
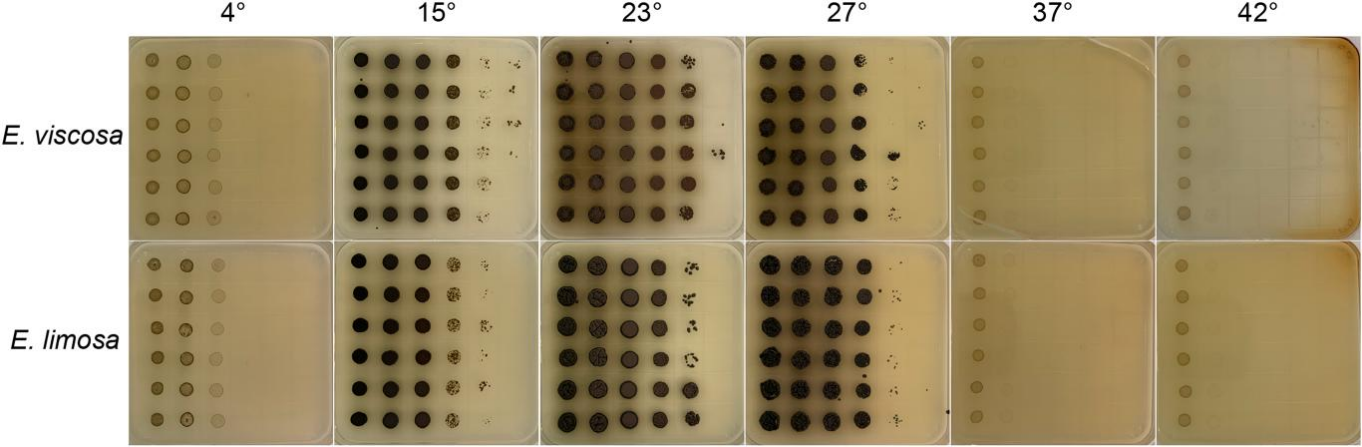
Growth of *E. viscosa* JF 03-3F and *E. viscosa* JF 03-4F at varying temperatures. Neither species was capable of growth at or above 37°C, implying they are not human pathogens. Both species optimally grew at 23°C, but were capable of growth down to 4°C.

*E. viscosa* JF 03-3F and *E. viscosa* JF 03-4F were isolated from an environment that experiences wide seasonal and diurnal temperature changes. As such, we determined the optimal growing temperature for these strains, as well as the limits at which they are capable of survival (cardinal temperatures). Both isolates were serially diluted and spotted onto MEA plates to determine the carrying capacity at different temperatures. Both *E. viscosa* JF 03-3F *and E. viscosa* JF 03-4F were capable of growth at 4°C, 15°C, 23°C, and 27°C, but could not grow at 37°C and 42°C (Fig. 11). Optimal growth temperature of both *E. viscosa* JF 03-3F and *E. viscosa* JF 03-4F was 23°C (Fig. 11). Growth at 4°C was slow, but after a year the isolates both strains formed extensive colonies and flooded the agar with melanin (Supplementary Fig. 9). Although neither strain grew at 37°C, they retained viability as they were able to resume growth following return to 23°C after 2 days of exposure to 37°C (Supplementary Fig. 9). In contrast, the same experiment showed that incubation at 42°C is lethal.

### UV and metal resistance

**Fig. 12.**
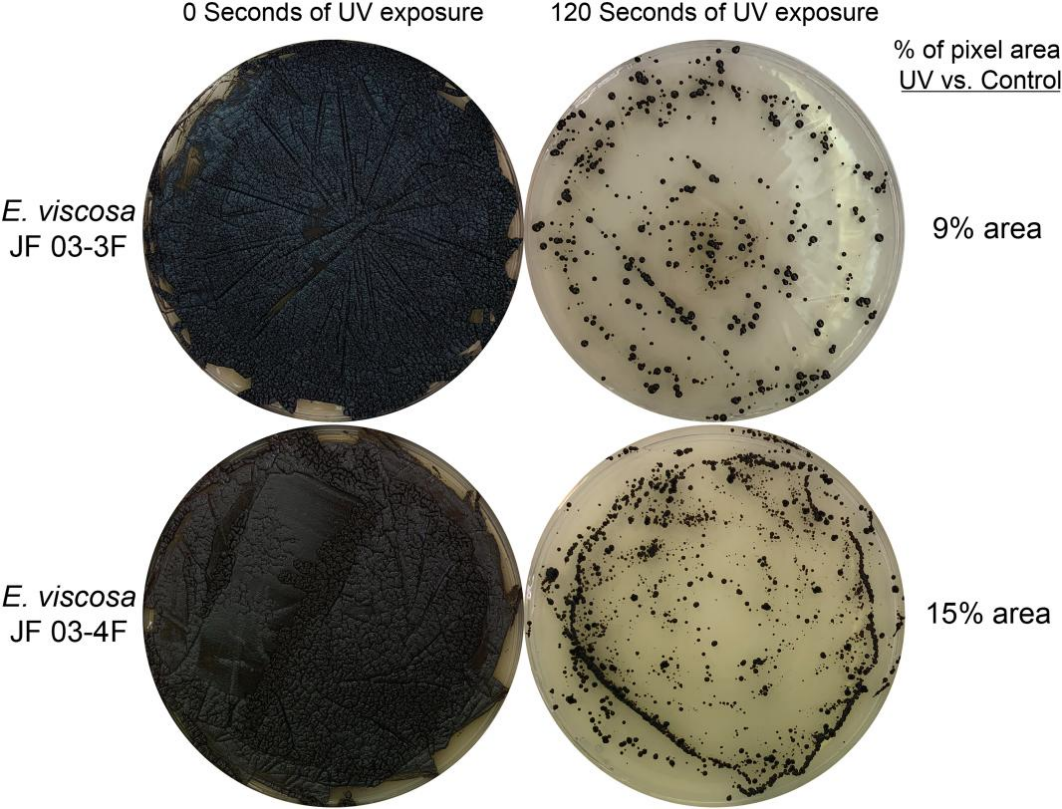
Difference in growth of *E. viscosa* JF 03-3F and *E. viscosa* JF 03-4F with and without exposure to UV light. *E. viscosa* JF 03-3F showed slightly less resistance to UV exposure than *E. viscosa* JF 03-4F. Neither species was mutated from 120 seconds of UV exposure, normally *S. cerevisiae* and *E. dermatitidis* are incapable of growth after the same amount of UV exposure (data not shown).

Melanized fungi are recognized for their resistance to UV light, and the possibility of using ionizing radiation as an energy source (Dadachova *et al.* 2007). To assess the response of *E. viscosa* JF 03-3F and *E. viscosa* JF 03-4F to UV radiation, they were each exposed to a dose (120 seconds of 10,000 μJ/cm^2^ at 254 nm) that was lethal to *S. cerevisiae* and *E. dermatitidis* (data not shown). The same level of exposure did not kill either *E. viscosa* JF 03-3F or *E. viscosa* JF 03-4F (Fig. 12) but did significantly reduce the number of viable colonies. Strikingly, surviving colonies showed no evidence of induced mutagenesis based on the absence of altered morphologies or pigmentation (Fig. 12).

Polyextremotolerant fungi have been previously noted as having increased metal resistances as a result of their melanized cell wall and other adaptations to harsh environments (Gadd and de Rome 1988). To test if these 2 new *Exophiala* spp. possess increased metal resistances when compared to *E. dermatitidis* and *S. cerevisiae*, we impregnated Whatman filter discs with various metals of different concentrations. Diameters of zones of clearing revealed no evidence for enhanced metal resistance to the metals tested (Table 9). On the other hand, both strains appear to be moderately more sensitive to NiCl_2_ and CdCl_2_ (Table 9).

**Table 9.** Diameter of the zone of clearing of *E. viscosa* JF 03-3F, *E. viscosa* JF 03-4F, *E. dermatitidis*, and *S. cerevisiae* with various metals.

Fungus	FeSO_4_ 1 M	CoCl_2_ 0.5 M	AgNO_3_ 0.47 M	NiCl_2_ 1.5 M	CuCl_2_ 1.5 M	CdCl_2_ 10 mM
*E. viscosa* JF 03-3F	1.6 cm	1.5 cm	1.4 cm	3.9 cm	1.3 cm	4.5 cm
*E. viscosa* JF 03-4F	1.8 cm	1.8 cm	1.4 cm	4 cm	1.5 cm	3.9 cm
*E. dermatitidis*	1.3 cm	1 cm	1.5 cm	2 cm	2.5 cm	1.2 cm
*S. cerevisiae*	1 cm	1.9 cm	2 cm	2.1 cm	2 cm	2.5 cm

### Lipid profiles

Both *E. viscosa* JF 03-3F and *E. viscosa* JF 03-4F appear to possess abundant lipid bodies (Supplementary Fig. 4). This observation along with the unique sticky morphology of both strains led us to the idea that they might contain unique or copious amounts of lipids. We performed a Bligh and Dyer lipid extraction followed by TLC to observe patterns of lipid accumulation in *E. viscosa* JF 03-3F and *E. viscosa* JF 03-4F grown on media that contained fermentable or nonfermentable sugars, both with high nitrogen and low nitrogen. These iterations creating 4 unique media types would allow us to cover as much of a spread of lipid changes as possible. *S. cerevisiae* and *E. dermatitidis* were also similarly analyzed. Results from this lipid extraction showed that our 2 new strains did not seem to produce any unique or novel amounts or types of lipids when compared to *S. cerevisiae* and *E. dermatitidis* (Supplementary Fig. 10).

### Melanin production and regulation in *E. viscosa* JF 03-3F and *E. viscosa* JF 03-4F

#### Melanin biosynthesis gene annotation

A defining feature of black yeasts is their pigmentation caused by the accumulation of melanin (Bell and Wheeler 1986). Given the presumed importance of melanin to a polyextremotolerant lifestyle, we are interested in understanding how melanin production is regulated. The first step in this process is to determine the types of melanin that *E. viscosa* JF 03-3F and *E. viscosa* JF 03-4F are capable of producing. To accomplish this, the sequenced and automatically annotated genomes of *E. viscosa* JF 03-3F and *E. viscosa* JF 03-4F were manually annotated using protein sequences for all 3 melanin biosynthetic pathways in *Aspergillus niger* (Teixeira *et al.* 2017). The list of *A. niger* (and 2 *A. fumigatus*) proteins and their homologs in both *E. viscosa* JF 03-3F and *E. viscosa* JF 03-4F are summarized in Table 10. Manual annotation and reverse BLASTP of the melanin biosynthesis pathway genes showed that both *E. viscosa* JF 03-3F and *E. viscosa* JF 03-4F have the genetic capability to produce all 3 forms of known fungal melanins: DOPA-melanin (pheomelanin), DHN-melanin (allomelanin), and L-tyrosine/HGA-derived pyomelanin. Two *A. fumigatus* proteins, hydroxynaphthalene reductase (Arp2) and scytalone dehydratase (Arp1) were also separately BLASTPed against the sequences of *E. viscosa* JF 03-3F and *E. viscosa* JF 03-4F because those protein sequences are not homologous between *A. niger* and *A. fumigatus* (Jørgensen *et al.* 2011). Both *E. viscosa* JF 03-3F and *E. viscosa* JF 03-4F were found to have homologous proteins to both *A. fumigatus* and *A. niger*'s Arp1 and Arp2 (Table 10).

**Table 10.** Annotation of melanin biosynthetic genes for *E. viscosa* JF 03-3F and JF 03-4F. JGI Mycocosm Protein (Prot.) IDs provided for *E. viscosa* JF 03-3F and JF 03-4F homologs.

DHN/Allomelanin pathway								
	*E. viscosa* JF 03-3F Homologs				*E. viscosa* JF 03-4F Homologs			
Gene in *A. niger* (or *A. fumigatus)	Mycocosm Prot. ID #	e-value	Bit score	% ID	Mycocosm Prot. ID #	e-value	Bit score	% ID
**Pks1** An03g05440	580617	0.0E00	4,077	47.0	463165	0.0E00	4,081	47.0
**Ayg1** An14g05350	511449	1.21E-90	852	44.0	494160	1.21E-90	852	43.9
**Arp2** An02g00220	676985	5.72E-93	779	56.5	479993, 453709	5.74E-93	779	56.5, 48.7
*Arp2 *Afu2g17560*	625384	3.28E-48	612	48.4	242806; 441819	3.29E-48, 6.59E-75	612, 598	48.4, 51.3
**Arp1** An08g09920	477931	5.58E-38	429	51.6	210894	5.59E-38	429	51.6
*Arp1 *Afu2g17580*	452087	2.18E-61	496	53.2	275994	2.19E-61	496	53.2
**Abr2** An01g14010	603697, 153763	9.11E-155, 8.05E-142	1,398, 1,235	51.0, 43.9	326274, 72468	9.13-155, 8.53E-143	1,398, 1,235	46.8, 43.3
**Abr1** An14g05370 An01g11120 An15g05520	648725, 437535	1.2E-99, 2.14E-41	1,082, 551	48.4, 38.8	258543, 441397	1.20E-99, 2.14E-41	1,082, 551	48.4, 38.8
	387337, 648725	5.04E-120, 4.05E-46	1,274, 591	54.5, 44.0	92776, 258543	4.81E-122, 4.06E-46	1,289, 591	55.0, 44.0
	648725, 653857	0.0E00, 1.7E-44	2,144, 584	67.0, 42.7	258543, 102128	0.0E00, 3.27E-44	2,144, 582	67.0, 42.7

L-DOPA/pheomelanin pathway								
	*E. viscosa* JF 03-3F Homologs				*E. viscosa* JF 03-4F Homologs			
Gene in *A. niger*	Mycocosm Prot. ID #	e-value	Bit score	% ID	Mycocosm Prot. ID #	e-value	Bit score	% ID
**MelC2** An01g09220 An03g00280	140179, 643161	1.22E-78, 1.33E-49	833, 576	49.3, 40.6	84855, 433643	1.22E-78, 1.34E-49	833, 576	49.3, 40.6
	140179	2.86E-122	1,084	59.1	84855	2.87E-122	1,084	59.1
**MelO** An12g01670								
**McoJ** An12g05810	571417, 437535	1.06E-94, 6.08E-78	1,067, 799	60.1, 52.5	465594, 441397	1.41E-97, 6.10E-78	1,088, 799	61.2, 52.5
**McoM** An16g02020	571417, 580955	1.12E-103, 3.78E-74	1,127, 807	52.6, 45.3	465594, 189119	4.27E-109, 3.80E-74	1,133, 807	53.9, 45.3
**McoD** An11g03580	437535, 580955	2.11E-124, 5.40E-82	1,178, 938	51.2, 49.6	441397, 189119	2.12E-124, 1.59E-82	1,178, 942	51.2, 49.9
**McoG** An08g08450	437535, 580955	1.67E-110, 4.66E-88	1,069, 944	49.1, 47.6	441397, 189119	1.67E-110, 1.35E-88	1,069, 948	49.1, 47.9
**McoF** An05g02340	437535, 382943, 580955	1.79E-121, 4.03E-101, 3.80E-96	1,150, 1,041, 1,004	44.6, 47.4, 50.3	441397, 95636, 189119	1.80E-121, 5.88E-101, 1.10E-96	1,150, 1,040, 1,008	44.6, 47.4, 50.6
**McoN** An01g00860	653857, 649741	1.72E-129, 3.37E-116	1,253, 1,153	57.3, 55.2	102128, 454148	3.27E-129, 7.54E-97	1,251, 1,009	57.1, 52.6
**McoI** An18g02690	653857, 649741	1.96E-154, 1.47E-134	1,471, 1,287	57.0, 53.3	102128, 454148	3.75E-154, 7.08E-122	1,469, 1,193	56.7, 52.8

L-tyrosine degradation/pyomelanin pathway								
	*E. viscosa* JF 03-3F Homologs				*E. viscosa* JF 03-4F Homologs			
Gene in *A. niger*	Mycocosm Prot. ID #	e-value	Bit score	% ID	Mycocosm Prot. ID #	e-value	Bit score	% ID
**Tat** An02g05540	606461	1.22E-169	1,477	54.4	34310	1.22E-169	1,477	54.4
**HppD** An11g02200	623446	0.0E00	1,448	69.7	39306	0.0E00	1,448	69.7
**HmgA** An11g02180	617354, 102643, 403121	0.0E00, 1.05E-92, 1.12E-104	1,789, 971, 922	73, 45.1, 43.7	430149, 483886, 431641	0.0E00, 1.97E-92, 1.12E-104	1,789, 969, 922	73, 44.9, 43.7
**FahA** An11g02170	617341	4.32E-175	1,477	65.5	148504	1.38E-178	1,466	65
**MaiA** An11g02160	213100	1.15E-55	533	51.2	198633	9.47E-57	541	45.3

#### Regulation of melanin production using chemical blockers

Because *E. viscosa* JF 03-3F and *E. viscosa* JF 03-4F possess the genetic capability to produce all 3 forms of fungal melanin, we asked whether they were all being produced simultaneously or if one was preferred for production vs excretion. We used chemical blockers for both L-DOPA-melanin and DHN-melanin to determine the predominant type of melanin produced on MEA and YPD. Kojic acid blocks the production of L-DOPA-melanin, whereas phthalide inhibits the synthesis of DHN-melanin; both are effective at doses of 1, 10, and 100 μg/mL in various fungi (Pal *et al.* 2014).

**Fig. 13.**
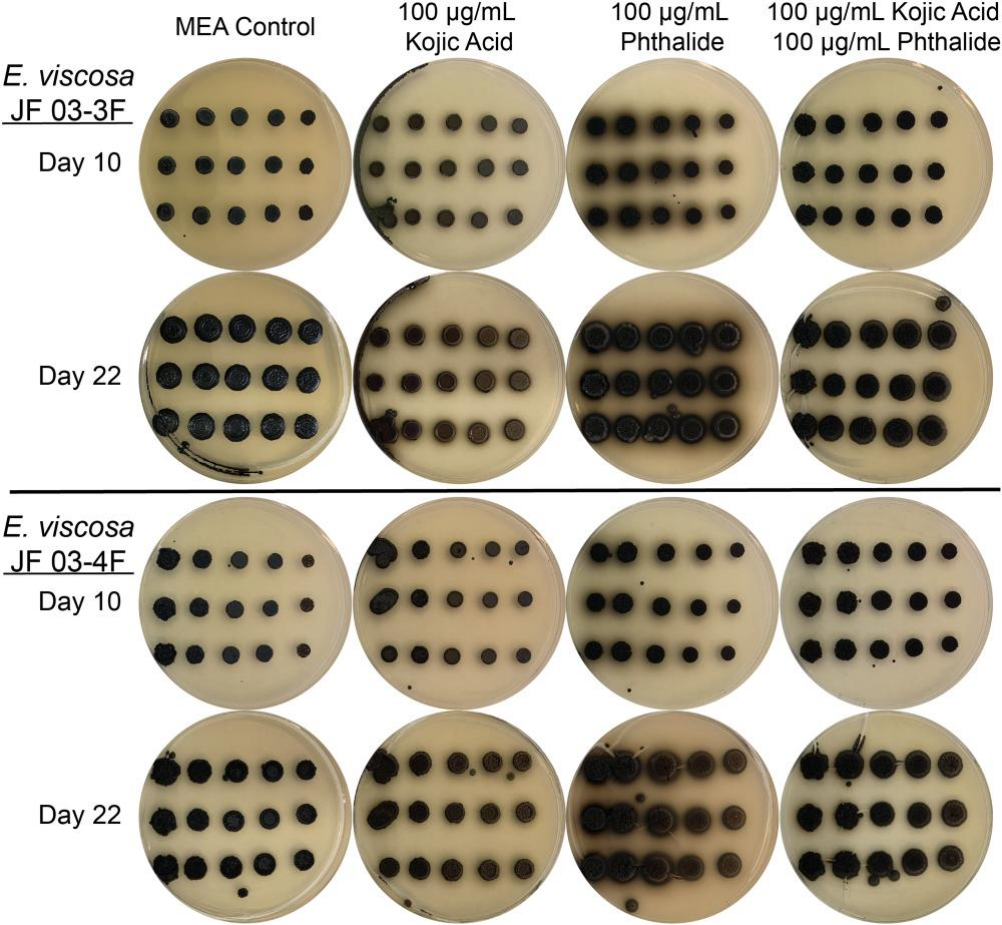
Growth of serial diluted (0×–10,000× with 10× steps) *E. viscosa* JF 03-3F and *E. viscosa* JF 03-4F in the presence of melanin blockers kojic acid and phthalide. Neither chemical melanin blocker was capable of blocking melanin production in either fungus, even when both chemical blockers were used simultaneously. Additionally, use of phthalide on *E. viscosa* JF 03-3F induced melanin secretion on a medium where this does not usually occur. The melanin halo around *E. viscosa* JF 03-3F's colonies on medium containing phthalide was replaced with hyphal growth after 22 days.

First, we used 100 mM of phthalide and 100 mg/mL of kojic acid in a filter disc assay. Placing drug-impregnated filter discs on freshly spread lawns of cells either individually or with both drugs combined did not block melanin production even though the concentrations of the drugs were higher than that of previous studies (Supplementary Fig. 11). Then using Pal *et al.*'s (2014) method of melanin-blocking, we added the highest dosage (100 µg/mL) of phthalide and kojic acid individually and combined to agar-solidified MEA, and both spread a lawn of cells and spotted serially diluted cells onto the plates. Neither assay resulted in blockage of melanin production in *E. viscosa* JF 03-3F or *E. viscosa* JF 03-4F (Fig. 13 and Supplementary Fig. 8). However, addition of 100 µg/mL of phthalide alone did result in their apparent excretion of a dark substance into MEA (Fig. 13). Overall, chemical inhibition of L-DOPA-melanin or DHN-melanin production did not qualitatively reduce colony pigmentation, possibly suggesting that the tyrosine-derived pyomelanin is still being produced.

#### Evaluating the cause of melanin excretion

The appearance of a dark pigment surrounding colonies of *E. viscosa* JF 03-3F and *E. viscosa* JF 03-4F under specific conditions raised the idea that these fungi are able to excrete melanin into their local environment. The presence of dark pigments in the supernatants of liquid cultures lent further support to this.

**Fig. 14.**
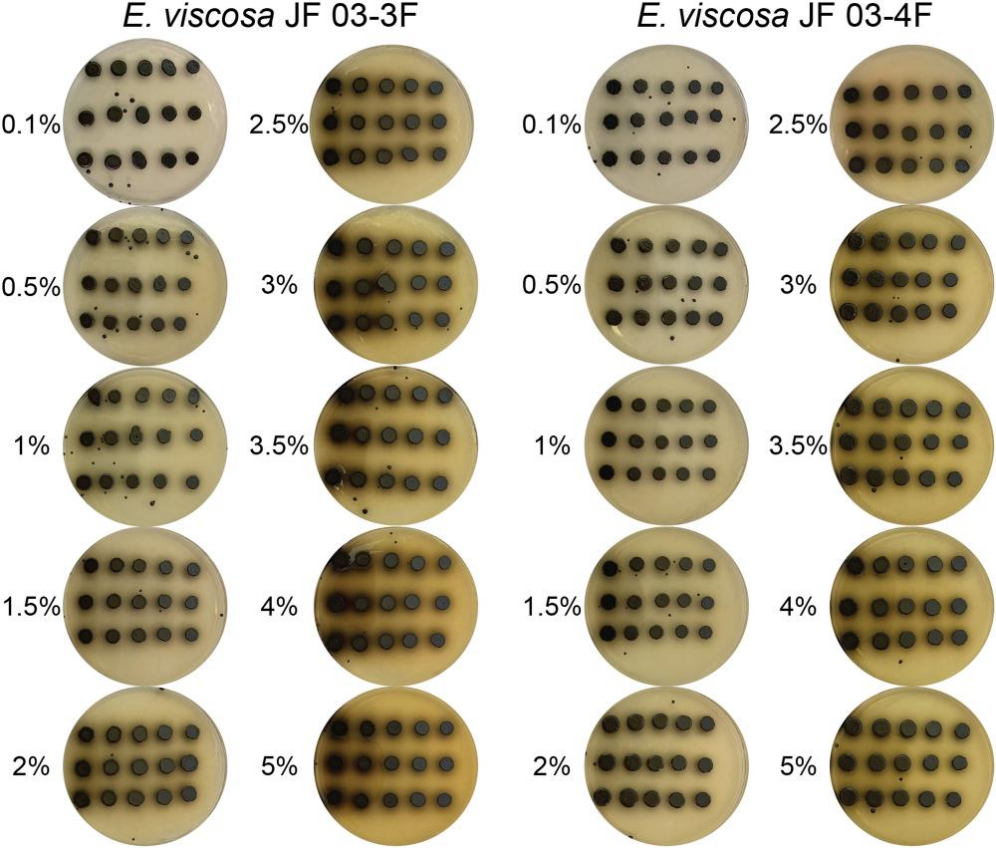
*E. viscosa* JF 03-3F and *E. viscosa* JF 03-4F grown on MEA with increasing amounts of peptone. The higher the amount of peptone in the medium, the more melanin was secreted. *E. viscosa* JF 03-3F started secreting melanin at 2%, and *E. viscosa* JF 03-4F at 4%.

Initial studies were performed to determine which media triggered the release of melanin. Both *E. viscosa* JF 03-3F and *E. viscosa* JF 03-4F were capable of releasing the most melanin and in the shortest growth time on YPD, with *E. viscosa* JF 03-3F seemingly excreting more than *E. viscosa* JF 03-4F (Figs. 7 and 8). Because YPD and MEA only differ by the presence of yeast vs malt extract as well as the percentage of peptone (2% in YPD, 0.2% in MEA), we first determined if the peptone differences impacted melanin excretion. By switching the peptone amounts in YPD and MEA, we demonstrated that *E. viscosa* JF 03-3F acquired the ability to excrete melanin on MEA if it was supplemented with 2% peptone (Supplementary Fig. 12). To extend this observation, we added progressively higher amounts of peptone to MEA media (i.e. 10 different concentrations of peptone ranging from 0.1 to 5%). We observed that *E. viscosa* JF 03-3F starts excreting melanin on MEA at a peptone amount of 2%, and *E. viscosa* JF 03-4F starts excreting melanin at about 4% peptone (Fig. 14).

To determine if it was tyrosine (the precursor to L-DOPA and HGA/tyrosine-derived melanins) in the peptone that was inducing melanin excretion at higher peptone levels, we tested differing tyrosine amounts on our fungi. When 0.5 g/L of tyrosine is added to the media, *E. viscosa* JF 03-3F and *E. viscosa* JF 03-4F are capable of melanin excretion, whereas increasing the tyrosine amount to 1 g/L melanin excretion is stopped completely in *E. viscosa* JF 03-4F and is greatly reduced in *E. viscosa* JF 03-3F. Additionally, when tyrosine is the only source of nitrogen at 0.5 g/L, no melanin excretion is observed in the WT strains (Supplementary Fig. 13). Additional tests of added amino acids and ammonium chloride's effect on melanin excretion were also performed. A summarized description of the media variants and their results on these fungi's ability to excrete melanin can be found in Supplementary Table 3. Additional details and further understanding of these results are to be described at a later date.

#### Confirmation of melanin excretion from living cells and determining its OD amount in the supernatant

To confirm that the dark pigment in culture supernatants is indeed melanin, we performed a melanin extraction using previously described methods (Pal *et al.* 2014; Pralea *et al.* 2019). Although both enzymatic and chemical extraction of melanin was attempted, the chemical extraction process proved to be more efficient at recovering excreted melanin from the supernatant (Supplementary Fig. 14). Therefore, all extractions going forward were performed using the chemical extraction method to ensure all melanin was extracted from the solution.

**Fig. 15.**
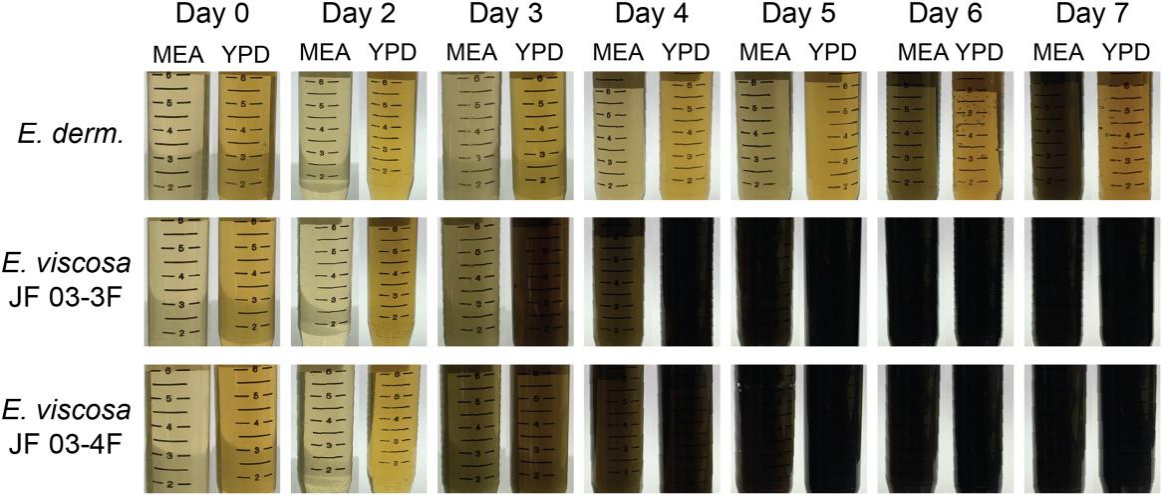
Supernatants of *E. dermatitidis* (*E. derm*.), *E. viscosa* JF 03-3F, and *E. viscosa* JF 03-4F for each day to observe their accumulation of external melanin. Melanin began to accumulate in the supernatant of *E. viscosa* JF 03-3F and *E. viscosa* JF 03-4F on day 3, with a steep increase in melanin in YPD. *E. dermatitidis* began melanin secretion on day 6, and only in MEA.

**Fig. 16.**
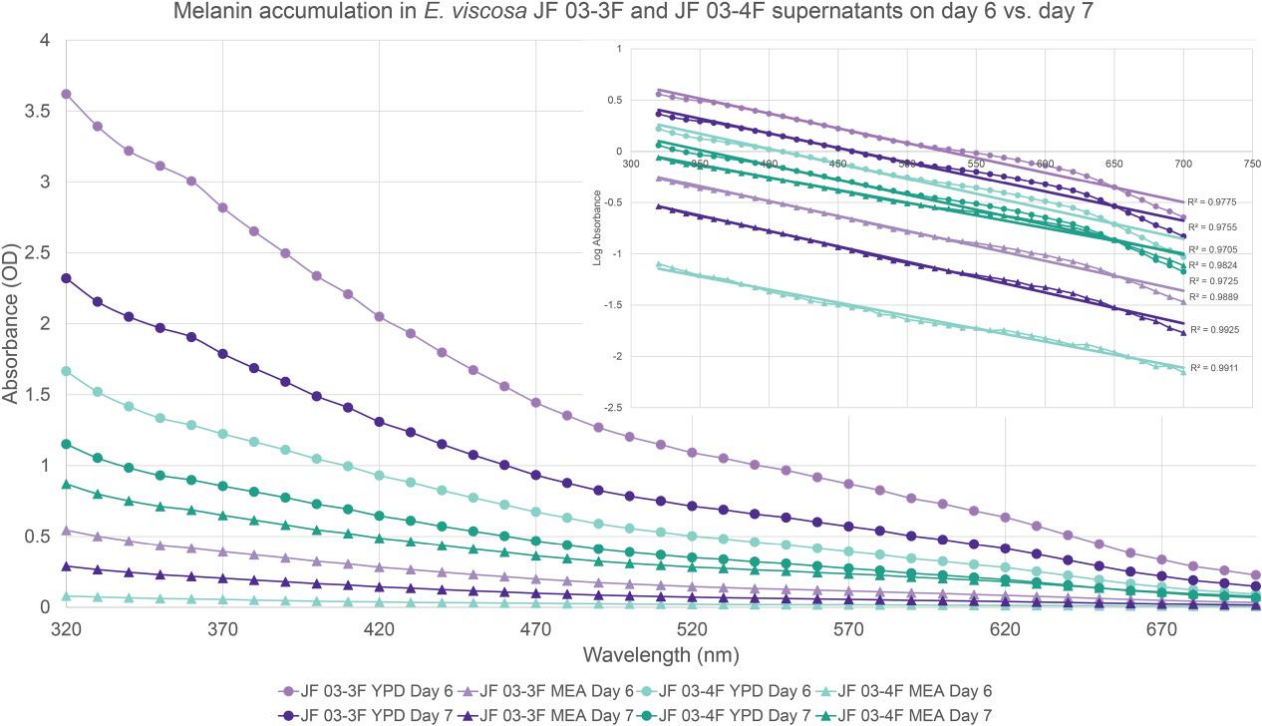
Days 6 and 7 results of daily melanin extraction from *E. viscosa* JF 03-3F and *E. viscosa* JF 03-4F. Both samples display typical melanin properties with full spectrum light, in that all samples have a linear regression when their Log Absorbance is plotted against wavelength, with an *R*^2^ value of 0.97 or higher (Pralea *et al*. 2019). The sample with the highest amount of secreted melanin was *E. viscosa* JF 03-3F in YPD on day 6. Both *E. viscosa* JF 03-3F and *E. viscosa* JF 03-4F had more secreted melanin when grown on YPD as opposed to MEA which showed lower melanin secretion for both species. Interestingly, both species had a decrease in melanin on day 7 when grown on YPD.

Growth of *E. viscosa* JF 03-3F*, E. viscosa* JF 03-4F, and *E. dermatitidis* on YPD and MEA were observed daily for melanin excretion; 11 mL aliquots of their supernatant were removed daily, and the melanin was extracted from each sample. As the experiment progressed, it was obvious that *E. viscosa* JF 03-3F and *E. viscosa* JF 03-4F began excreting melanin on day 3 and excreted more in YPD than in MEA (Fig. 15). *E. dermatitidis*, on the other hand, seemed to have a slower buildup of melanin, and melanin wasn’t truly obvious in the medium until day 6 in MEA though it can be seen in MEA day 3 in Fig. 15. Analysis of the OD of the final extracted melanin revealed that melanin amount increased over the course of the experiment and that greater amounts of melanin are released in YPD for *E. viscosa* JF 03-3F and *E. viscosa* JF 03-4F compared to MEA for *E. dermatitidis* (Supplementary Figs. 13–15). Interestingly, for both *E. viscosa* JF 03-3F and *E. viscosa* JF 03-4F, their peak melanin excretion was on day 6 and not on day 7 when grown in YPD (Fig. 16). There was less melanin on day 7 for both strains, indicating that the melanin after day 6 was either degrading or was being taken back up by the cells. This was only obvious by reading the OD results of the extracted melanin, as the supernatants themselves past day 5 were all strikingly dark and hard to differentiate visually (Fig. 15). Extracted melanin from all *E. viscosa* JF 03-3F, *E. viscosa* JF 03-4F, and *E. dermatitidis* samples (except *E. dermatitidis* in MEA on day 3) displayed the typical results from a full spectrum read on pure melanin (Supplementary Figs. 15–17; Table 2). When graphed, absorbance to Wavelength, the sample should have an exponentially decreasing OD as wavelength increases, and when the log10 of the OD absorbance reads is calculated, the full spectrum reads should become a linear regression with an R^2^ value of 0.97 or higher (Pralea *et al.* 2019). All *E. viscosa* JF 03-3F*, E. viscosa* JF 03-4F, and *E. dermatitidis* supernatant samples displayed these features (Supplementary Table 2), and therefore, we can confirm that the dark nature of their supernatants is caused by excreted melanin.

### Genetic analysis of melanin production

#### Creation of an albino mutant using chemical mutagenesis

**Fig. 17.**
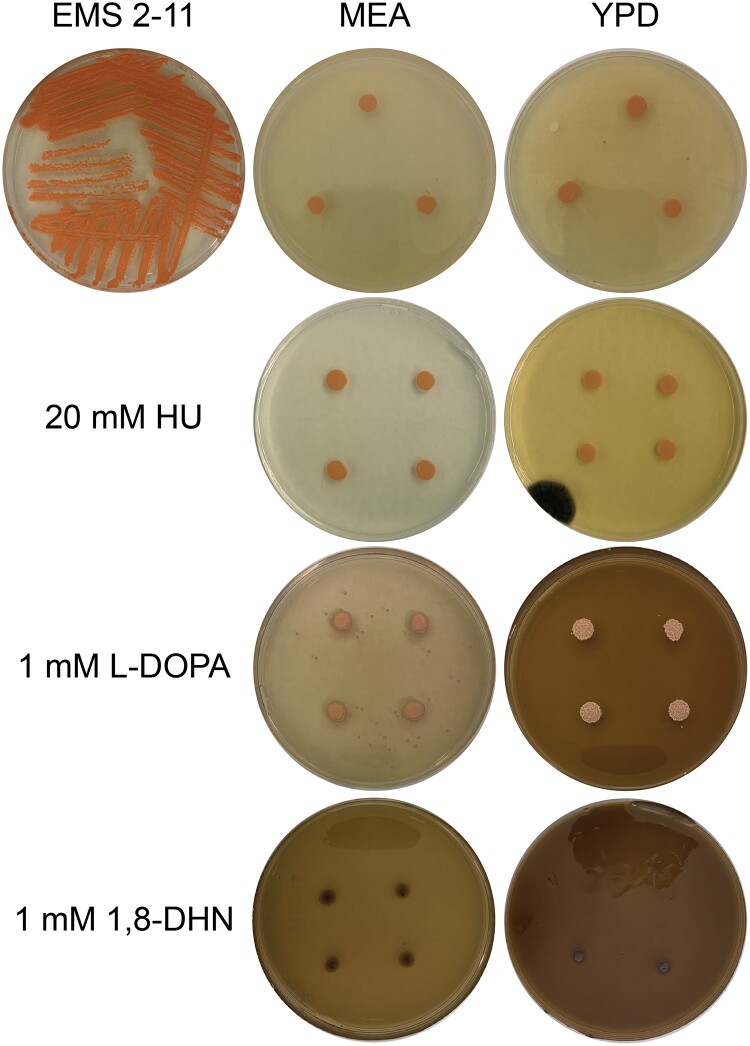
Phenotype of the EMS 2-11 mutant of *E. viscosa* JF 03-4F with *pks1* nonsense mutation Q814STOP, causing melanin production to be stopped hence the pink coloration. Attempts to recover melanin production were done with HU, L-DOPA, and 1,8-DHN. Neither HU nor L-DOPA were able to recover melanin production in the mutant, however, 1,8-DHN was able to recover melanin production in this mutant.

At this time, molecular tools for the manipulation of *E. viscosa* JF 03-3F and *E. viscosa* JF 03-4F are not yet available. Therefore, we combined classical genetics with genome re-sequencing to initially investigate the regulation of melanin production. Following mutagenesis with the alkylating agent ethyl methanesulfonate (EMS), 4 distinct phenotypic classes of mutants were recovered: pink/albino, crusty, brown, and melanin over-excretion (data not shown). At this time, the most important of these phenotypes was the pink/albino phenotype found in a mutant of *E. viscosa* JF 03-4F, a mutant we named EMS 2-11 (Fig. 17). Genome re-sequencing of high molecular weight DNA from mutant EMS 2-11 and comparison to the reference *E. viscosa* JF 03-4F genome revealed a deleterious SNP causing a nonsense mutation in the gene *pks1*. This mutation was on position 2,346,590 in scaffold 3, located in the *pks1* gene, which caused a C -> T mutation on the first position of the codon such that it became a stop codon, Q814STOP. Interestingly, *pks1* only functions in one of the 3 melanin biosynthetic pathways found in *E. viscosa* JF 03-4F, specifically the DHN/allomelanin pathway, which is typically known as the main melanin produced by polyextremotolerant fungi. Only 2 of the other random mutations found in EMS 2-11 were missense or nonsense mutations. One is a mutation in a transcriptional repressor EZH1, and another is in alcohol dehydrogenase GroES-like/PKS enoylreductase. Although either of these mutations could theoretically contribute to the pink phenotype, it is more likely that a nonsense mutation in *pks1* is solely responsible for the loss of melanin production despite the lack of mutations in the other melanin biosynthetic pathways. This mutant is considered albino but is colored pink because of carotenoid production (Noack-Schönmann *et al.* 2014; Tesei *et al.* 2017). It has already been shown that blockage of melanin production in *E. dermatitidis* by mutations in *pks1* results in pink instead of white albino colonies because they also produce carotenoids and byproducts such as flavolin that are normally masked by melanin (Geis *et al.* 1984; Geis and Szaniszlo 1984).

#### Recovery of melanin production in an albino mutant: modern use of the classical beadle and tatum biochemical-genetics experiment

Pks1 is a PKS that is essential for the first step in the DHN-melanin/allomelanin production pathway. However, both *E. viscosa* JF 03-4F and *E. viscosa* JF 03-3F are shown to have the genetic capabilities to produce all 3 forms of fungal melanins, which means theoretically any of the 3 pathways could be activated to produce melanin if the fungi are given the proper chemical precursors. To test if “activation” of any of the 3 melanin production pathways could restore melanin production to the EMS 2-11 mutant, we performed the classical Beadle and Tatum biochemical-genetics experiment (Beadle and Tatum 1941) and substituted either 1 mM of L-DOPA, 20 mM HU, tyrosine (additive of 0.5 g/L, 1 g/L, or 0.5 g/L sole nitrogen source), or 1 mM of 1,8-DHN into the medium. L-DOPA was previously shown to recover melanization in a *pks1* deletion mutant of *E. dermatitidis*, thus we presumed that it would be taken up by our EMS 2-11 mutant and activate the DOPA-melanin/pheomelanin biosynthesis pathway (Paolo *et al.* 2006; Dadachova *et al.* 2007). However, the substitution of 1 mM L-DOPA into either MEA or YPD did not induce melanin production in the EMS 2-11 *pks1* mutant (Fig. 17). These plates were grown in the dark for 10 days, as L-DOPA will auto polymerize into a melanin precursor in the presence of light, and still no melanin production was observed.

The addition of tyrosine, a melanin precursor molecule for L-DOPA and HGA-based melanins, also did not recover EMS 2-11's melanin production in any of the media iterations tested (Supplementary Fig. 13). HU is another compound that in other *Exophiala* species induced melanin production (Schultzhaus *et al.* 2020), however, the addition of 20 mM of HU also did not activate melanin production in our EMS 2-11 albino mutant (Fig. 17). Finally, addition to the media of 1 mM of 1,8-DHN, which is the immediate precursor to the final DHN-melanin/allomelanin molecule and is downstream of *pks1*, did result in recovery of melanized colonies. The colonies that grew on MEA+ 1 mM 1,8-DHN did not form extensive growth, but their cells were still dark, and on YPD + 1,8-DHN active dark colony growth is observed (Fig. 17). These results suggest that the DHN-melanin produced by the Pks1 PKS is responsible for the bulk of melanin production by *E. viscosa* JF 03-4F.

## Discussion

A major limitation to our understanding of the roles played by BSCs in semi-arid ecosystems is our lack of insight into the microbial components of these biofilms and the nature of their functional interactions (Carr *et al.* 2021). As photoautotrophs, the roles of cyanobacteria and algae in BSCs are clear (Belnap 2002), but the importance of other residents such as fungi and heterotrophic bacteria is less so. Here, we identify 2 isolates of a novel species of black yeasts from BSCs; *E. viscosa* 2 strains JF 03-3F and JF 03-4F. In addition to presenting the complete annotated genome sequence for both strains, we also provide a relatively detailed profile of their ability to utilize diverse carbon and nitrogen sources, as well as their response to different forms of stress. Most importantly, we demonstrate that *E. viscosa* JF 03-3F and *E. viscosa* JF 03-4F are capable of producing excessive amounts of melanin, including melanin that is excreted at high levels. Our results suggest that by making melanin extracellularly available as a public good, polyextremotolerant fungi provide a critical service to the broader BSC community.

### *E. viscosa* and its closest relatives

*E. viscosa* JF 03-3F and *E. viscosa* JF 03-4F are 2 strains of a novel fungus from the family Herpotrichielleaceae that we isolated from a lichen-dominated BSC. Distinguishing features of these isolates include their yeast-dominated morphology, pronounced melanization, and production of extracellular material that leads to the formation of “goopy” or “slimy” colonies. Notably, numerous other melanized fungi with filamentous or polymorphic characteristics were also isolated from the same BSC communities. These fungi, which will be described in detail elsewhere, grow much more slowly than *E. viscosa* JF 03-3F or *E. viscosa* JF 03-4F and also release much less or no melanin.

Phylogenetic comparisons suggest that the closest relatives of *E. viscosa* JF 03-3F and *E. viscosa* JF 03-4F are *E. sideris*, *E. bergeri*, *P. elegans*, and *E*. *nigra*. However, through extensive phylogenetic analyses, we can confirm that *E. viscosa* is a distinct species separate from these related fungi. A comparison of the 2 strains respective annotated genome sequences revealed that overall gene content and characteristics are similar to *E. sideris*. *E*. *sideris* belongs to the *jeanselmei*-clade of *Exophiala*; it is typically recovered from highly arsenate-polluted environments and is not known to be pathogenic (Seyedmousavi *et al.* 2011). The failure of *E. viscosa* JF 03-3F and *E. viscosa* JF 03-4F to grow at 37°C reinforces the point that species within this clade are less likely to possess the capacity of causing disease in mammals.

The organization of the *MAT* locus between both strains of *E. viscosa* and *E. sideris* is very similar, as they possess a fused *MAT* gene, although they differ in the transcript directionality for the flanking *COX13* and *APC5* genes. Many *Exophiala* species are considered heterothallic, even species closely related to *E. sideris* such as *E. oligosperma* and *E. spinifera* are heterothallic (Untereiner and Naveau 1999; Teixeira *et al.* 2017). As previously proposed by Teixeira *et al.*, the proposed homothallic behavior of *E. sideris*, *E. viscosa* JF 03-3F, and *E. viscosa* JF 03-4F, may have resulted from an ancestral parasexual recombination event that altered the organization of the *MAT* locus (2017). Further investigation of these ideas will require demonstration that these *Exophiala* species undergo sexual development. Additional investigations will also be performed to determine the environmental factors for triggering sexual or asexual development in *E. viscosa*, as past attempts of established methods have failed but do not constitute evidence for the absence of development.

Phenotypic differences between the 2 strains of *E. viscosa* were unable to be elucidated using their automatic gene annotations. However, their gene synteny dot plots could indicate locations of the genome where gene synteny has influenced their phenotype. The dot plot comparing the 2 *E. viscosa* strains showed high % sequence similarity, but very distinct regions of nonsyntenic genes. If the genes responsible for cell polarity are found within the nonsyntenic regions, then it could be a reason for the morphological differences we have observed. However, we were also able to see gene transcription direction differences between the strains in some genes, including the gene COX13 of the *MAT* locus which is forward in the strain JF 03-3F and reverse in JF 03-4F. Between the differences in gene synteny and transcriptional direction, there could be a reason to re-evaluate the intraspecific variation of these strains once it becomes standard practice for fungal taxonomic naming systems to compare their whole genomes, as is done with bacteria.

### Phenotypic characterization of *E. viscosa* JF 03-3F and *E. viscosa* JF 03-4F

Our results show that *E. viscosa* JF 03-3F and *E. viscosa* JF 03-4F are capable of using a diverse array of carbon sources. These include mannitol, ribitol, and glucose, which are considered the main carbon sources exchanged between photobionts and their mycobiont partners in lichens (Richardson *et al.* 1967; Yoshino *et al.* 2020). Although, *E. viscosa* JF 03-3F and *E. viscosa* JF 03-4F are able to use most nitrogen sources provided, glutamate and aspartate are clearly not being taken up or used, unlike in yeasts such as *S. cerevisiae* where glutamate is more favorable nitrogen source used for multiple core metabolism and amino acid production processes (Ljungdahl and Daignan-Fornier 2012). Because peptone is a favorable nitrogen source for *E. viscosa* JF 03-3F and *E. viscosa* JF 03-4F, it is likely that this species can import peptides and degrade them as organic nitrogen sources. In the BSC soil environment, organic nitrogen has been shown to be the more abundant form of nitrogen (Delgado-Baquerizo *et al.* 2010), but whether cyanobacteria and other nitrogen-fixing bacteria in BSCs are contributing organic or inorganic nitrogen sources is not understood (Belnap 2002). However, since these fungi can use both inorganic and organic forms of nitrogen, they can use their metabolic flexibility to benefit them when either is present.

A characteristic feature of BSCs is the inherently stressful nature of these ecosystems. Of note, *E. viscosa* JF 03-3F and *E. viscosa* JF 03-4F were isolated from a site that can experience extremes in temperature, UV exposure, and soil wetness/osmotic pressure. Although the optimal growth range for these strains is from 15°C to 27°C, they are capable of slow growth at 4°C and while they do not grow at 37°C, they do remain viable after 72 consecutive hours of exposure to this temperature. The ability to grow at 4°C, albeit slowly, presumably contributes to the success of fungi such *E. viscosa* JF 03-3F and *E. viscosa* JF 03-4F in adapting to BSCs in colder drylands. A broader comparison of microbiomes and associated functional traits between cold-adapted BSCs and those found in hot deserts would be an informative approach to identifying microbial species and/or physiological factors that underlie temperature adaptation.

As for resistance to UV radiation, *E. viscosa* JF 03-3F and *E. viscosa* JF 03-4F were both able to survive the high exposure to UV-C and were uniquely resistant to standard UV mutagenesis conditions. Potential mechanisms that might contribute to UV resistance include the presence of melanin or other defensive compounds (i.e. carotenoids), some of which could be public goods provided by other constituents of the BSC community, or an enhanced capacity to repair damaged DNA. This will ultimately be an interesting topic to investigate in the future.

One feature we expected to find in *E. viscosa* JF 03-3F and *E. viscosa* JF 03-4F, but we did not necessarily observe was higher metal resistance. Melanin is known to enhance resistances to metals (Bell and Wheeler 1986; Gadd 1994; Purvis *et al.* 2004; Gorbushina 2007), and their closest relative *E. sideris* is primarily isolated from arsenate-contaminated soils (Seyedmousavi *et al.* 2011), so we predicted that this species would have increased metal tolerance when compared to nonmelanized fungi. However, of the metals tested, only AgNO_3_ and CuCl_2_ showed a decreased zone of clearing (higher resistance) in *E. viscosa* JF 03-3F and *E. viscosa* JF 03-4F, compared to *S. cerevisiae* and *E. dermatitidis*. A potential explanation for the latter observation is that melanin has a higher affinity for copper due to the phenolic compounds within the polymer (Gadd and de Rome 1988).

### Production and excretion of melanin by *E. viscosa* JF 03-3F and *E. viscosa* JF 03-4F

Melanin is considered an expensive secondary metabolite to produce because it is made up of conglomerates of polyphenols and as such contains many carbons (Schroeder *et al.* 2020). Therefore, it would be assumed that an organism would not want to create melanin just to export it out of the cell. Either this excreted melanin is an evolutionary trait that is beneficial to the fungi in some way, or this melanin excretion is a form of overflow metabolism in which melanin is the most streamlined way to remove an excessive buildup of precursors (De Deken 1966). Additionally, there are not many fungi known to excrete melanin, and few in the genus *Exophiala* (Romsdahl *et al.* 2021), so the possibility of a novel species that excrete such extreme amounts of melanin into their surroundings was intriguing.

Both fungi have the genetic make-up to produce all 3 types of fungal melanin: allomelanin (DHN-derived), pyomelanin (HGA/L-tyrosine-derived), and pheomelanin (L-DOPA-derived); but it is still unknown how these melanin biosynthetic pathways are regulated. We were able to confirm through genetic means that they are producing DHN-derived allomelanin, but we were unable to definitively confirm the production of the other 2 forms of melanin. Additionally, we can speculate that tyrosine-derived melanins are not being directly produced for excretion under conditions we have tested, since using tyrosine as a sole nitrogen source did not induce melanin excretion. However, tyrosine used as an additive did induce melanin excretion at 0.5 g/L but not at 1 g/L, implying a possible tyrosine-dependent regulation of melanin production/excretion that is not entirely understood. We hope to eventually elucidate the melanin(s) that are produced by these fungi by combining genetic approaches with mass spectrometry and NMR in the future.

In our attempts to understand the regulation of melanin production by *E. viscosa* JF 03-3F and *E. viscosa* JF 03-4F, we tried blocking individual pathways using known chemical blockers of DHN and L-DOPA-melanin, phthalide, and kojic acid, respectively. Neither blocker nor their combination at the highest amounts used was able to block the production of melanin in either strain. If this species has the capability to produce all 3 melanins, then it is possible that they are producing more than one at the same time. This could be the case here since the combination of both kojic acid and phthalide still resulted in melanized cells which would indicate that tyrosine-derived pyomelanin was the cause of melanization. Alternatively, since these fungi are fully melanized, it could be that the concentrations used on fungi that use melanin in asexual structures only were not strong enough to block melanin production in these fungi. Additionally, our melanin gene annotation revealed that both fungi contain homologs to *A. niger* and *A. fumigatus’* Arp1 and Arp2, which could be causing a bifurcation of the DHN-melanin pathway and prevent phthalide from being an effective blocker of melanin production (Wheeler and Klich 1995; Jørgensen *et al.* 2011).

Linked regulation of potentially multiple forms of melanin, was observed with an EMS-induced mutant in which the *pks1* gene was disrupted. Although *pks1* is the first enzyme involved in only the production of DHN-derived allomelanin, the cells that resulted from this mutation were albino. Theoretically, these cells should have still been capable of producing the 2 other melanins as their pathways were seemingly not disrupted by any mutations, but this was not the case. The addition of L-DOPA has been shown to induce melanization in a *pks1* deletion mutant of *E. dermatitidis* (Paolo *et al.* 2006), which would indicate that it is capable of producing both pheomelanin and allomelanin. However, the same experiment did not restore melanization to the *E. viscosa* JF 03-4F's *pks1* nonsense mutant. Therefore, it is possible that; (i) *E. viscosa* JF 03-3F and *E. viscosa* JF 03-4F only produce DHN-derived allomelanin and disruption of *pks1* causes all melanin production to shut down, (ii) *pks1* (or downstream creation of DHN-derived allomelanin) is essential for the regulation of other melanin productions, or (iii) pheomelanin and pyomelanin are only produced under specific conditions that were not tested during analysis of the mutants.

Our experiments also showed that *E. viscosa* JF 03-3F and *E. viscosa* JF 03-4F actively excrete melanin when they are viable and growing. We observed that melanin began to accumulate starting as early as day 2 (by OD reads), therefore, suggesting live cells are excreting melanin and not melanin coming out of dead lysed cells. However, at this time we do not have a mechanism for how melanin is being released from the cells and something to be investigated later. We also observed a decrease in melanin concentrations after day 6 in both YPD and MEA, which raises the intriguing potential for melanin uptake or degradation by *E. viscosa* JF 03-3F and *E. viscosa* JF 03-4F. Additionally, we observed that increasing organic nitrogen in the media-induced melanin excretion from both fungi, but we do not know how this phenomenon is regulated or which melanin is being excreted.

### Insights into the niche of polyextremotolerant fungi within the BSC consortium

Fungal residents of BSC communities include lichenized mycobionts, lichenicolous fungi, endolichenic fungi, and free-living fungi. Although the latter is likely not directly associated with lichens themselves, they are surrounded by algae, cyanobacteria, and other bacteria in the same way that lichen-associated fungi are surrounded by a similar community of microbes. This raises the intriguing possibility that fungi such as *E. viscosa* JF 03-3F and *E. viscosa* JF 03-4F might be transiently associated with autotrophs in response to specific environmental conditions. Members of the genus *Exophiala* (Class Eurotiomycetes) are phylogenetically related to the lichen-forming Verrucariales and reside between Lecanoromycetes and Lichinomycetes (James *et al.* 2006). Additionally, the lifestyle of the ancestral state of Eurotiomycetes is currently debated as to whether it is lichenized or not. Originally, it was thought that the ancestral state of Eurotiomycetidae was lichenized (Lutzoni *et al.* 2001), but recent literature has shown that the ancestral state was more likely nonlichenized (Lücking and Nelsen 2018; Nelsen *et al.* 2020). However, many have recognized the similarities between taxa residing in Eurotiomycetes that are nonlichenized to their lichenized relatives. Particularly, members of Eurotiomycetes and Lecanoromycetes share a commonality in their ecological locations (Gostinčar *et al.* 2012; Muggia *et al.* 2013) and in their type 1 PKS genes (Boustie and Grube 2005), which are thought to be acquired by lichen-forming fungi via horizontal gene transfer from actinobacteria (Schmitt and Lumbsch 2009). As such, it is likely that *E. viscosa* JF 03-3F and *E. viscosa* JF 03-4F, and other related fungi have lichen-like qualities, which allow them to interact with algae, cyanobacteria, and other bacteria to form lichen-like symbioses. The idea that polyextremotolerant fungi can engage in lichen-like associations with microbes has been postulated (Gostinčar *et al.* 2012; Muggia *et al.* 2013), but direct experimentation is still needed to confirm these ideas. As these are 2 strains of a newly identified species, a more extensive ecological and molecular investigation is needed to determine the extent to which this taxon is found in BSCs and to elucidate their species interaction networks.

Correlation between environmental organic nitrogen availability and excreted melanin amounts could have strong implications for interactions between these fungi found in BSCs and their nitrogen-fixing community partners. The presumed source of nitrogen used to enhance the excretion of melanin would be nitrogen-fixing prokaryotes such as cyanobacteria, other bacteria, and archaea in the community. Additionally, organic nitrogen was found to be more abundant than inorganic nitrogen in BSC soils, and more abundant than in other soil ecosystems (Delgado-Baquerizo *et al.* 2010). However, the nitrogen source that is released into the environment by nitrogen-fixing bacteria in BSCs is not known. It could be ammonia/ammonium, or it could be an organic form of nitrogen since cyanobacteria and diazotrophs are known to release both forms of nitrogen (Vitousek *et al.* 2002; Berthelot *et al.* 2015; Gimenez *et al.* 2016). Our results suggest that the availability of these nitrogen-fixers to produce a pool of organic nitrogen could subsequently induce the excretion of melanin which then provides protective services to the broader BSC community. Excreted melanin could function at the community-level to maintain respiration and growth under varying temperature conditions while also mitigating the effects of desiccation and exposure to UV, extending the window of time allotted for optimal photosynthetic capabilities (Lange and Tenhunen 1981; Honegger 1997; Honegger and Haisch 2001; Nybakken *et al.* 2004; Carr *et al.* 2021). Moreover, the excreted melanin could simultaneously serve as an external source of carbon used by the fungi and possibly others when the environment does not allow for photosynthesis. In either scenario, the seemingly wasteful excretion of melanin by *E. viscosa* JF 03-3F and *E. viscosa* JF 03-4F instead represents the optimal sharing of an expensive public good for the overall betterment of the BSC community in which they reside.

The availability of complete, annotated genome sequences for *E. viscosa* JF 03-3F and *E. viscosa* JF 03-4F will enable more detailed investigations of the mechanisms that support the adaptation of these fungi to extreme environments. This in turn should provide greater insight into the roles that these fungi play in the BSC community. Of particular interest will be the regulatory mechanisms that coordinate the production of different types of melanin in response to environmental and chemical inputs. Moreover, the broad applicability of melanin as a bioproduct has triggered growing interest in microorganisms that excrete this polymer. In this context, *E. viscosa* JF 03-3F and *E. viscosa* JF 03-4F could potentially serve as excellent platforms for additional engineering focused on optimizing or tailoring the type of melanin produced in responses to specific applications.

## Supplementary Material

jkad110_Supplementary_Data

## Data Availability

All data generated is available through the JGI Mycocosm website under *E. viscosa* JF 03-3F and *E. viscosa* JF 03-4F, or at NCBI under accession numbers JALDXI000000000 for *E. viscosa* JF 03-3F and JALDXH000000000 for *E. viscosa* JF 03-4F. Transcriptomes for these fungi can be found at NCBI using the SRA numbers: SRX5076261 for *E. viscosa* JF 03-3F, and SRX5077344 for *E. viscosa* JF 03-4F. All accession numbers from all databases for both strains are listed in Table 5. The strains of *E. viscosa* JF 03-3F CBS 148801 and *E. viscosa* JF 03-4F CBS 148802 have also been deposited to the Westerdijk institute in a metabolically inactive state for access to the scientific community, but all strains used in this study can also be obtained from the corresponding author upon request. Finally, if any additional details or questions are required for the replication of any experiments listed above, one may reach out to the corresponding author. Supplemental Material available at figshare: https://doi.org/10.25387/g3.22687252.
