# Supplementary material for: Characterization of a novel polyextremotolerant fungus, Exophiala viscosa, with insights into its melanin regulation and ecological niche
Source: G3 (Bethesda). 2023 May 23;13(8):jkad110. doi: 10.1093/g3journal/jkad110 (PMC10411609; doi:10.1093/g3journal/jkad110)

ITS-only ASAP analysis:

Partition 1
Asap-Score: 1.000000
P-value: 2.814371e-01
Threshold distance: 1.32%
Nb subsets with recursion:18 (without recursion: 17)

Subset[1] n: 2 ;id: Exophiala_viscosa_JF_03-4F Exophiala_viscosa_JF_03-3F
Subset[2] n: 1 ;id: Exophiala_nigra
Subset[3] n: 1 ;id: Exophiala_spinifera
Subset[4] n: 1 ;id: Exophiala_bergeri
Subset[5] n: 1 ;id: Exophiala_dermatitidis
Subset[6] n: 2 ;id: Exophiala_sideris Phaeoannellomyces_elegans
Subset[7] n: 1 ;id: Exophiala_lecanii-corni
Subset[8] n: 1 ;id: Exophiala_cancerae
Subset[9] n: 1 ;id: Exophiala_castellanii
Subset[10] n: 1 ;id: Exophiala_crusticola
Subset[11] n: 1 ;id: Exophiala_salmonis
Subset[12] n: 1 ;id: Exophiala_nishimurae
Subset[13] n: 1 ;id: Exophiala_pisciphila
Subset[14] n: 1 ;id: Exophiala_aquamarina
Subset[15] n: 1 ;id: Exophiala_xenobiotica
Subset[16] n: 1 ;id: Exophiala_oligosperma
Subset[17] n: 1 ;id: Exophiala_jeanselmei
Subset[18] n: 1 ;id: Endocarpon_pusillum

Nb of subsets	asap-score		P-val (rank)	W (rank)	Treshold dist.	Text
* 18	1.00		2.81e-01 (1)	9.71e-03 (1)	0.013177	list csv
8	4.00		6.45e-01 (3)	5.90e-03 (5)	0.109561	list csv
* 16	4.00		9.12e-01 (4)	5.93e-03 (4)	0.041445	list csv
* 17	4.50		6.37e-01 (2)	5.27e-03 (7)	0.028664	list csv
13	8.50		9.58e-01 (6)	2.90e-03 (11)	0.066657	list csv
11	8.50		9.62e-01 (7)	2.98e-03 (10)	0.080038	list csv
9	10.00		1.00e+00 (14)	5.77e-03 (6)	0.100786	list csv
7	10.50		9.64e-01 (8)	2.46e-03 (13)	0.124177	list csv
20	10.50		1.00e+00 (19)	9.36e-03 (2)	0.000000	list csv
19	10.50		1.00e+00 (18)	8.31e-03 (3)	0.004722	list csv

Histogram of distances [save]

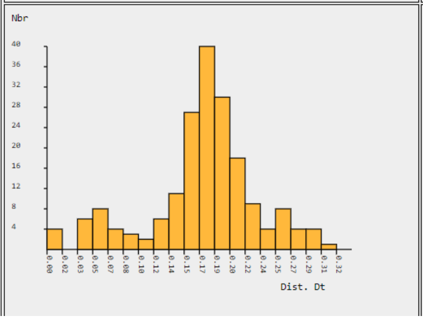

Ranked distances [save]

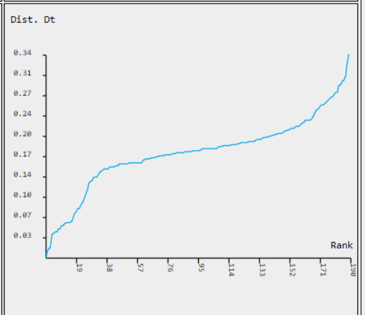

18S-28S-ITS concatenation ASAP analysis:

Partition 1
Asap-Score: 2.000000
P-value: 1.477046e-01
Threshold distance: 0.75%
Nb subsets with recursion:18 (without recursion: 17)

Subset[1] n: 2 ;id: Exophiala viscosa JF 03-3F Exophiala viscosa JF 03-4F
Subset[2] n: 1 ;id: Exophiala nigra
Subset[3] n: 2 ;id: Exophiala sideris Phaeoannellomyces elegans
Subset[4] n: 1 ;id: Exophiala bergeri
Subset[5] n: 1 ;id: Exophiala spinifera
Subset[6] n: 1 ;id: Exophiala aquamarina
Subset[7] n: 1 ;id: Exophiala cancerae
Subset[8] n: 1 ;id: Exophiala castellanii
Subset[9] n: 1 ;id: Exophiala dermatitidis
Subset[10] n: 1 ;id: Exophiala jeanselmei
Subset[11] n: 1 ;id: Exophiala lecanii-corni
Subset[12] n: 1 ;id: Exophiala nishimurae
Subset[13] n: 1 ;id: Exophiala oligosperma
Subset[14] n: 1 ;id: Exophiala pisciphila
Subset[15] n: 1 ;id: Exophiala salmonis
Subset[16] n: 1 ;id: Exophiala xenobiotica
Subset[17] n: 1 ;id: Exophiala crusticola
Subset[18] n: 1 ;id: Endocarpon pusillum

Nb of subsets	asap-score	P-val (rank)		W (rank)	Treshold dist.	Text
* 18	2.00		1.48e-01 (1)	2.89e-03 (3)	0.007509	list csv
* 11	4.00		7.92e-01 (2)	1.91e-03 (6)	0.029425	list csv
2	4.50		9.30e-01 (5)	1.94e-03 (4)	0.069412	list csv
* 9	7.50		9.76e-01 (7)	1.74e-03 (8)	0.030263	list csv
* 7	8.00		9.74e-01 (6)	1.07e-03 (10)	0.033474	list csv
* 16	8.50		8.86e-01 (4)	7.46e-04 (13)	0.011888	list csv
* 6	9.50		8.80e-01 (3)	4.85e-04 (16)	0.038156	list csv
20	10.00		1.00e+00 (19)	6.35e-03 (1)	0.000000	list csv
19	10.00		1.00e+00 (18)	4.28e-03 (2)	0.003195	list csv
* 10	10.00		1.00e+00 (15)	1.91e-03 (5)	0.029802	list csv

Histogram of distances [save]

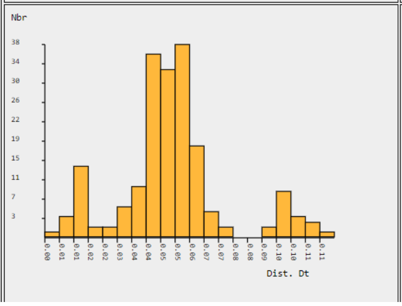

Ranked distances [save]

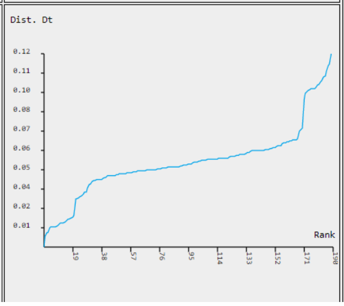

18S-28S-ITS-rpb1 concatenation ASAP analysis:

Partition 1
Asap-Score: 2.500000
P-value: 9.321357e-01
Threshold distance: 2.56%
Nb subsets with recursion:16 (without recursion: 15)

Subset[1] n: 2 ;id: Exophiala viscosa JF 03-3F Exophiala viscosa JF 03-4F
Subset[2] n: 1 ;id: Exophiala sideris
Subset[3] n: 1 ;id: Exophiala bergeri
Subset[4] n: 1 ;id: Exophiala nigra
Subset[5] n: 1 ;id: Exophiala spinifera
Subset[6] n: 2 ;id: Exophiala aquamarina Exophiala pisciphila
Subset[7] n: 1 ;id: Exophiala salmonis
Subset[8] n: 1 ;id: Exophiala cancerae
Subset[9] n: 1 ;id: Exophiala castellanii
Subset[10] n: 1 ;id: Exophiala dermatitidis
Subset[11] n: 1 ;id: Exophiala jeanselmei
Subset[12] n: 1 ;id: Exophiala lecanii-corni
Subset[13] n: 1 ;id: Exophiala nishimurae
Subset[14] n: 1 ;id: Exophiala oligosperma
Subset[15] n: 1 ;id: Exophiala xenobiotica
Subset[16] n: 1 ;id: Endocarpon pusillum

Nb of subsets	asap-score		P-val (rank)	W (rank)	Treshold dist.	Text
* 16	2.50		9.32e-01 (2)	6.47e-03 (3)	0.025639	list csv
* 15	3.00		4.64e-02 (1)	2.19e-03 (5)	0.028228	list csv
* 14	3.50		9.40e-01 (3)	2.21e-03 (4)	0.031368	list csv
2	6.50		9.62e-01 (6)	1.86e-03 (7)	0.110115	list csv
* 11	7.50		9.42e-01 (4)	1.03e-03 (11)	0.040017	list csv
* 12	8.50		9.68e-01 (7)	1.08e-03 (10)	0.038969	list csv
18	9.00		1.00e+00 (17)	2.36e-02 (1)	0.000000	list csv
* 17	9.00		1.00e+00 (16)	1.32e-02 (2)	0.012071	list csv
7	9.50		1.00e+00 (13)	2.04e-03 (6)	0.051783	list csv
4	10.50		9.56e-01 (5)	5.97e-04 (16)	0.070077	list csv

Histogram of distances [save]

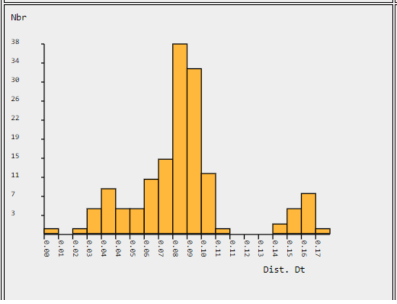

Ranked distances [save]

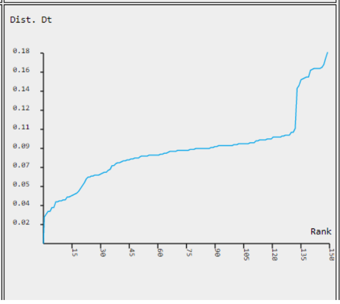

Supplement: jkad110_Supplementary_Data [file jkad110_supplementary_data.zip › File_S2.pdf]
