# Supplementary material for: Characterization of a novel polyextremotolerant fungus, Exophiala viscosa, with insights into its melanin regulation and ecological niche
Source: G3 (Bethesda). 2023 May 23;13(8):jkad110. doi: 10.1093/g3journal/jkad110 (PMC10411609; doi:10.1093/g3journal/jkad110)
Supplement: jkad110_Supplementary_Data [file jkad110_supplementary_data.zip › Supplemental_Tables.docx]

**Table S1:** R^2^ values for serially-diluted cell’s OD vs. labeled columns to determine best wavelength for OD measurements (Dilu. = dilution).

| **R^2^ Value** | **E. viscosa** JF 03-3F | | | | | **E. viscosa** JF 03-4F | | | | |
| --- | --- | --- | --- | --- | --- | --- | --- | --- | --- | --- |
| **λ** | **25% ≥ % Dilu.** | **50% ≥ % Dilu.** | **2.19x10^6 ≥ cells** | **4.38x10^6 ≥ cells** | **% Dilu. 12.5% ≥ accuracy** | **25% ≥ % Dilu.** | **50% ≥ % Dilu.** | **2.29x10^6 ≥ Cells** | **4.58x10^6 ≥ cells** | **% Dilu. 12.5% ≥**  **accuracy** |
| 300 | N/A | N/A | 0.9984 | N/A | 0.0046 | N/A | N/A | N/A | N/A | 0.0103 |
| 310 | N/A | N/A | 0.9984 | N/A | 0.0064 | N/A | N/A | N/A | N/A | 0.0174 |
| 320 | N/A | N/A | 0.9981 | N/A | 0.0187 | N/A | N/A | N/A | N/A | 0.0537 |
| 330 | N/A | N/A | 0.9978 | N/A | 0.0288 | 0.9802 | 0.8306 | 0.9802 | 0.8306 | 0.0168 |
| 340 | N/A | N/A | 0.9976 | N/A | 0.0172 | 0.9785 | 0.8483 | 0.9785 | 0.8483 | 0.0011 |
| 350 | N/A | N/A | 0.9973 | N/A | 0.0301 | 0.9776 | 0.8622 | 0.9776 | 0.8622 | 0.0205 |
| 360 | 0.9907 | 0.8266 | 0.9970 | 0.9907 | 0.0259 | 0.9781 | 0.8687 | 0.9781 | 0.8687 | 0.0260 |
| 370 | 0.9895 | 0.8443 | 0.9966 | 0.9895 | 0.0035 | 0.9773 | 0.8777 | 0.9773 | 0.8777 | 0.0677 |
| 380 | 0.9885 | 0.8606 | 0.9962 | 0.9885 | 0.0002 | 0.9770 | 0.8856 | 0.9770 | 0.8856 | 0.0817 |
| 390 | 0.9884 | 0.8741 | 0.9957 | 0.9884 | 0.0035 | 0.9764 | 0.8958 | 0.9764 | 0.8958 | 0.1075 |
| 400 | 0.9866 | 0.8911 | 0.9951 | 0.9866 | 0.0171 | 0.9742 | 0.9120 | 0.9742 | 0.9120 | 0.1447 |
| **410** | **0.9853** | **0.9027** | **0.9947** | **0.9853** | **0.0422** | **0.9722** | **0.9260** | **0.9722** | **0.9260** | **0.1633** |
| **420** | **0.9845** | **0.9149** | **0.9941** | **0.9845** | **0.0466** | **0.9693** | **0.9418** | **0.9693** | **0.9418** | **0.1848** |
| 430 | 0.9828 | 0.9264 | 0.9936 | 0.9828 | 0.0650 | 0.9673 | 0.9512 | 0.9673 | 0.9512 | 0.2192 |
| 440 | 0.9804 | 0.9410 | 0.9927 | 0.9804 | 0.1083 | 0.9653 | 0.9596 | 0.9653 | 0.9596 | 0.2243 |
| 450 | 0.9776 | 0.9545 | 0.9917 | 0.9776 | 0.1372 | 0.9638 | 0.9555 | 0.9638 | 0.9555 | 0.2141 |
| 460 | 0.9747 | 0.9653 | 0.9906 | 0.9747 | 0.1729 | 0.9623 | 0.9535 | 0.9623 | 0.9535 | 0.2215 |
| 470 | 0.9718 | 0.9624 | 0.9898 | 0.9718 | 0.1985 | 0.9615 | 0.9530 | 0.9615 | 0.9530 | 0.2177 |
| 480 | 0.9698 | 0.9572 | 0.9892 | 0.9698 | 0.2177 | 0.9607 | 0.9503 | 0.9607 | 0.9503 | 0.2340 |
| 490 | 0.9683 | 0.9539 | 0.9888 | 0.9683 | 0.2507 | 0.9601 | 0.9486 | 0.9601 | 0.9486 | 0.2310 |
| 500 | 0.9673 | 0.9516 | 0.9884 | 0.9673 | 0.2849 | 0.9600 | 0.9474 | 0.9600 | 0.9474 | 0.2116 |
| 510 | 0.9666 | 0.9410 | 0.9882 | 0.9666 | 0.3161 | 0.9597 | 0.9466 | 0.9597 | 0.9466 | 0.2218 |
| 520 | 0.9662 | 0.9487 | 0.9881 | 0.9662 | 0.2863 | 0.9593 | 0.9449 | 0.9593 | 0.9449 | 0.2040 |
| 530 | 0.9659 | 0.9479 | 0.9882 | 0.9659 | 0.2855 | 0.9594 | 0.9445 | 0.9594 | 0.9445 | 0.2078 |
| 540 | 0.9657 | 0.9470 | 0.9883 | 0.9657 | 0.2532 | 0.9598 | 0.9443 | 0.9598 | 0.9443 | 0.1965 |
| 550 | 0.9657 | 0.9467 | 0.9883 | 0.9657 | 0.2993 | 0.9596 | 0.9437 | 0.9596 | 0.9437 | 0.1760 |
| 560 | 0.9662 | 0.9473 | 0.9885 | 0.9662 | 0.2605 | 0.9597 | 0.9429 | 0.9597 | 0.9429 | 0.1900 |
| 570 | 0.9667 | 0.9476 | 0.9886 | 0.9667 | 0.2515 | 0.9510 | 0.9430 | 0.9600 | 0.9430 | 0.1701 |
| 580 | 0.9670 | 0.9484 | 0.9888 | 0.9670 | 0.2206 | 0.9603 | 0.9428 | 0.9603 | 0.9428 | 0.1551 |
| 590 | 0.9676 | 0.9488 | 0.9891 | 0.9676 | 0.2071 | 0.9606 | 0.9425 | 0.9606 | 0.9425 | 0.1641 |
| 600 | 0.9680 | 0.9492 | 0.9893 | 0.9680 | 0.1979 | 0.9608 | 0.9425 | 0.9608 | 0.9425 | 0.1451 |
| 610 | 0.9684 | 0.9499 | 0.9897 | 0.9684 | 0.2104 | 0.9611 | 0.9423 | 0.9611 | 0.9424 | 0.1318 |
| 620 | 0.9689 | 0.9503 | 0.9897 | 0.9689 | 0.2008 | 0.9613 | 0.9420 | 0.9613 | 0.9420 | 0.1406 |
| 630 | 0.9692 | 0.9501 | 0.9810 | 0.9692 | 0.2160 | 0.9614 | 0.9414 | 0.9614 | 0.9414 | 0.1219 |
| 640 | 0.9695 | 0.9507 | 0.9901 | 0.9695 | 0.1826 | 0.9617 | 0.9414 | 0.9617 | 0.9414 | 0.1353 |
| 650 | 0.9706 | 0.9499 | 0.9902 | 0.9706 | 0.2080 | 0.9619 | 0.9409 | 0.9619 | 0.9409 | 0.1133 |
| 660 | 0.9708 | 0.9500 | 0.9904 | 0.9708 | 0.1751 | 0.9621 | 0.9408 | 0.9621 | 0.9408 | 0.1227 |
| 670 | 0.9707 | 0.9499 | 0.9905 | 0.9710 | 0.1708 | 0.9621 | 0.9403 | 0.9621 | 0.9403 | 0.1104 |
| 680 | 0.9709 | 0.9501 | 0.9906 | 0.9709 | 0.1904 | 0.9625 | 0.9399 | 0.9625 | 0.9399 | 0.1006 |
| 690 | 0.9709 | 0.9499 | 0.9907 | 0.9709 | 0.1833 | 0.9626 | 0.9397 | 0.9626 | 0.9397 | 0.1099 |
| 700 | 0.9707 | 0.9494 | 0.9907 | 0.9707 | 0.1588 | 0.9627 | 0.9394 | 0.9627 | 0.9394 | 0.1000 |

**Table S2:** R^2^ values and slopes after plotting the log absorbance of the supernatant samples against wavelength.

|  | *E. viscosa* JF 03-3F | | *E. viscosa* JF 03-4F | | *E. dermatitidis* | |
| --- | --- | --- | --- | --- | --- | --- |
|  | R^2^ | Slope | R^2^ | Slope | R^2^ | Slope |
|  | **YPD** | | | | | |
| Day 3 | 0.9746 | -0.0031 | 0.9792 | -0.0032 | 0.9803 | -0.0021 |
| Day 4 | 0.9727 | -0.0029 | 0.9903 | -0.0031 | 0.9914 | -0.0023 |
| Day 5 | 0.9768 | -0.0029 | 0.9778 | -0.0029 | 0.9823 | -0.0023 |
| Day 6 | 0.9774 | -0.0029 | 0.9762 | -0.0029 | 0.9810 | -0.0023 |
| Day 7 | 0.9755 | -0.0028 | 0.9778 | -0.0029 | 0.9849 | -0.0024 |
|  | **MEA** | | | | | |
| Day 3 | 0.9845 | -0.0044 | 0.9938 | -0.0029 | 0.9354 | -0.0016 |
| Day 4 | 0.9932 | -0.0029 | 0.9916 | -0.0026 | 0.9907 | -0.0026 |
| Day 5 | 0.9898 | -0.0029 | 0.9896 | -0.0023 | 0.9887 | -0.0028 |
| Day 6 | 0.9889 | -0.0029 | 0.9871 | -0.0026 | 0.9972 | -0.0026 |
| Day 7 | 0.9925 | -0.003 | 0.9891 | -0.0024 | 0.9890 | -0.0031 |

**Table S3:** Media variations tested on *E. viscosa* strains for inducing melanin excretion and their outcomes.

| Base media | Component Removed | Added nitrogen | Added carbon | Added other | Days of growth | Melanin excreted | |
| --- | --- | --- | --- | --- | --- | --- | --- |
|  |  |  |  |  |  | JF 03-3F | JF 03-4F |
| MN | N/A | 2% peptone | 2% dextrose | N/A | 16 | No | No |
| MN | NaNO_3_ | 2% peptone | 2% dextrose | N/A | 16 | No | No |
| MN | NaNO_3_ | 0.5 g/L Tyr | 2% dextrose | N/A | 16 | No | No |
| MN | Trace elements | 2% peptone | N/A | N/A | 45 | No | No |
| MN | Trace elements | 0.5 g/L Tyr | N/A | N/A | 45 | No | No |
| MEA | Peptone | 2% w/v NH_4_SO_4_ | N/A | N/A | 19 | No | No |
| MEA | Peptone | 10x AA stock | N/A | N/A | 10 | No | No |
| MEA | Peptone | 0.5 g/L Tyr | N/A | N/A | 26 | Yes | Yes |
| MEA | Peptone | 1 g/L Tyr | N/A | N/A | 26 | Yes | No |
| YPD | Peptone | 20x AA stock | N/A | N/A | 32 | Yes | Yes |
| YPD | Peptone | 2% w/v NH_4_SO_4_ | N/A | N/A | 32 | No | No |
| YPD | N/A | N/A | N/A | Trace Elements | 17 | No | No |
| BBM | N/A | 0.5 g/L Tyr | 2% dextrose | N/A | 26 | No | No |
| BBM | N/A | 20x AA | 2% dextrose | N/A | 26 | No | Yes |
| BBM | N/A | 20x AA;  0.5 g/L Tyr | 2% dextrose | N/A | 26 | No | Yes |
