# Supplementary material for: Characterization of a novel polyextremotolerant fungus, Exophiala viscosa, with insights into its melanin regulation and ecological niche
Source: G3 (Bethesda). 2023 May 23;13(8):jkad110. doi: 10.1093/g3journal/jkad110 (PMC10411609; doi:10.1093/g3journal/jkad110)
Supplement: jkad110_Supplementary_Data [file jkad110_supplementary_data.zip › Supplementary_Figure_Captions.docx]

**Figure S1:** Visual summary of the VALAP method for setting up microscope slides for long-term microscopy of budding patterns of yeast cells. VALAP is a 1:1:1 ratio by weight of Vaseline:Lanolin:Parafin which is heated to 114 °C to completely melt the mixture. After the mixture has melted, it must be maintained at 114 °C and not higher or else the mixture will become useless. To prepare the microscope slide, 6 μL of 5:1000 diluted cells was added directly onto the slide. Then a 1 cm^2^ slab of MEA agar is placed on top of the diluted cells. Additionally, 8 μL of liquid MEA is placed on top of the agar slab, and the cover slip is set on top of the liquid MEA. The warm VALAP is then applied to all of the outside edges of the coverslip using a paintbrush. Then using a needle syringe, air holes are carefully poked into the VALAP perimeter to allow for gas exchange while the cells are growing.

**Figure S2:** Rooted Bayesian phylogenetic tree of the concatenation of the 18S, 28S, ITS, and RPB1 regions of available *Exophiala* species with the addition of *E. viscosa* JF 03-3F and *E. viscosa* JF 03-4F to identify the phylogenetic location of these new species within the genus *Exophiala*. The tree with the highest log likelihood is shown. The numbers above the branches are the Bayesian posterior probability/maximum likelihood bootstrap values, with posterior probabilities > 75% and bootstrap values > 80 shown. Location of *E. viscosa* JF 03-3F and *E. viscosa* JF 03-4F within *Exophiala* places them closest to *E. sideris* with 100% posterior probability and a bootstrap value of 96.

**Figure S3:** A) *E. viscosa* JF 03-3F plate morphology; grown on an MEA plate for 10 days. B) *E. viscosa* JF 03-3F cell morphology; grown in liquid MEA for 5 days; 60x objective lens. C) *E. viscosa* JF 03-4F plate morphology; grown on a MEA plate for 10 days. D) *E. viscosa* JF 03-4F cell morphology; grown in liquid MEA for 5 days; 60x objective lens. (Both plate photos and microscopy photos were taken by Christian Elowsky)

**Figure S4:** Nile red micrographs of *E. viscosa* JF 03-3F and *E. viscosa* JF 03-4F to display their lipid bodies. Both *E. viscosa* JF 03-3F and *E. viscosa* JF 03-4F harbor lipid bodies. Cells were grown in liquid MEA for 3 days; Nile red solution was 50 μg/mL; 1 μL of Nile red was used for every 10 μL of cells. Scale bar represents scale for all images.

**Figure S5:** Synteny dot plot of *E. viscosa* JF 03-3F contigs as the target genome sequence and *E. viscosa* JF 03-4F contigs as the query genome sequence. The dot plot shows that while some of the DNA across these two strains is syntenic, there are many DNA rearrangements and a few inversions that have occurred between these two fungi. Additionally, most of the sequences are more than 75% matching.

**Figure S6:** Synteny dot plot of *E. sideris* as the target genome sequence and *E. viscosa* JF 03-3F contigs as the query genome sequence. Comparison of these two fungi shows a great deal of sequence rearrangements and inversions. The syntenic regions of these genomes are also shown to have low sequence matching <25% which is the majority of the percent matching.

**Figure S7:** Synteny dot plot of *E. sideris* as the target genome sequence and *E. viscosa* JF 03-4F contigs as the query genome sequence. Much like the genome comparison of *E. sideris* and JF 03-3F, these two fungi also show a large amount of sequence rearrangements and inversions. Syntenic regions of these genomes are shown to have low sequence matching <25% and most of the sequences are below 25% matching.

**Figure S8:** MAT loci gene order for *E. sideris*, *E. viscosa* JF 03-3F, and *E. viscosa* JF 03-4F. In all three species the same genes are present within the MAT locus. *E. sideris* is indicated to have a gene for a hypothetical protein between APN2 and MAT1-1-4, whereas *E. viscosa* JF 03-3F and *E. viscosa* JF 03-4F were not predicted to have that gene. Additionally, all three species have COX 13 and APC5 downstream of their MAT loci, but the gene order or orientation is different amongst the three species. Numbers above the genes represent their protein ID numbers in the JGI Mycocosm database. Bottom part shows a portion of the protein sequences of the MAT1-1-1 for *E. sideris*, *E. viscosa* JF 03-3F, and *E. viscosa* JF 03-4F. All three species share high homology in this protein, dark blue amino acids mean all three species have the same amino acid in that position.

**Figure S9:** Growth of *E. viscosa* JF 03-3F and *E. viscosa* JF 03-4F at the lowest and highest temperatures tested, after prolonged periods of time. Growth at 4 °C continued for a year in both strains, indicating that they can grow at these lower temperatures for extended periods of time. Additionally, we observed that while neither species was capable of active growth at 37 °C, 48 hours was not too long of an exposure time to kill these cells. Whereas, at 42 °C neither strain was capable of growth and both were killed after 48 hours of exposure.

**Figure S10:** Lipid profile of *S. cerevisiae* (Sc), *E. dermatitidis* (Ed), *E. viscosa* JF 03-3F (Ev), and *E. viscosa* JF 03-4F (El) using four different medias (1: MEA, 2: MEA + 2% peptone, 3: MEA + 2% glycerol, 4: MEA + 2% glycerol + 2% peptone). Differences in fermentable vs. non-fermentable carbon sources and amount of nitrogen source did not alter the amount or types of lipids produced by either *E. viscosa* JF 03-3F or *E. viscosa* JF 03-4F. These fungi also showed no unique lipid production or any extreme accumulations of any lipids when compared to other fungi. Lipid codes: PI = Phosphotidylinositol; PS = Phosphotidylserine; PC = Phosphotidylcholine; PE = Phosphotidylethanolamine; MAG = Monoacylglycerol; DAG = Diacylglycerol; TAG = Triacylglycerol.

**Figure S11:** (A) *E. viscosa* JF 03-3F and *E. viscosa* JF 03-4F lawns grown on MEA media contain either 100 μg/mL of kojic acid, 100 μg/mL of phthalide, or both in hopes of blocking melanin production through chemical means. Neither the individual melanin blockers nor their combined efforts were able to block melanin production in either fungus. (B) The same chemicals were used to attempt a different method of melanin blocking but at different concentrations, 10 mg/mL for kojic acid and 10 mM for phthalide. These compounds were added to filter discs and placed on lawns of *E. viscosa* JF 03-3F and *E. viscosa* JF 03-4F. Neither the individual compounds nor the combined compounds blocked melanin production.

**Figure S12:** *E. viscosa* JF 03-3F and *E. viscosa* JF 03-4F grown on MEA and YPD with different concentrations of peptone. *E. viscosa* JF 03-3F is capable of melanin excretion on MEA with 2% peptone, which is the same amount of peptone in regular YPD. *E. viscosa* JF 03-4F was not as prolific at excreting melanin in the MEA + 2% peptone, but there is a slight amount of excreted melanin. *E. viscosa* JF 03-3F was also capable of excreting melanin on YPD with 0.2% peptone, indicating that yeast extract might have more available nitrogen than malt extract.

**Figure S13:** *E. viscosa* JF 03-3F, *E. viscosa* JF 03-4F, and the albino mutant of *E. viscosa* JF 03-4F EMS 2-11, grown on Tyrosine media (Tyr media: 2% dextrose; 1% peptone; 0.1% yeast extract (Jalmi et al., 2012)) with or without tyrosine added at 0.5 g/L and 1 g/L, and on MN-Nitrate 2% dextrose +0.5 g/L tyrosine; day 19 of growth. This was done to determine if the tyrosine in peptone could be causing the melanin excretion, or if tyrosine alone can induce melanin excretion. If tyrosine alone was causing the excretion via the tyrosine-dependent biosynthetic pathways, then EMS 2-11 would also be excreting melanin since it is only mutated in pks1 which affects allomelanin production and does not require tyrosine as a precursor. While adding 0.5 g/L of tyrosine does seem to induce melanin excretion, adding 1 g/L seems to reduce melanin excretion. Growth on tyrosine as a sole nitrogen source also does not seem to induce melanin excretion in either the wild type strains or the albino mutant. The peptone vs. tyrosine melanin excretion phenomenon, and the melanin excretion phenomenon overall is still being studied.

**Figure S14:** Extraction of melanin from supernatants of *E. viscosa* JF 03-3F and *E. viscosa* JF 03-4F using both enzymatic and chemical methods described in (Pralea et al., 2019). Enzymatic extraction methods were incapable of extracting all the melanin, leaving behind a dark supernatant in the last step. However, melanin extracted by chemical extraction methods had complete extraction of the secreted melanin.

**Figure S15:** Log absorbance values of the extracted melanin from the supernatant of *E. viscosa* JF 03-3F. All R2 values of both MEA and YPD from every day are R2 ≥ 0.97, meaning that the compound that was extracted was melanin for all samples.

**Figure S16:** Log absorbance values of the extracted melanin from the supernatant of *E. viscosa* JF 03-4F. All R2 values of both MEA and YPD from every day are R2 ≥ 0.97, meaning that the compound that was extracted was melanin for all samples.

**Figure S17:** Log absorbance values of the extracted melanin from the supernatant of E. dermatitidis. All R2 values of both MEA and YPD from every day are R2 ≥ 0.97, meaning that the compound that was extracted was melanin for all samples.

**Figure S18**: Original unedited photos of *E. viscosa* JF 03-3F and *E. viscosa* JF 03-4F on different media types.
