# Supplementary figures and images for: Characterization of a novel polyextremotolerant fungus, Exophiala viscosa, with insights into its melanin regulation and ecological niche
Source: G3 (Bethesda). 2023 May 23;13(8):jkad110. doi: 10.1093/g3journal/jkad110 (PMC10411609; doi:10.1093/g3journal/jkad110)

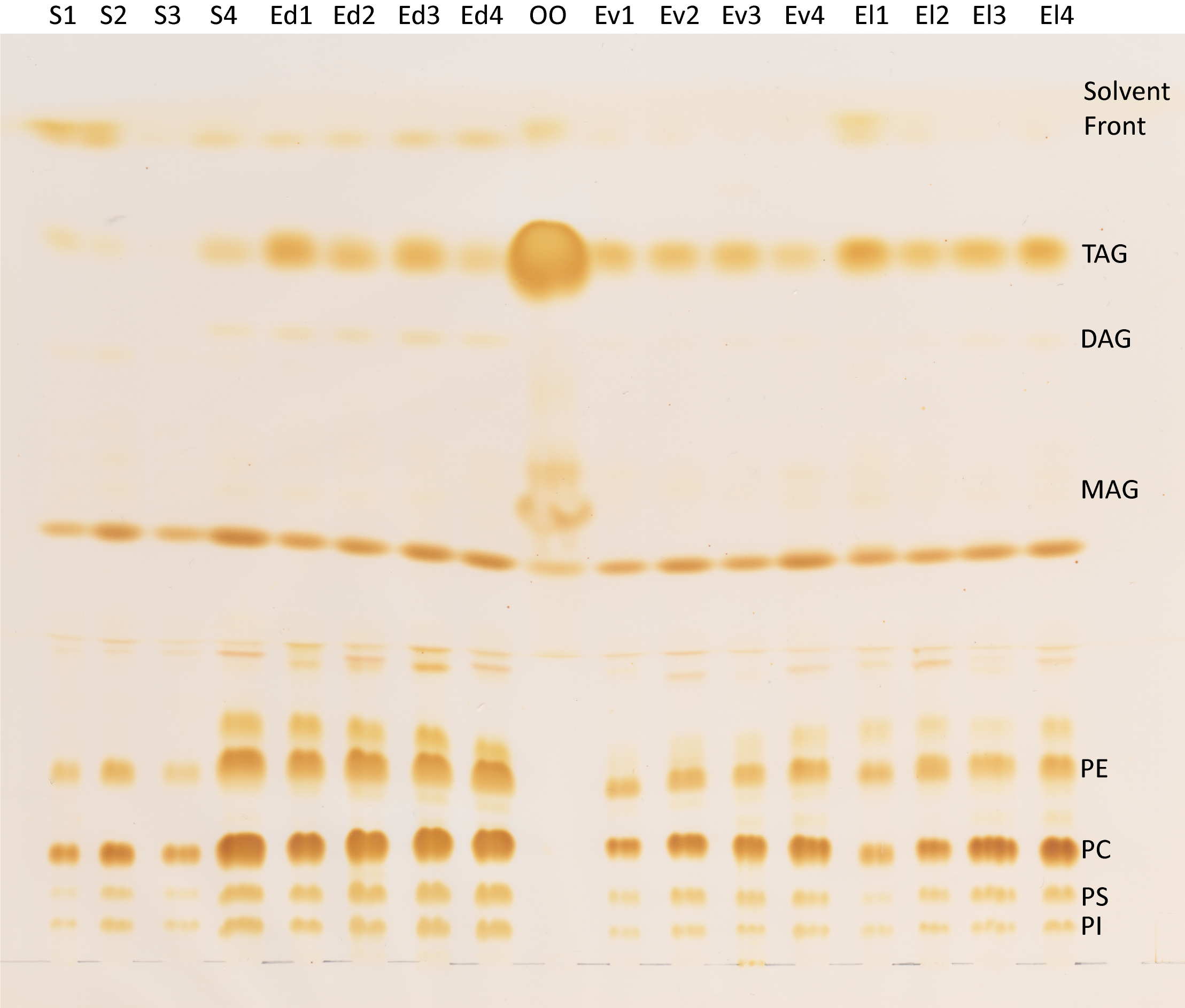

Supplement: jkad110_Supplementary_Data [file jkad110_supplementary_data.zip › Figure_S10.bmp]

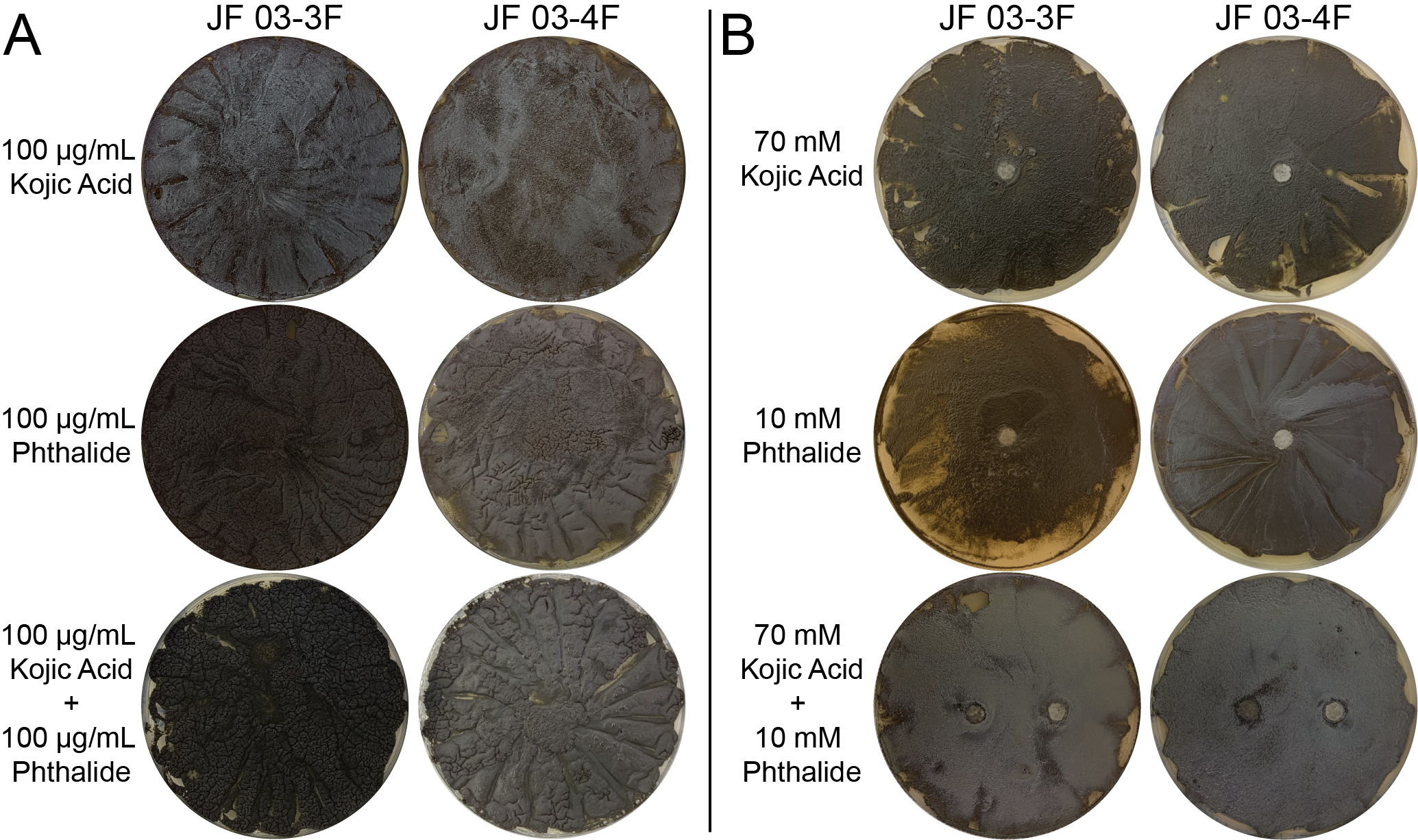

Supplement: jkad110_Supplementary_Data [file jkad110_supplementary_data.zip › Figure_S11.png]

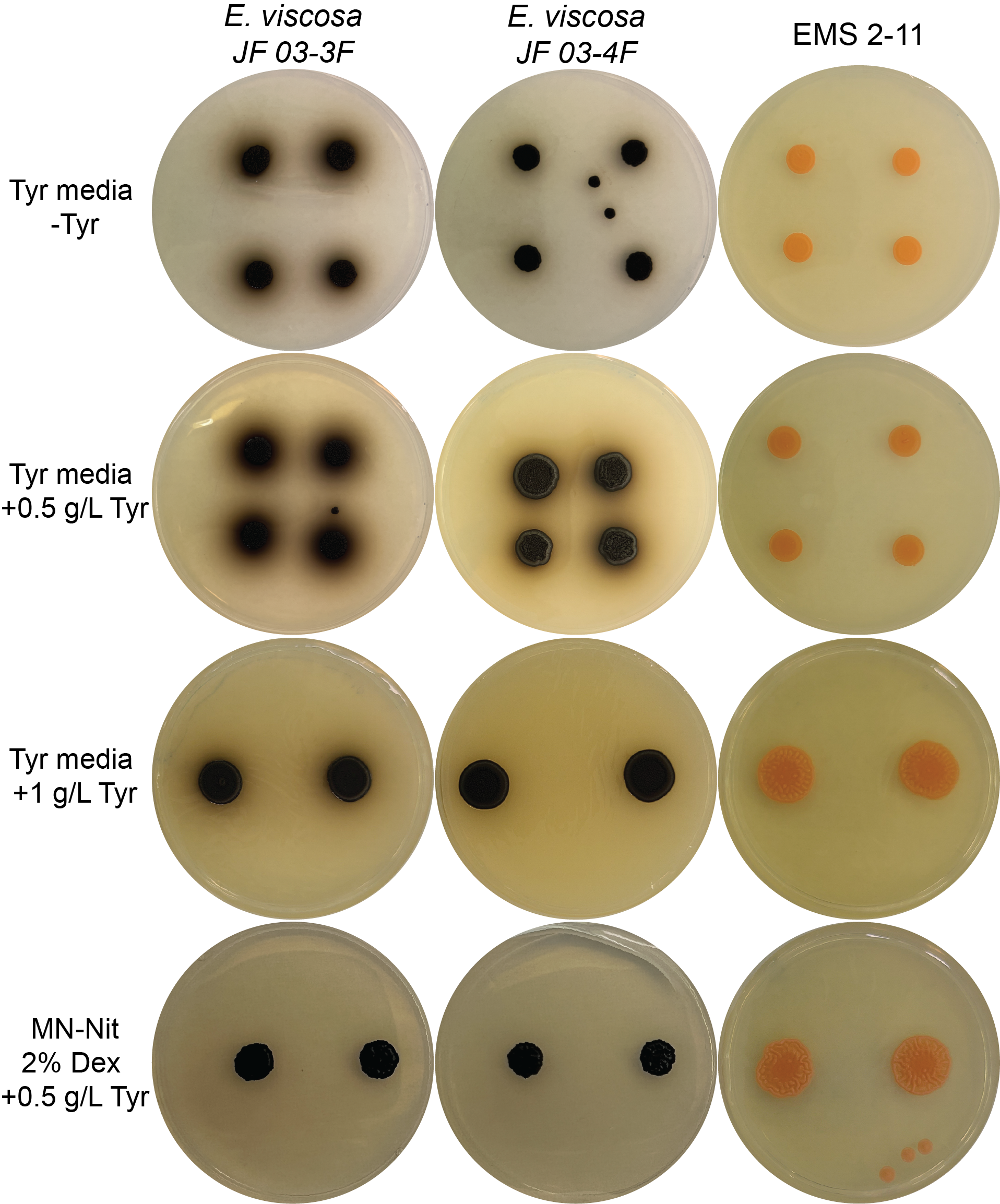

Supplement: jkad110_Supplementary_Data [file jkad110_supplementary_data.zip › Figure_S13.png]

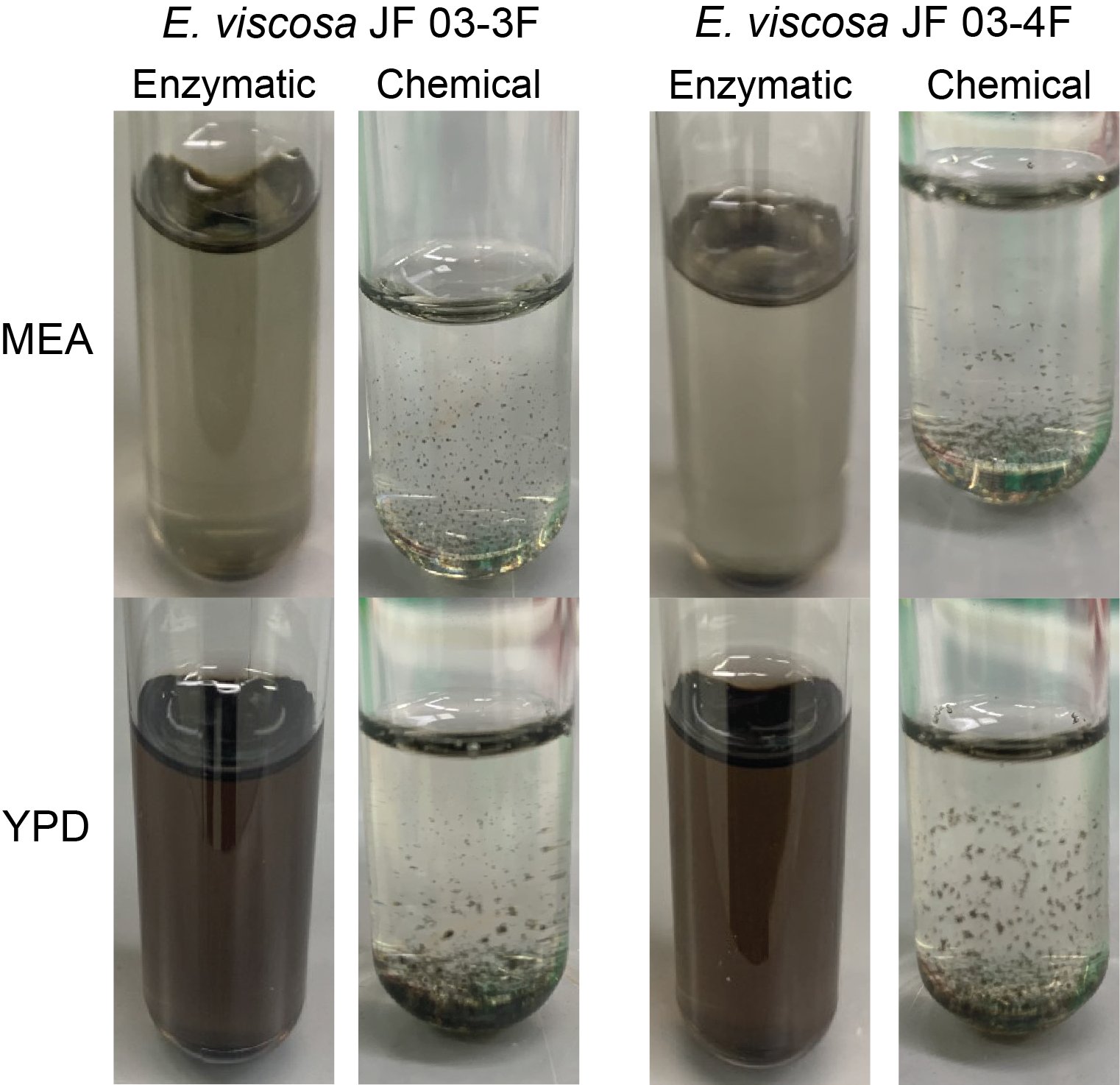

Supplement: jkad110_Supplementary_Data [file jkad110_supplementary_data.zip › Figure_S14.png]

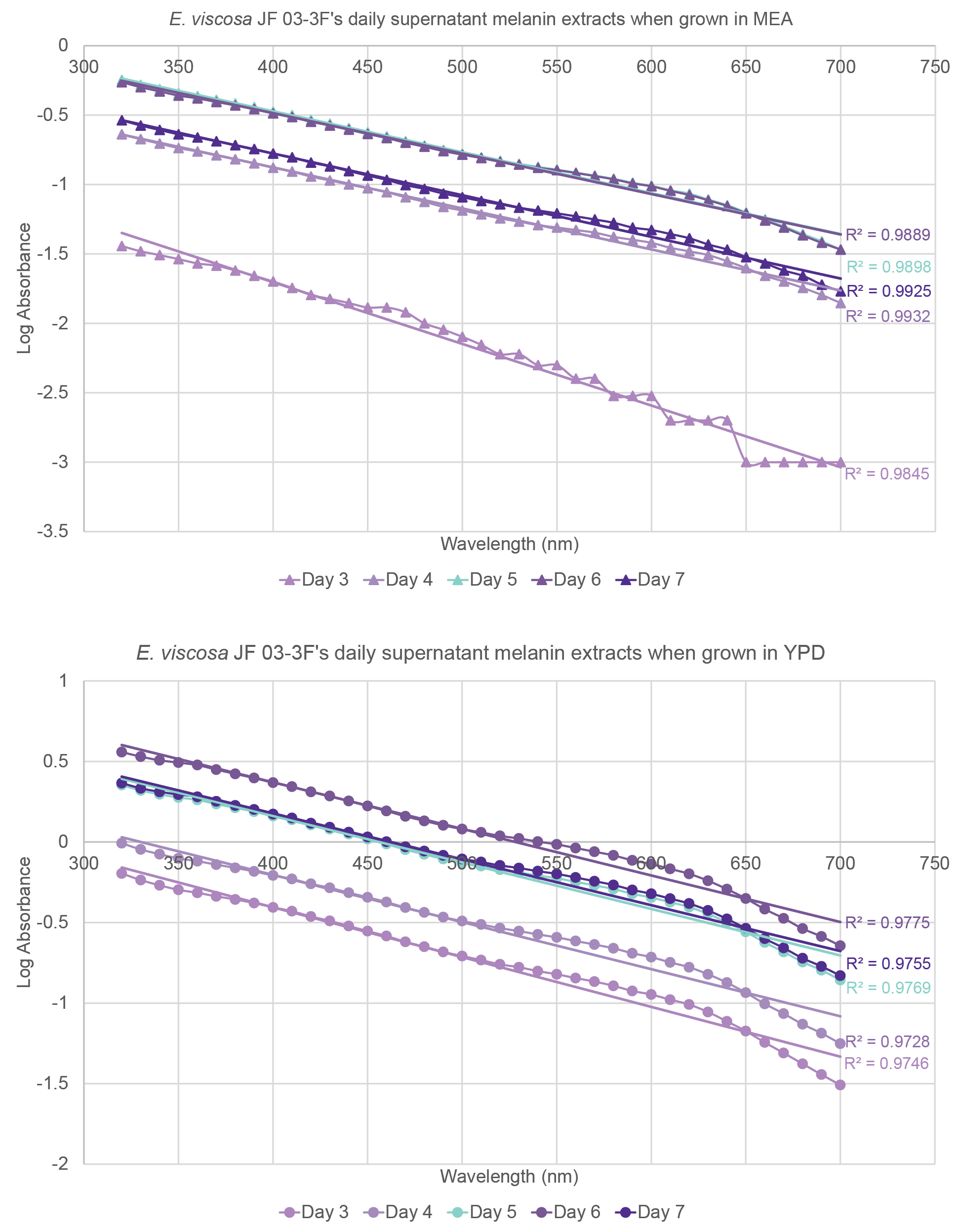

Supplement: jkad110_Supplementary_Data [file jkad110_supplementary_data.zip › Figure_S15.png]

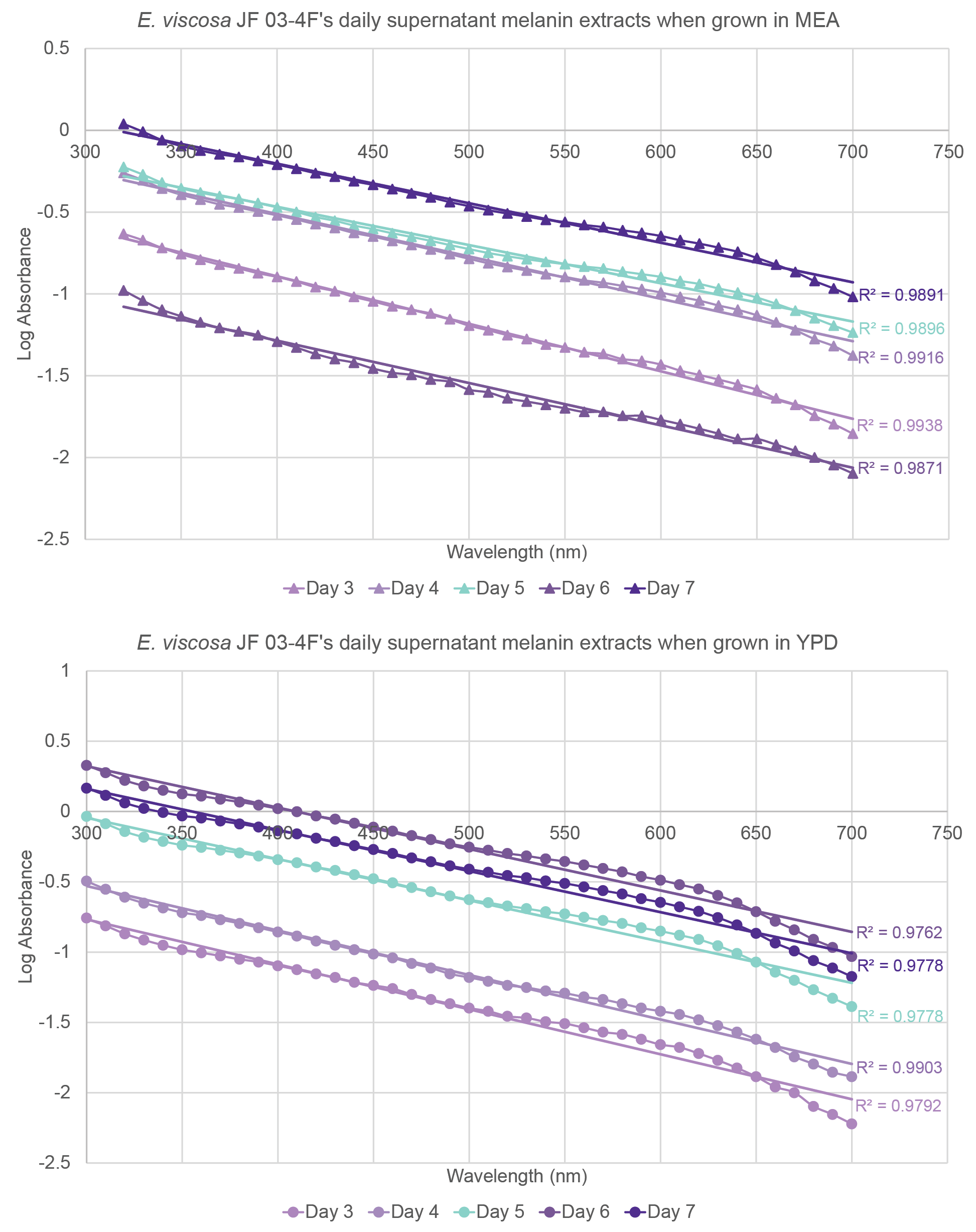

Supplement: jkad110_Supplementary_Data [file jkad110_supplementary_data.zip › Figure_S16.png]

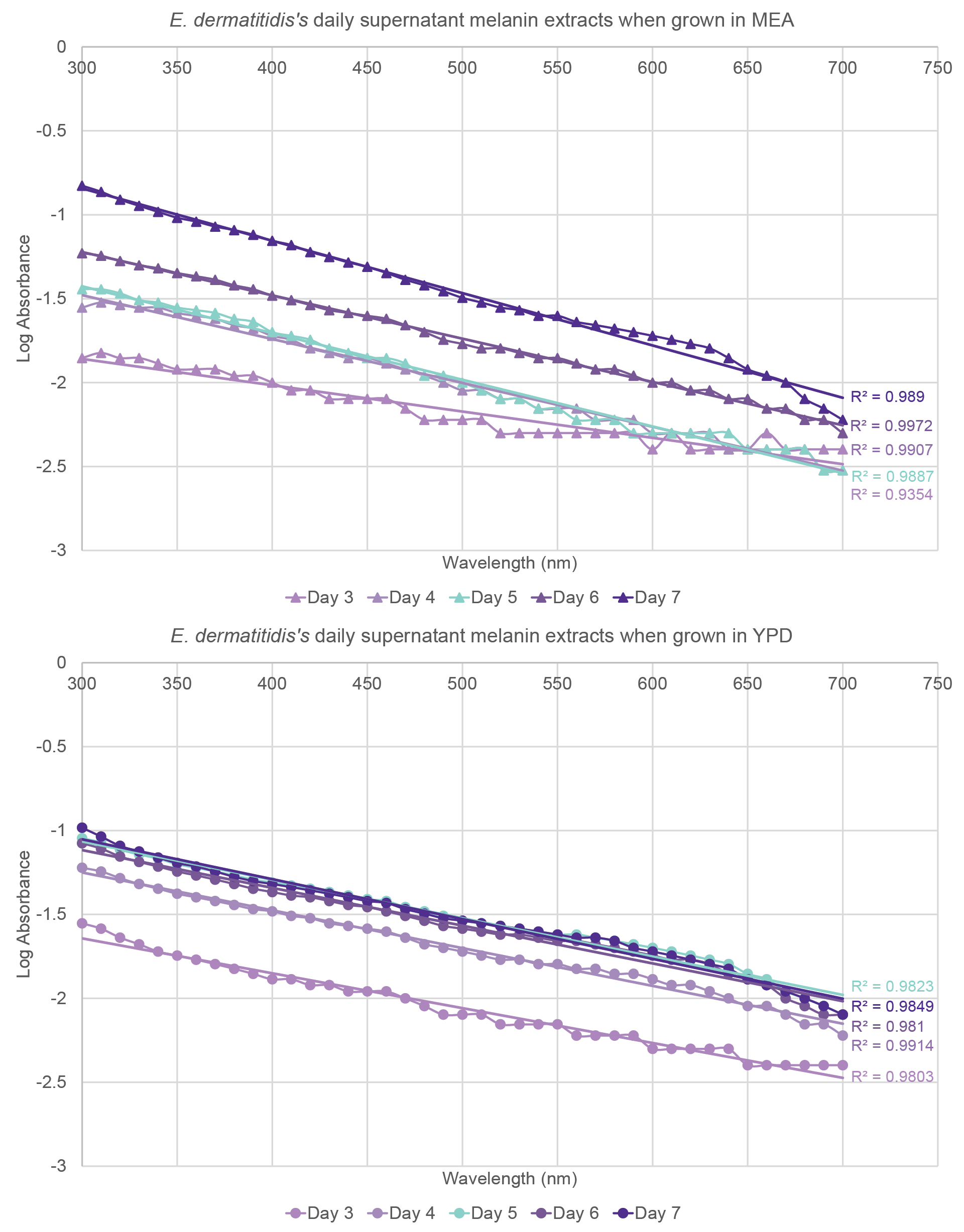

Supplement: jkad110_Supplementary_Data [file jkad110_supplementary_data.zip › Figure_S17.png]

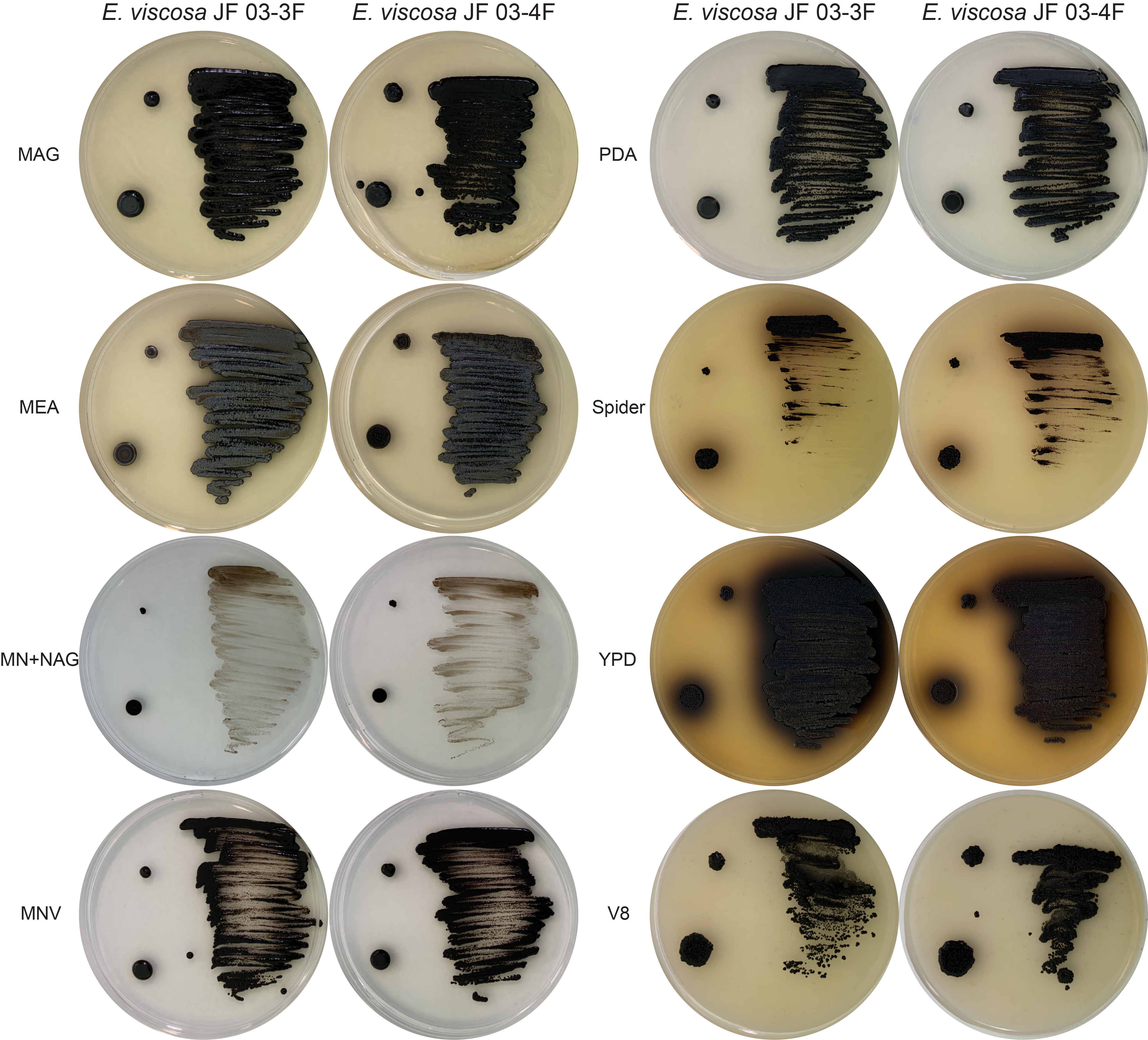

Supplement: jkad110_Supplementary_Data [file jkad110_supplementary_data.zip › Figure_S18.png]

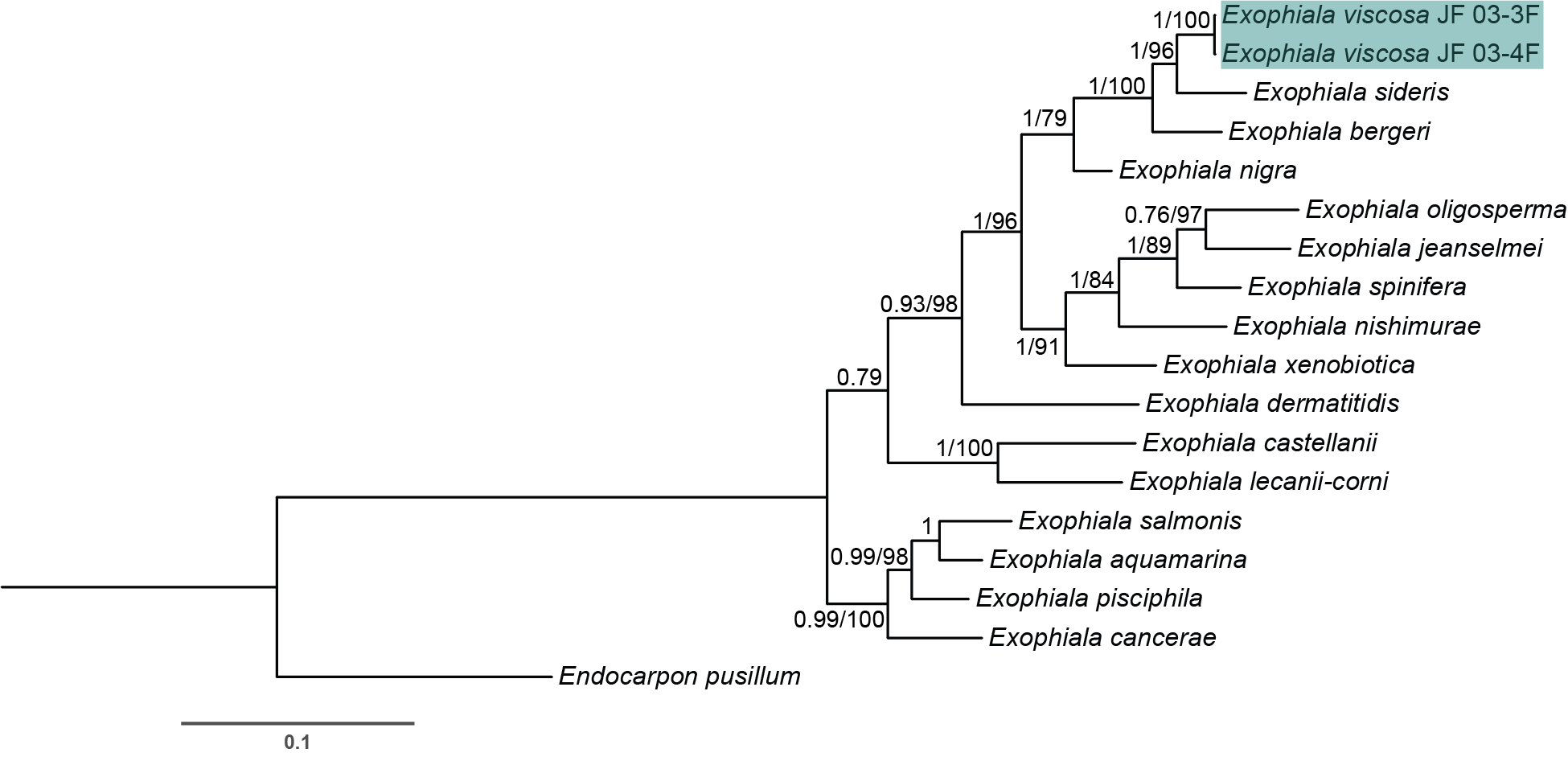

Supplement: jkad110_Supplementary_Data [file jkad110_supplementary_data.zip › Figure_S2.png]

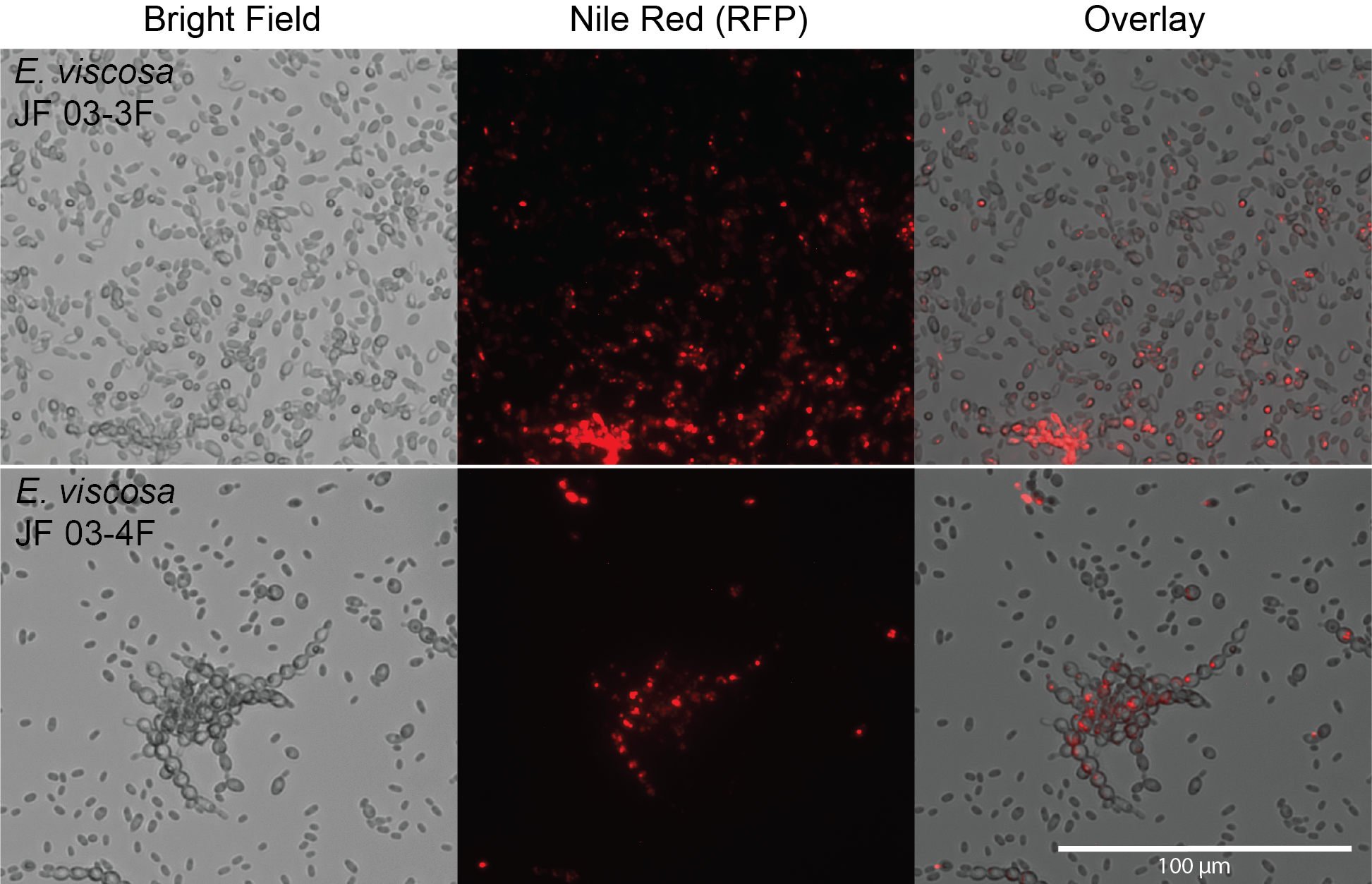

Supplement: jkad110_Supplementary_Data [file jkad110_supplementary_data.zip › Figure_S4.png]

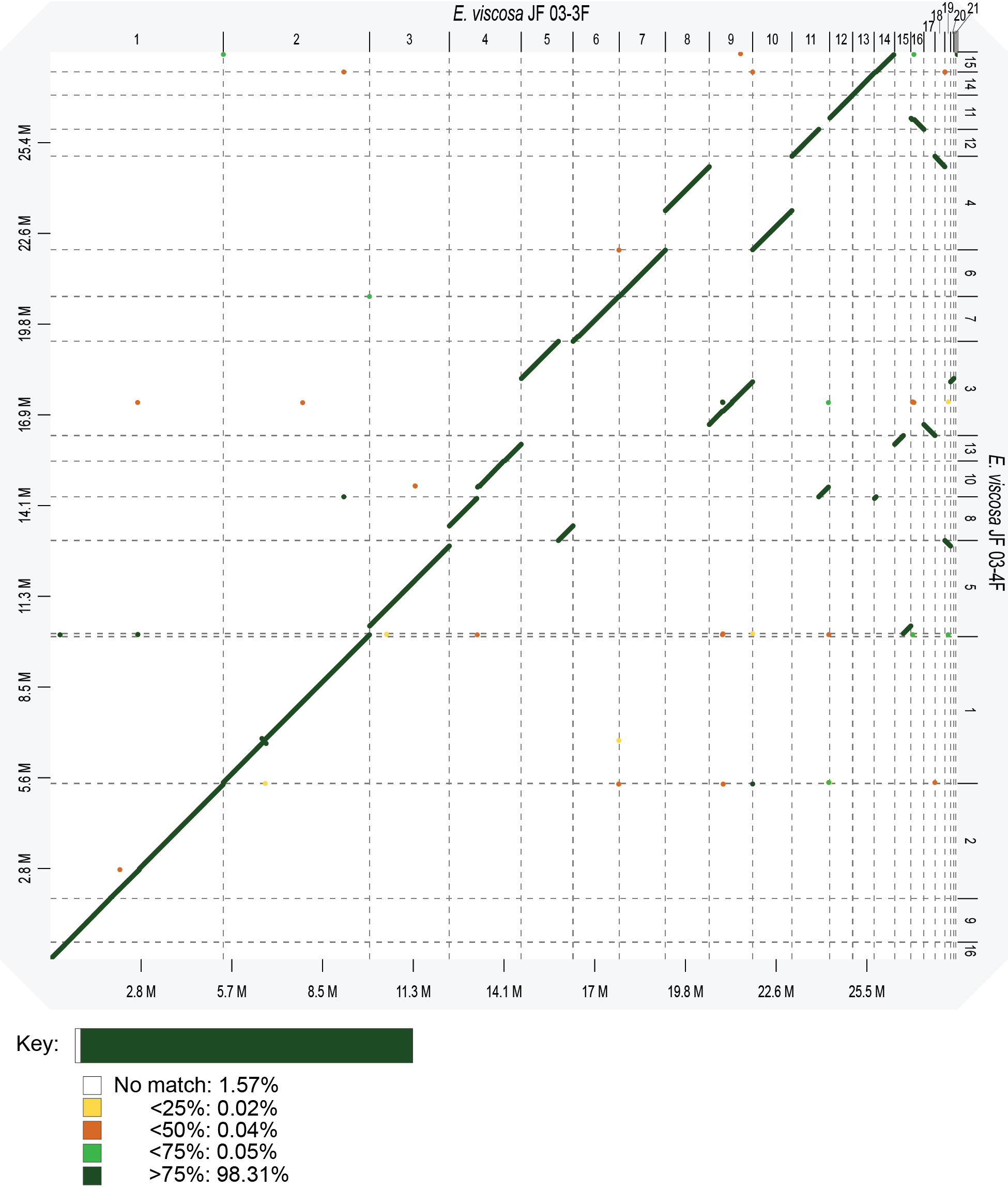

Supplement: jkad110_Supplementary_Data [file jkad110_supplementary_data.zip › Figure_S5.png]

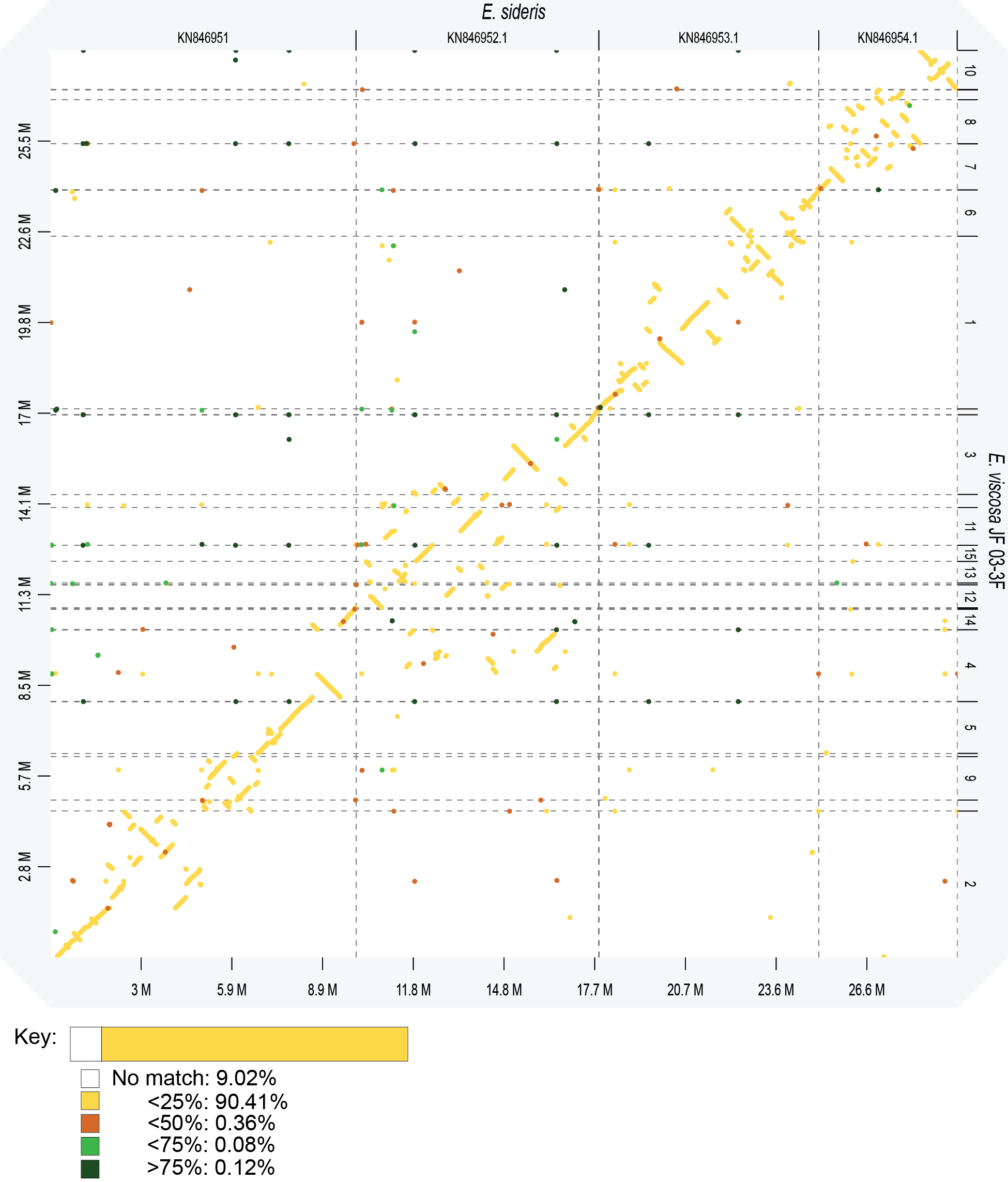

Supplement: jkad110_Supplementary_Data [file jkad110_supplementary_data.zip › Figure_S6.png]

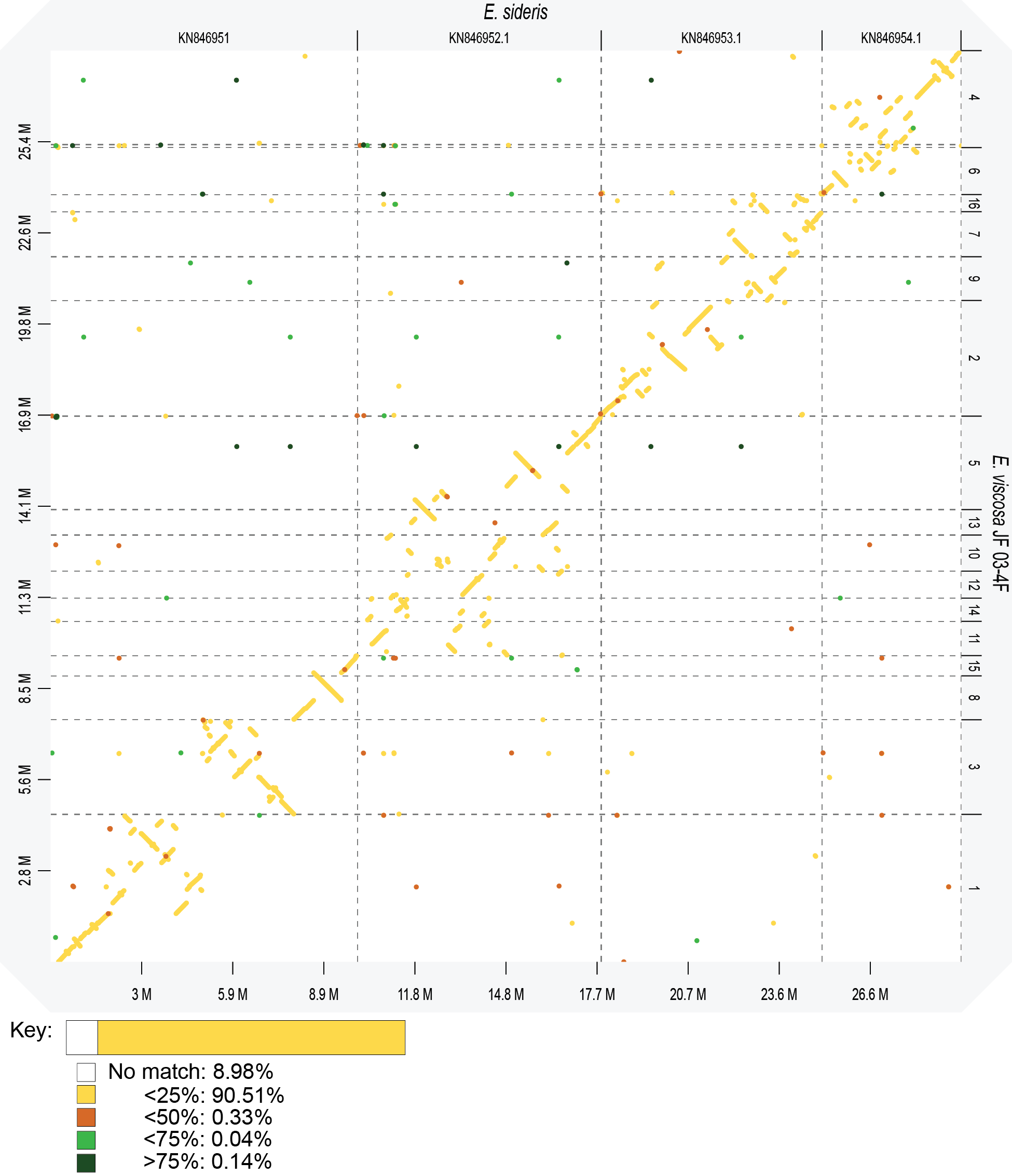

Supplement: jkad110_Supplementary_Data [file jkad110_supplementary_data.zip › Figure_S7.png]

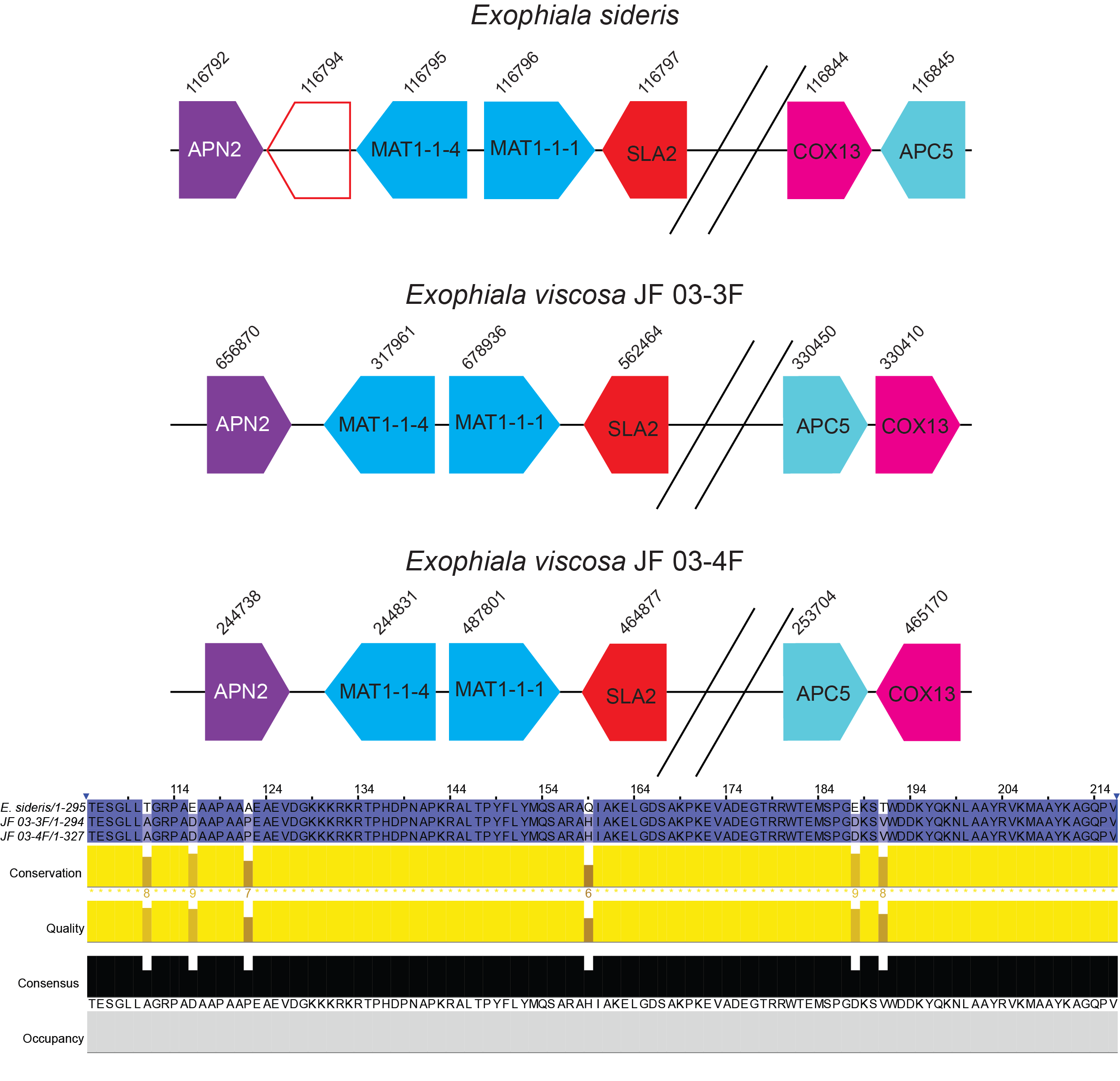

Supplement: jkad110_Supplementary_Data [file jkad110_supplementary_data.zip › Figure_S8.png]

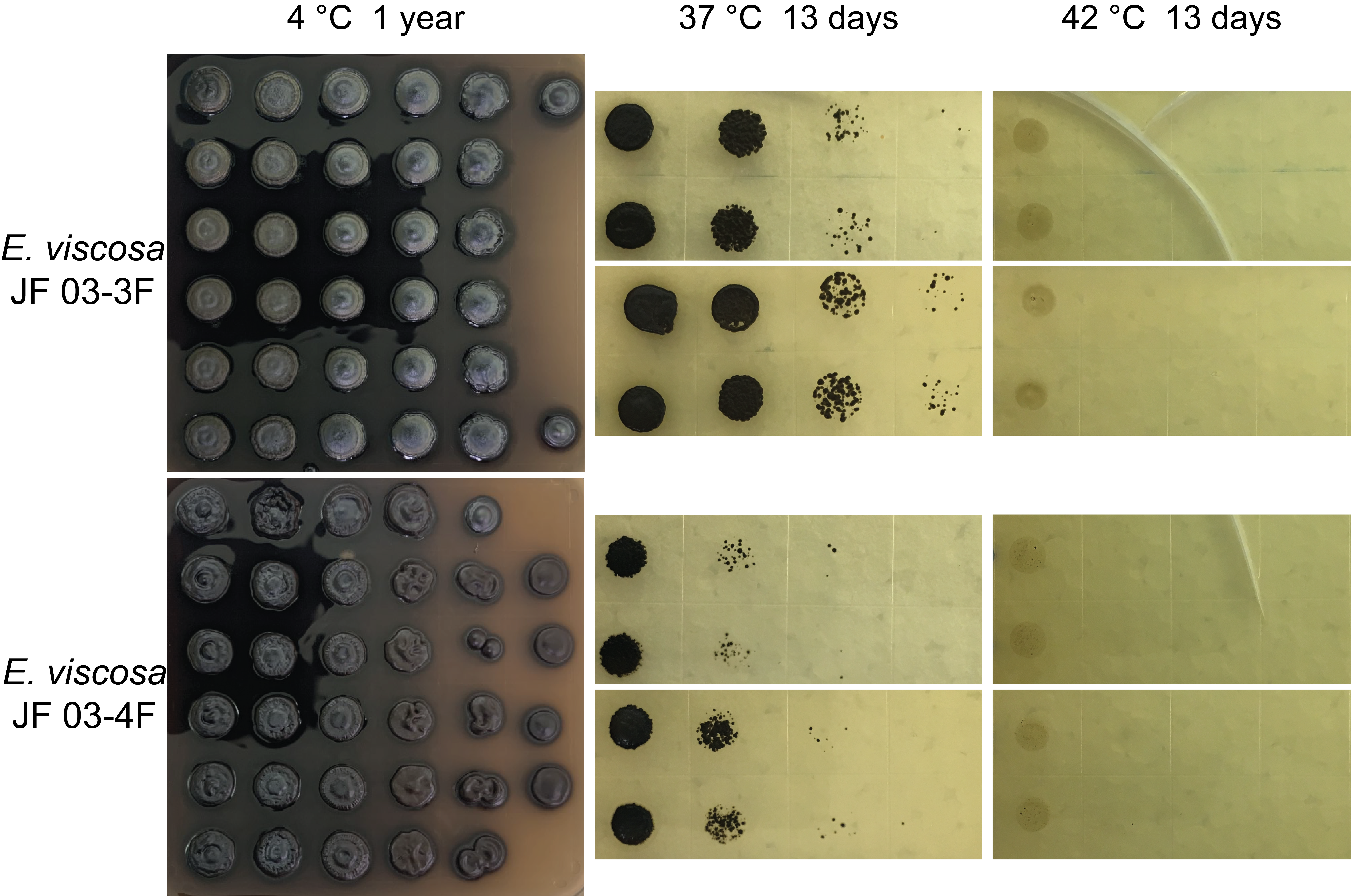

Supplement: jkad110_Supplementary_Data [file jkad110_supplementary_data.zip › Figure_S9.png]
